# Targeting caspase-8/c-FLIP_L_ heterodimer in complex II promotes DL-mediated cell death

**DOI:** 10.3389/fcell.2024.1471216

**Published:** 2024-09-30

**Authors:** Laura K. Hillert-Richter, Corinna König, Nikita V. Ivanisenko, Dirk Reinhold, Inna N. Lavrik

**Affiliations:** ^1^ Translational Inflammation Research, Medical Faculty, Otto von Guericke University, Magdeburg, Germany; ^2^ Institute of Molecular and Clinical Immunology, Medical Faculty, Otto von Guericke University, Magdeburg, Germany

**Keywords:** cell death, complex II, c-FLIP, FLIPin, caspase-8, SMAC mimetic, CD95

## Abstract

Death receptor (DR) networks are controlled by the assembly of the Death-Inducing Signaling Complex (DISC) and complex II. The family of small molecules FLIPins (FLIP interactors) were developed to target the caspase-8/c-FLIP_L_ heterodimer. FLIPin compounds were shown to promote apoptosis and caspase-8 activation at the DISC upon stimulation with death ligands (DLs) such as CD95L and TRAIL. To further investigate the role of FLIPin compounds in the DL-mediated cell death response, we analyzed their effects in combination with DLs and SMAC mimetics treatment. FLIPins were found to enhance cell viability loss and cell death induced by DL and SMAC mimetics in acute myeloid leukemia (AML), colon and pancreatic cancer cells. FLIPins enhanced both DL/BV6-induced apoptosis and DL/BV6/zVAD-fmk-induced necroptosis via an increase in complex II formation. Our results indicate that targeting the caspase-8/c-FLIP_L_ heterodimer plays a prominent role in enhancing cell death induced by co-stimulation of DL/SMAC mimetics and opens new therapeutic strategies for targeting DR networks.

## Introduction

Activation of death receptors (DRs) by death ligands (DLs) induces apoptosis or necroptosis in sensitive cells (Lavrik and Krammer, 2012; Nagata, 1999). CD95/Fas and TRAILRs are among the best studied DRs (Seyrek et al., 2022). Stimulation of the DRs CD95/TRAILRs by CD95L/TRAIL results in the formation of a death-inducing signaling complex (DISC), which comprises DR, FADD, procaspase-8/-10 and c-FLIP (Lavrik and Krammer, 2012; Sprick et al., 2002). Procaspase-8 is activated via dimerization at the DISC in Death Effector Domain (DED) filaments formed via DED interactions of FADD, procaspase-8/10 and c-FLIP (Schleich et al., 2012; Dickens et al., 2012; Fu et al., 2016). Activation of caspase-8 at the DED filaments leads to induction of the caspase cascade followed by the demolition of the cell.

Upon DR stimulation procaspase-8 can also be activated at complex IIa or RIPoptosome. The RIPoptosome is formed upon deubiquitinylation of the kinase RIPK1 and is composed of RIPK1, FADD, procaspase-8/-10 and c-FLIP proteins (Feoktistova et al., 2011; Tenev et al., 2011). RIPoptosome is a RIPK1-dependent intracellular macromolecular platform that mediates apoptosis, while upon inhibition of caspase activity and in the presence of RIPK3, it forms complex IIb/necrosome, which mediates necroptosis. Hence, RIPK1-mediated platforms promote both caspase-dependent and -independent programmes, apoptosis and necroptosis, via complex IIa/ripoptosome or complex IIb/necrosome, respectively (Silke et al., 2015; Grootjans et al., 2017). The important question that defines the plasticity of the response towards DL stimulation is the dynamics of the formation of the DISC versus complex II, which has not been really clarified so far (Matveeva et al., 2019; Lalaoui et al., 2020).

Caspase-8 activation at the DISC and RIPoptosome is controlled by c-FLIP proteins (Feoktistova et al., 2011; Ozturk et al., 2012). Three c-FLIP isoforms, have been characterized so far: c-FLIP_L_, c-FLIP_S_, and c-FLIP_R_. All three isoforms contain two DED domains. c-FLIP_L_ also contains catalytically inactive caspase-like domains (p20 and p12) at its C-terminal part (Ivanisenko et al., 2022). The two short isoforms, c-FLIP_S_ and c-FLIP_R_, inhibit procaspase-8 activation by limiting the growth of DED filament (Hughes et al., 2016). c-FLIP_L_ at the DISC can act both in a pro- and anti-apoptotic way (Hughes et al., 2016; Chang et al., 2002; Micheau et al., 2002; Fricker et al., 2010; Yu et al., 2009; Hillert et al., 2020a). The pro-apoptotic function of c-FLIP_L_ has been reported to be mediated by the formation of procaspase-8/c-FLIP_L_ heterodimers, with the active center of caspase-8 being stabilized in the active conformation through interactions with the C-terminal part of c-FLIP_L_.

**FIGURE 1:**
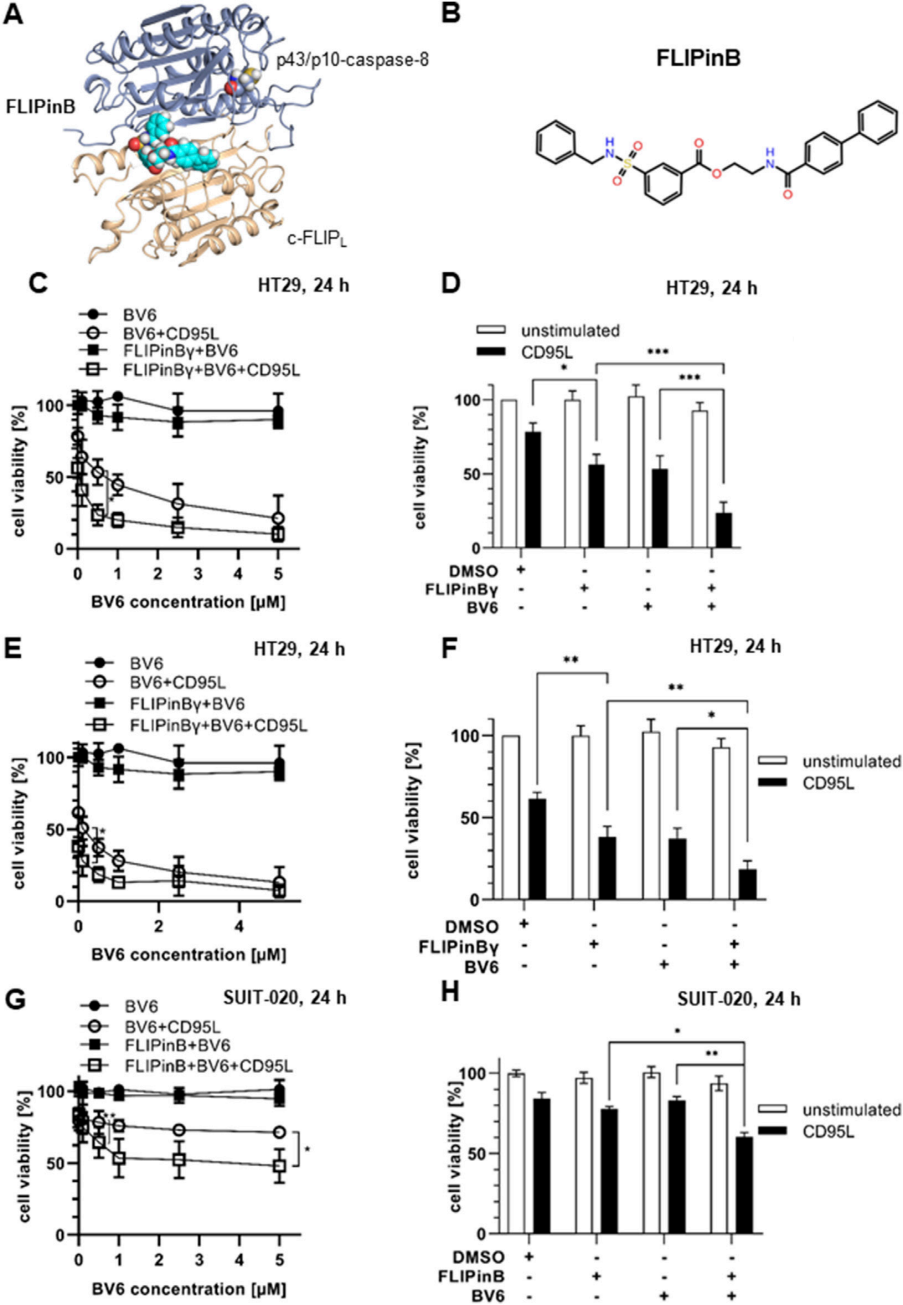
CD95L/BV6/FLIPin co-treatment increases the loss of cell viability in cancer cell lines **(A)** FLIPinB bound to the caspase-8/c-FLIP_L_ heterodimer. **(B)** The structure of FLIPinB. **(C, E, G)** HT29 cells **(C, E)** or SUIT-020 cells **(G)** were pretreated for 2 h with 20 µM FLIPinBγ **(C, E)** or 30 µM FLIPinB **(G)** and 1 h pre-stimulation with BV6 (concentration range 0.1 µM, 0.5 µM, 1 μM, 2.5 µM, 5 µM) and then stimulated for 22 h with 250 ng/mL **(C)** or 500 ng/mL CD95L **(E, G)**. **(D, F, H)** HT29 cells **(D, F)** or SUIT-020 cells **(H)** were pretreated with 20 µM FLIPinBγ **(D, F)** or 30 µM FLIPinB **(H)** for 2 h and pre-stimulated with 0. 5 μM **(D, F)** or 5 µM **(H)** BV6 and then stimulated with 250 ng/mL **(D)** or 500 ng/mL CD95L **(F, H)** for 22 h. Cell viability was measured using the Cell Titer-Glo^®^ Luminescent Cell Viability Assay. The mean and standard deviation of three independent experiments are shown. For statistical analysis, values were calculated using unpaired one-way ANOVA test with Tukey *post hoc* test. The following values were used: ****p < 0.0001,***p < 0.001; **p < 0.01; *p < 0.05; ns not significant.

Recently, small molecules thatbind to the C-terminal part of c-FLIP_L_ at the interface of the caspase-8/c-FLIP_L_ heterodimer have been rationally designed (Hillert et al., 2020b). These compounds, FLIPins (FLIP interactors), were constructed to stabilize the active center of caspase-8 in the caspase-8/c-FLIP_L_ heterodimer (Figures 1A, B). In particular, the proapoptotic effects of the caspase-8/c-FLIP_L_ heterodimer are based on the interactions of the C-terminal part of c-FLIP_L_ with the part of caspase-8 including the L2 loop and the resulting stabilization of the caspase-8 active center in the active conformation (Micheau et al., 2002; Yu et al., 2009; Smyth et al., 2020). The L2 loop is cleaved in the course of apoptosis, which leads to the abrogation of stabilization effects on the active center of caspase-8 within the caspase-8/c-FLIP_L_ heterodimer. FLIPins were designed to bind to the interface the C-terminal fragments of caspase-8 and c-FLIP_L_ and to mimic the cleaved off fragment of the L2 loop and thereby stabilizes the caspase-8 active center (Figures 1A, B). The prototype compounds, FLIPinB and its water-soluble version FLIPinBγ enhanced caspase-8 activity at the DISC and CD95L/TRAIL-induced apoptosis (Hillert et al., 2020b). Moreover, FLIPinB has shown prominent effects on the sensitization of cancer cells towards DL-induced apoptosis upon co-treatment with Mcl-1 inhibitor (Konig et al., 2020). FLIPins enhance the caspase-8 activity at the DISC in the first hours after DR stimulation and thereby promote apoptosis (Hillert et al., 2020b).

In this study, to further explore the effects of FLIPin compounds, we examined their effects on co-stimulation with DL and the SMAC mimetic BV6. BV6 was selected as it is well established in several *in vitro* studies, although there are a number of recently developed SMAC mimetics being investigated in clinical trials (Morrish et al., 2020). FLIPins were found to be efficient in enhancing cell death induced by DL/BV6. This was the case for both FLIPinB and its water-soluble version FLIPinBγ. In addition, FLIPin compounds enhanced the formation of complex II, which in turn promoted the cell death. These findings provide new insights into the role of the caspase-8/c-FLIP_L_ heterodimer in these cell death pathways and open new avenues for the development of anti-cancer therapeutics.

## Results

### FLIPin compounds enhance CD95L/SMAC mimetic-mediated cell viability loss

Previous studies have shown that treatment with the SMAC mimetic BV6 sensitizes the cells towards DL-mediated cell death (Feoktistova et al., 2011; Geserick et al., 2009). To investigate the effects of FLIPin compounds in combination with BV6 and CD95L/BV6 treatment, we utilized the colon cancer cell line HT29 and the pancreatic cancer cell line SUIT-020. We aimed on testing both compounds, FLIPinB and its water-soluble version FLIPinBγ, termed thereafter as FLIPin compounds or FLIPins, because in the previous studies we have shown that they have the similar activity (Ivanisenko et al., 2022; Konig et al., 2020). HT29 cells were treated with BV6/FLIPin or CD95L/BV6/FLIPin followed by cell viability analysis (Figures 1C–F). Cell viability was analyzed by measuring cellular ATP, a well-established method for determining cell viability (Konig et al., 2020). Almost no cell viability loss was detected upon the treatment with BV6 alone or the BV6/FLIPin combination. However, FLIPin compounds enhanced the cell viability loss upon CD95L/BV6 treatment (Figures 1C–F). These effects were observed upon treatment with different doses of BV6 and CD95L (Figures 1C–F). FLIPin compounds also enhanced the effects of CD95L/BV6 on cell viability loss upon stronger CD95L stimulation, although these effects were less pronounced due to the high rate of cell viability loss but still significant (Figures 1E, F).

In the pancreatic cancer cell line SUIT-020, FLIPin compounds also enhanced the loss of cell viability induced by CD95L/BV6 treatment (Figures 1G, H). It should be noted that SUIT-020 cells were less sensitive to BV6 or CD95L/BV6 treatments compared to HT29 cells; however, FLIPins also enhanced the loss of cell viability, particularly upon CD95L/BV6 treatment, in SUIT-020 cells (Figures 1G, H). Moreover, BV6 and CD95L/FLIPin acted in the synergistic manner in SUIT-020 and HT29 cells (Supplementary Figures S1A, S1B).

Having tested the effects of FLIPins on HT29 and SUIT-020 cells, the effects of DL/BV6/FLIPin treatments on human primary fibroblasts were examined (Supplementary Figures S1C, S1D). Primary fibroblasts were much less sensitive towards TRAIL/BV6/FLIPin and CD95L/BV6/FLIPin treatments, although some minor effects on cell viability upon stimulation with higher concentrations of CD95L and BV6 were observed upon short-term treatment. No sensitization of primary fibroblasts was observed upon long-term treatment in contrast to pancreatic and colon cancer cells. These analyses strongly suggest that FLIPin compounds ameliorate DL/BV6-induced loss of cell viability in colon and pancreatic cancer cells.

### FLIPin compounds enhance CD95L/BV6-induced cell viability loss of AML cells

**FIGURE 2:**
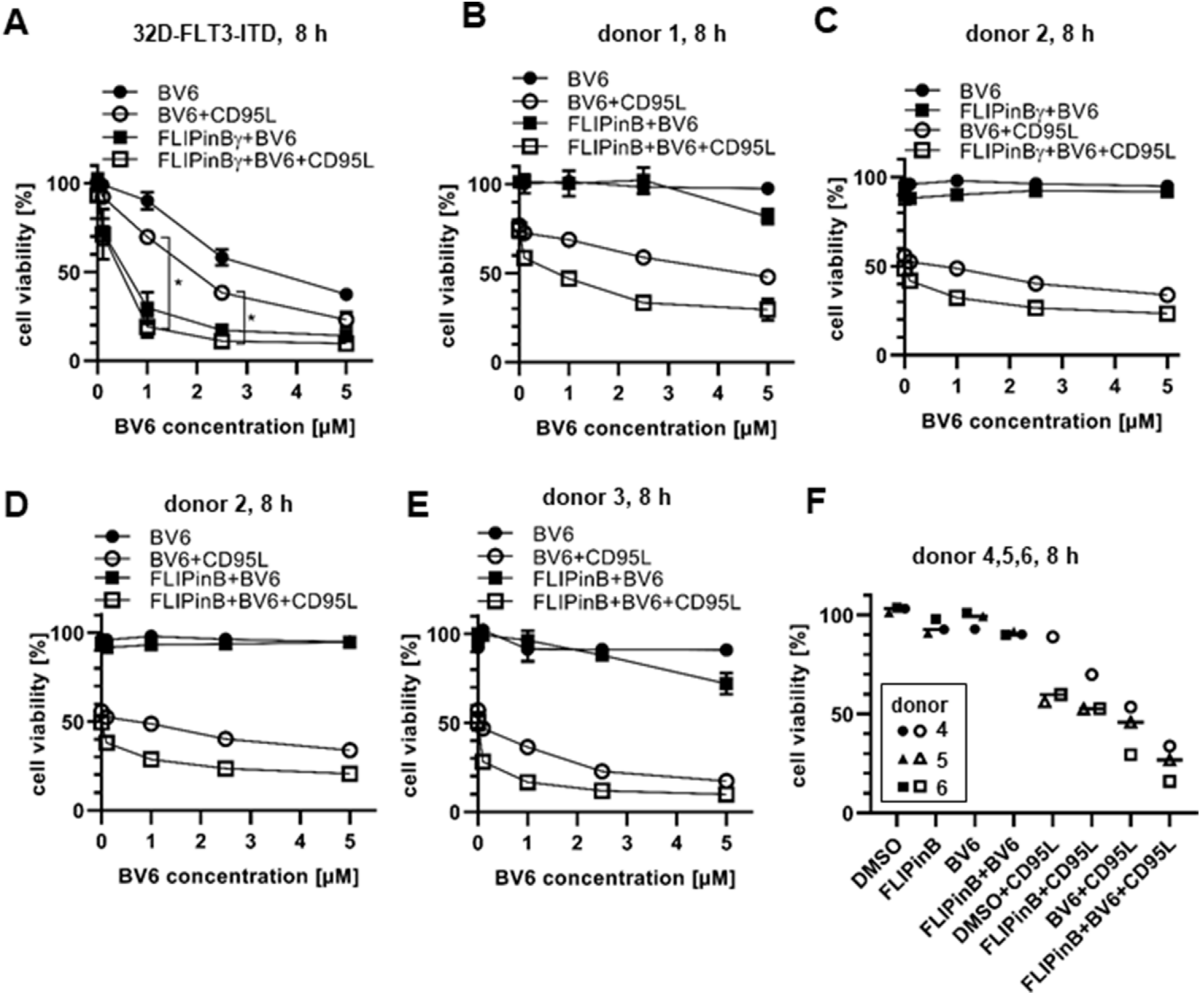
CD95L/BV6/FLIPin co-treatment induces the loss of cell viability in AML cell lines and primary T cells **(A)** 32D-FLT3-ITD cells were pretreated with 20 µM FLIPinBγ and BV6 (concentration range or 0.1 µM, 1 μM, 2.5 µM, 5 µM) for 2 h and then stimulated with 60 ng/mL CD95L (SFL) for 6 h **(B–E)** Primary T cells [donor 1 **(B)**, donor 2 **(C, D)**, donor 3 **(E)**] were pretreated with 20 µM FLIPinB **(B, D, E)** or 20 µM FLIPinBγ **(C)** for 2 h and BV6 (concentration range 0.1 µM, 1 μM, 2. 5 μM, 5 µM) and then stimulated with 500 ng/mL CD95L (SFL) for 6 h. **(F)** Primary T cells from donors 4, 5, 6 were pretreated with 20 µM FLIPinB for 2 h and with 5 µM BV6 for 1 h. The donors were then treated with 500 ng/mL CD95L (SFL) for 6 h. Cell viability was measured using the Cell Titer-Glo^®^ Luminescent Cell Viability Assay. The mean and standard deviation of three independent experiments **(A, F)** or one representative experiment **(B–E)** are shown. For statistical analysis, values were calculated using unpaired one-way ANOVA test with Tukey *post hoc* test. The following values were used: ****p < 0.0001,***p < 0.001; **p < 0.01; *p < 0.05; ns not significant.

It was shown previously that CD95L/BV6 treatment sensitizes acute myeloid leukemia (AML) cells to CD95L-induced cell death (Hillert et al., 2019). Specifically, these effects were reported in 32D-FLT3-ITD cells harbouring the FLT3-ITD mutation (Hillert et al., 2019). To analyze whether the treatment with FLIPin compounds can enhance the effects of BV6 and CD95L/BV6 stimulation, 32D-FLT3-ITD cells were treated with BV6/FLIPin or CD95L/BV6/FLIPin (Figure 2A). Dose-dependent BV6 treatment resulted in a loss of cell viability, which was enhanced by co-stimulation with CD95L. This was in agreement with previous reports (Hillert et al., 2019). Remarkably, FLIPin compounds enhanced both BV6 and CD95L/BV6-induced loss of cell viability (Figure 2A). This decrease was similar upon the high concentrations of BV6 and stronger for CD95L/BV6 stimulation at lower concentrations of BV6 (Figure 2A). A similar tendency was observed in other AML cell line MV4-11. In these cells, FLIPins enhanced the loss of cell viability induced by BV6 or TRAIL/BV6 stimulation (Supplementary Figure S1E).

Importantly, AML cells were much more sensitive towards BV6 and BV6/FLIPin treatments compared to pancreatic and colorectal cancer cell lines. Hence, after analysing the effects of FLIPin compounds in combination with BV6 and DLs on AML cells, we next tested the effects of these combinations on primary T cells from human healthy donors. The non-activated primary T cells (T cells, day 0) were resistant to CD95L or TRAIL treatments with or without co-treatment with FLIPins (Supplementary Figures S2A–SD). This resistance was fully in accordance with previous reports (Schmitz et al., 2003). Activated primary T cells, treated with IL-2 for 6 days (day 6 primary T cells (Schmitz et al., 2003)), have shown sensitivity towards CD95L treatment, which was also consistent with previous reports (Schmitz et al., 2003). Importantly, they were less sensitive to BV6 treatment compared to 32D-FLT3-ITD cells, but still lost their viability to small extent upon addition of FLIPin. Furthermore, they were slightly sensitised by FLIPins upon CD95L/BV6 treatment (Figures 2B–F). These results indicate that FLIPins can sensitise AML cells to BV6 and DL/BV6 treatments, but day 6 primary T cells only to DL/BV6 treatment, albeit to a lesser extent. These shows that primary T cells are more resistant towards combinatorial treatments CD95L/BV6/FLIPin and especially BV6/FLIPin.

### FLIPin compounds enhance CD95L/BV6-induced caspase activity

**FIGURE 3:**
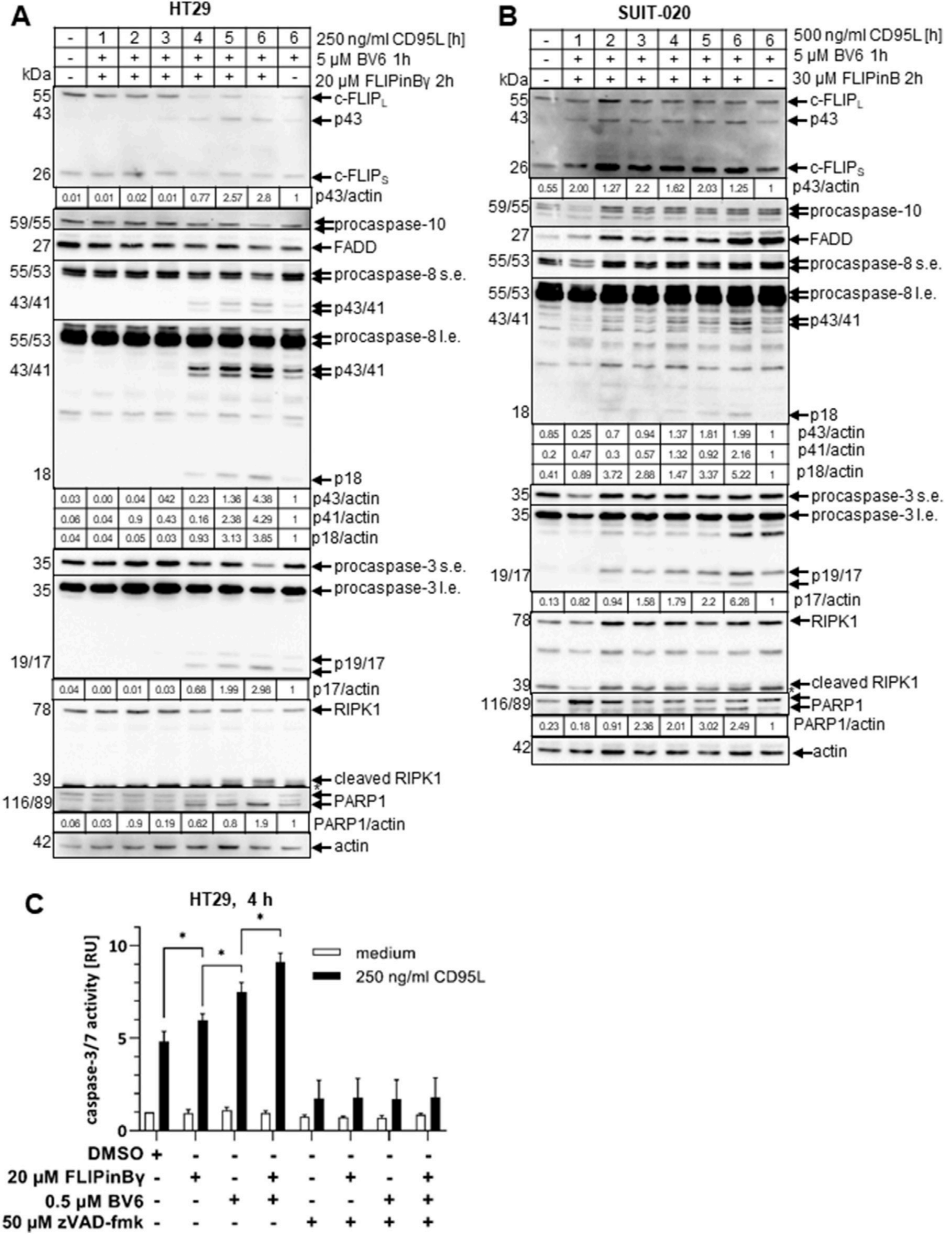
CD95L/BV6/FLIPin co-treatment leads to more caspase activity than CD95L/BV6 co-treatment **(A, B)** HT29 cells **(A)** or SUIT-020 cells **(B)** were pretreated for 2 h with 20 µM FLIPinBγ **(A)** or 30 µM FLIPinB and for one 1 h with 5 µM BV6 **(B)**. 250 ng/mL **(A)** or 500 ng/mL **(B)** CD95L was added for the indicated timepoints. Western Blot was analysed using the corresponding antibodies. Actin served as loading control. One representative Western Blot out of three is shown. Quantification values are shown under the corresponding Western Blot. Quantification values were normalized to the corresponding actin band. Quantification values from n = 3 are shown in Supplementary Figure S2A **(B) (C)** HT29 cells were 2 h prestimulated with 20 µM FLIPinBγ and 1 h 5 μM BV6, 50 µM zVAD-fmk. Subsequently 250 ng/mL CD95L for 4 h was added. Caspase-3/7 activity was measured using the Caspase-Glo^®^3/7 Assay. Mean and standard deviation from three independent experiments are shown. For statistical analysis values were calculated with unpaired One-way ANOVA test with Tukey *post hoc* test. Following values were used: ****p < 0.0001,***p < 0.001; **p < 0,01; *p < 0,05; ns not significant. Abbreviations: s.e. short exposure, l.e. long exposure.

Both BV6 and DL/BV6 treatments lead to RIPoptosome formation, which in turn triggers apoptosis in the sensitive cells (Feoktistova et al., 2011). To test whether FLIPins enhance the loss of cell viability via the apoptotic branch of the cell death pathway, Western Blot analysis of HT29 and SUIT-020 cells treated with CD95L/BV6 versus CD95L/BV6/FLIPin was performed (Figures 3A, B). This analysis revealed the faster accumulation of caspase cleavage products: caspase-8-p43/p41, caspase-8-p18 and caspase-3-p19/p17 upon CD95L/BV6/FLIPin treatment compared to CD95L/BV6 treatment in both HT29 and SUIT-020 cells (Figures 3A, B; Supplementary Figures S2E, SF). In particular, the stronger processing of procaspase-3 and higher levels of catalytically active caspase-3 p17 subunit were observed for both cell lines at the 6 h stimulation time point, indicating higher rate of apoptosis upon addition of FLIPins to the cells (Figures 3A, B; Supplementary Figures S2E, SF). Furthermore, the faster cleavage of PARP1, which is a substrate of caspase-3, was observed in HT29 and SUIT-020 cells upon CD95L/BV6/FLIPin compared to CD95L/BV6 stimulation indicating an increased rate of execution phase of apoptosis upon administration of FLIPins (Figures 3A, B).

Analysis of caspase-3/7 activity in HT29 cells upon CD95L/BV6 versus CD95L/BV6/FLIPin treatments demonstrated an increase in caspase-3/7 activity upon addition of FLIPins to the cells (Figure 3C). This was consistent with the results of the Western Blots analysis, which also have shown the higher rate of caspase-3 processing and PARP1 cleavage upon administration of FLIPins. Taken together, these results demonstrate that FLIPin enhances caspase activity and apoptosis induced by CD95L/BV6 treatment.

### FLIPin compounds enhance phosphorylation of MLKL and RIPK1 upon DL/BV6/zVAD-fmk treatment

**FIGURE 4:**
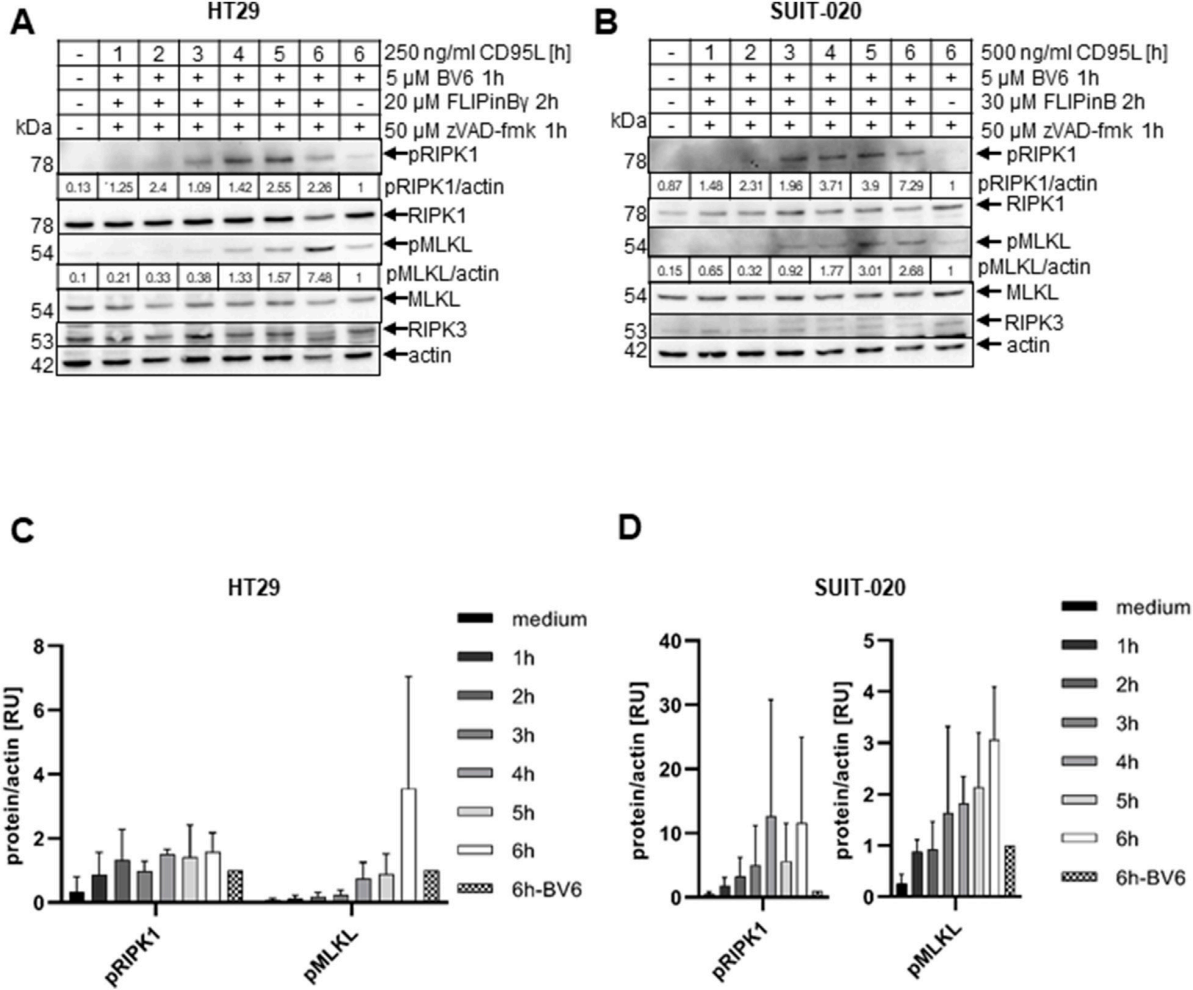
CD95L/BV6/FLIPin co-treatment leads to stronger activation of necroptotic markers than BV6/CD95L co-treatment **(A, B)** HT29 cells **(A)** or SUIT-020 cells **(B)** were pretreated for 2 h with 20 µM FLIPinBγ **(A)** or 30 µM FLIPinB **(B)** and for 1 h with 5 µM BV6 and 50 µM zVAD-fmk. 500 ng/mL **(B)** or 250 ng/mL **(A)** CD95L was added for the indicated time points. Western Blot was analysed using the corresponding antibodies. Actin served as loading control. One representative Western Blot out of three is shown. Quantification values are shown under the corresponding Western Blot. Quantification values were normalized to the corresponding actin band. **(C, D)** Quantifications from n = 3 from Western Blots **(A, B)** in HT29 cells **(C)** and SUIT-020 cells **(D)** are shown.

Both BV6 and DL/BV6 treatments can also induce necroptosis upon inhibition of caspases in RIPK3-containing cells (Feoktistova et al., 2011; Pietkiewicz et al., 2015a). HT29 and SUIT-020 cells contain RIPK3 (Supplementary Figure S2G). Subsequently, next we investigated the influence of FLIPins on the necroptosis pathway induced by co-stimulation with DL, BV6 and the pan-caspase inhibitor zVAD-fmk in HT29 and SUIT-020 cells. Western Blot analysis of HT29 and SUIT-020 cells treated with CD95L/BV6/zVAD-fmk versus CD95L/BV6/FLIPin/zVAD-fmk revealed the stronger phosphorylation of RIPK1 (pRIPK1) and MLKL (pMLKL), upon FLIPin treatment after 6 h of stimulation with CD95L/BV6/zVAD-fmk (Figures 4A, B). pRIPK1 and pMLKL are well-established necroptosis markers indicating the activation of the necrosome or complex IIb and subsequent necroptosis induction. Their faster accumulation was detected in both HT29 and SUIT-020 cells, indicating a more rapid activation of necroptosis upon CD95L/BV6/FLIPin/zVAD-fmk treatment (Figures 4A–D).

### FLIPin compounds enhance DL/BV6-mediated cell death

**FIGURE 5:**
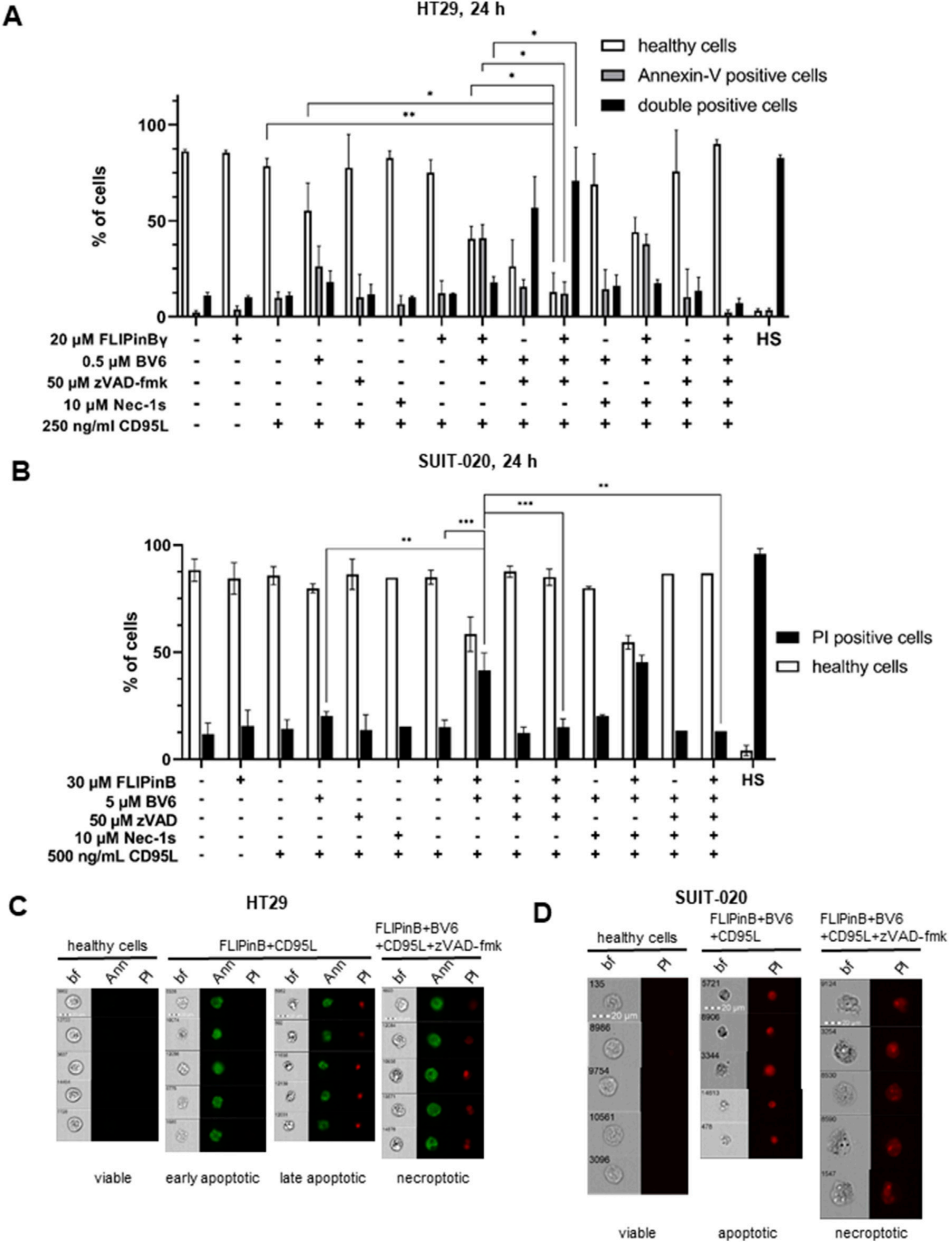
Co-treatment with CD95L/BV6/FLIPin induces both apoptosis and necroptosis **(A)** HT29 cells were pretreated with 20 µM FLIPinBγ for 2 h and with 0.5 µM BV6 and 50 µM zVAD-fmk and 10 µM Nec-1s for 1 h. The cells were then treated with 250 ng/mL CD95L for 22 h. Cells were stained with Annexin-V-FITC and PI. Populations were gated for Annexin-V-FITC negative (healthy or viable), Annexin-V-FITC positive (early apoptotic) and double positive (late apoptotic) cells. **(B)** SUIT-020 cells were pretreated with 30 µM FLIPinB for 2 h and with 5 μM BV6 and 50 µM zVAD-fmk and 10 µM Nec-1s for 1 h. The cells were then treated with 500 ng/mL CD95L for 22 h. Cells were stained with PI only. Cell death was measured by imaging flow cytometry. Populations were gated for negative (healthy or viable) and PI positive cells. Mean and standard deviation of three independent experiments are shown. **(C, D)** Representative images are shown for HT29 cells **(C)** or SUIT-020 cells **(D)**. Images are shown for viable cells, early apoptotic (Annexin-V-FITC positive), late apoptotic and necroptotic (Annexin and PI double positive) cells **(C)**. Images are shown for viable and PI-positive cells **(D)**. For statistical analysis, values were calculated using unpaired one-way ANOVA test with Tukey *post hoc* test. The following values were used: ****p < 0.0001,***p < 0.001; **p < 0.01; *p < 0.05; ns not significant. Abbreviations: BF brightfield, PI propidium iodide.

Next we investigated, whether FLIPins enhance the DL/BV6-induced cell death induction. Specifically, we used an imaging flow cytometry approach that we developed to distinguish between apoptotic and necroptotic cell death (Pietkiewicz et al., 2015a). This assay is based on the combination of Flow Cytometry analysis via Annexin-V FITC and propidium iodide (PI) staining and bright field imaging of the morphology of dying cells. This combination allows the identification of key features of dying cells and their classification as apoptotic or necroptotic. Consistent with our observations using cell viability assays, this analysis showed that FLIPins enhance CD95L/BV6-induced cell death in SUIT-020 and HT29 cells (Figures 5A, B). Analysis of images of dying cells showed that CD95L/BV6/FLIPin-stimulated SUIT-020 and HT29 cells exhibited apoptotic morphology with the typical shrinkage of cells and formation of apoptotic blebs (Figures 5C, D). The significant increase in the amount of apoptotic cells upon FLIPin administration to CD95L/BV6-treated SUIT-020 cells was observed (Figure 5B). The same effects were detected in HT29 cells, as well as in MV4-11 and primary T cells, but the increase in apoptotic cells was less pronounced (Figure 5A; Supplementary Figure S3). Since FLIPin treatment also enhanced CD95L/BV6/zVAD-fmk-induced phosphorylation of RIPK1 and MLKL, the effects of FLIPins on CD95L/BV6/zVAD-fmk-induced necroptosis were also analysed in SUIT-020 and HT29 cells. Co-treatment with zVAD-fmk resulted in the appearance of cells with necroptotic morphology characterized by an increase in cell volume (Figures 5C, D). CD95L/BV6/zVAD-fmk-induced cell death was partially blocked by Nec-1s administration, indicating the induction of necroptosis (Figures 5A, B). In turn, FLIPins caused an enhancement of DL/BV6/zVAD-fmk-induced necroptosis in SUIT-020 and HT29 cells, but this enhancement was not strong (Figures 5A, B). These results further confirmed that FLIPin compounds prominently enhance DL/BV6-mediated apoptotic cell death with small effects on the enhancement of DL/BV6/zVAD-fmk-induced necroptosis.

### FLIPin compounds enhance complex II assembly

**FIGURE 6:**
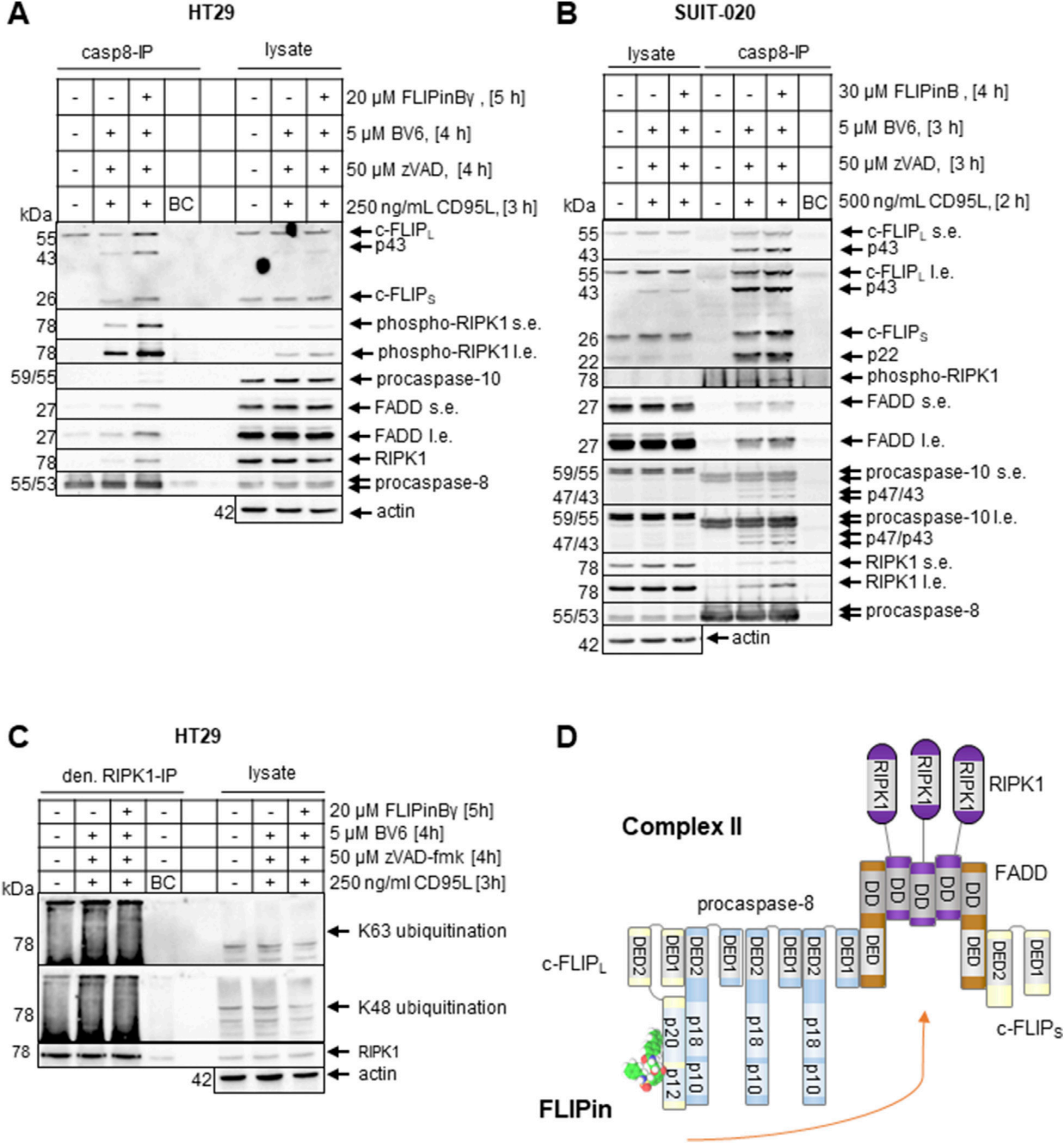
FLIPin compounds enhance complex II assembly **(A, B)** HT29 cells **(A)** or SUIT-020 cells **(B)** were pretreated for 2 h with 20 µM FLIPinBγ **(A)** or 30 µM FLIPinB **(B)** and for 1 h with 5 µM BV6 and 50 µM zVAD-fmk. Afterwards cells were treated with 250 CD95L ng/mL for 3 h **(A)** or 500 ng/mL CD95L for 2 h **(B)**. Complexes were immunoprecipitated using anti-caspase-8 antibodies. Caspase-8 was used as a loading control for IP and actin was used as loading control for lysate. **(C)** HT29 cells were pretreated for 2 h with 20 µM FLIPinBγ and for 1 h with 5 µM BV6 and 50 µM zVAD-fmk. Afterwards cells were treated with 250 CD95L ng/mL for 3 h. Lysates were shaked for 5 min at 95°C with addition of 10% SDS to a final concentration of 1%. RIPK1 was immunoprecipitated using anti-RIPK1 antibody. One representative Western Blot out of three **(A, B)** or two **(C)** is shown. **(D)** Scheme of complex II assembly and putative effects of FLIPin. Abbreviations: IP: Immunoprecipitation, s.e. short exposure, l.e. long exposure.

CD95L/BV6 treatment leads to formation of complex II (Geserick et al., 2009). To test how FLIPin compounds affect complex II assembly, we performed immunoprecipitation (IP) using anti-caspase-8 antibodies (casp8-IP) from SUIT-020 and HT29 cells (Figures 6A, B). HT29 and SUIT-020 cells were stimulated with CD95L/BV6 in the presence of zVAD-fmk. The casp8-IPs showed efficient pull-down of core complex II components together with the phosphorylated form of RIPK1 (pRIPK1). The latter could serve as an indicator for the activation of this kinase in complex II, in agreement with previous reports (Liu et al., 2018). Importantly, an increase in the amount of complex II was detected upon co-treatment with FLIPins. In particular, this was observed for the core components of complex II such as FADD, RIPK1 and c-FLIP in casp8-IPs from both cell lines, with stronger effects in HT29 cells.

It has been reported that the stability of complex II is controlled by multiple ubiquitination linkages of RIPK1 signalling complexes (Peltzer et al., 2016; Kist et al., 2021), suggesting that FLIPin may influence the ubiquitination status of RIPK1 and complex II. To test this hypothesis, denaturing IPs of RIPK1 (den. RIPK1-IP) were performed with the addition of SDS (Figure 6C). A very small increase in the degree of K63 ubiquitination was detected in the denaturing IPs, which may also contribute to the stabilisation of complex II. However, no increase in ubiquitination of complex II was detected in conventional, non-denaturating IPs of complex II using anti-pRIPK1 antibodies (Supplementary Figure S4). Thus, no strong influence of ubiquitination events on the stability of complex II was detected. This suggests that targeting the caspase-8/c-FLIP_L_ heterodimer with FLIPins leads to stabilization of complex II and an increase in its level, however this is not directly linked to ubiquitination (Figure 6D).

### FLIPin compounds enhance CD95L/BV6-mediated cell viability loss of AML cells from primary patients

**FIGURE 7:**
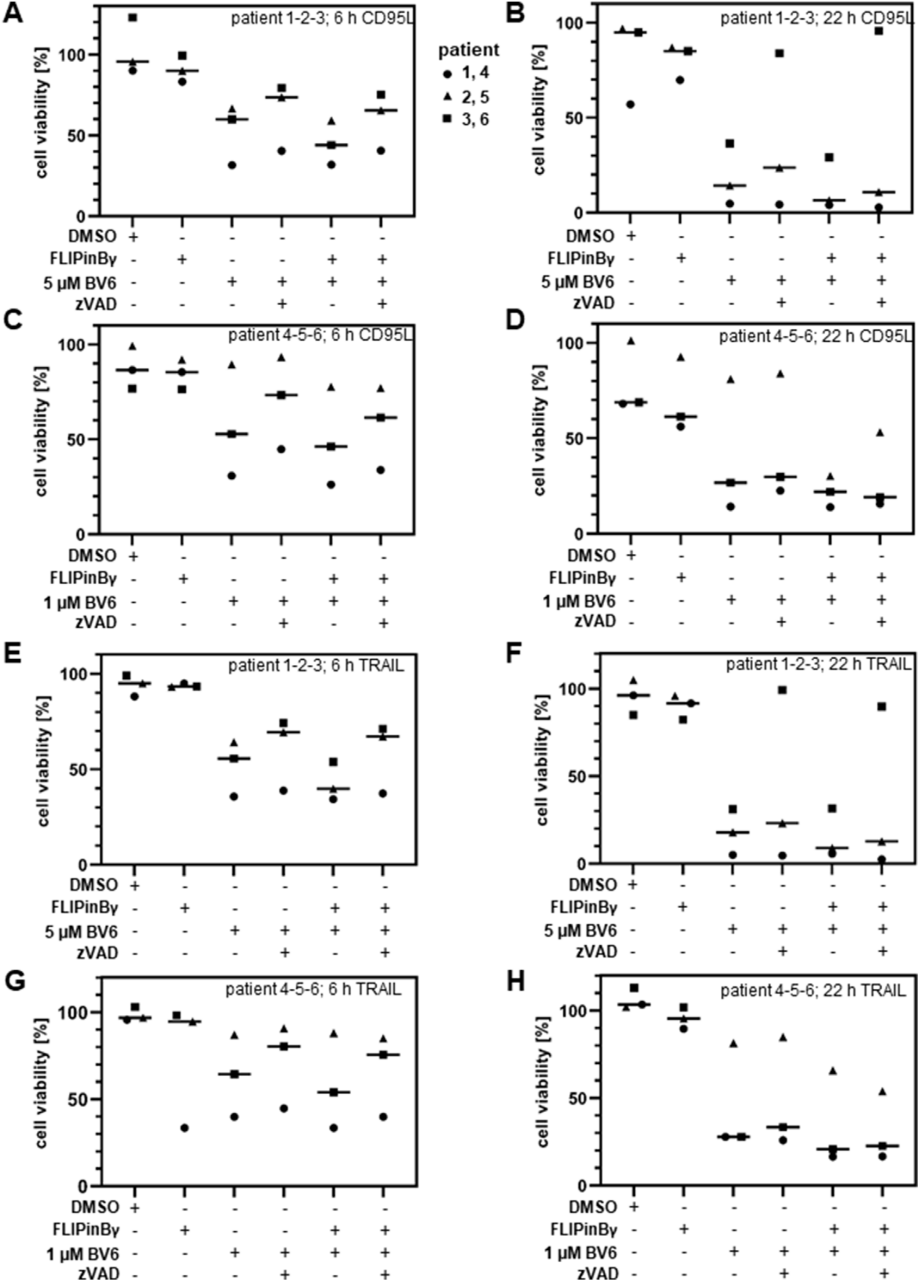
CD95L/BV6/FLIPin co-treatment enhances cell viability loss in primary AML cells **(A–H)** Primary AML patient samples were preteated with 20 µM FLIPinBγ for 2 h and with 1 µM BV6 **(C, D and G, H)** or 5 µM BV6 **(A, B, E, F)** and with 50 µM zVAD-fmk, 10 µM Nec-1s for 1 h. Afterwards cells were stimulated with 60 ng/mL CD95L (SFL) **(A, B, C, D)** or 100 ng/mL TRAIL **(E, F, G, H)** for 6 h **(A, C, E, G)** or 22 h **(B, D, F, H)**. Cell viability was measured using the Cell Titer-Glo^®^-Luminescent Cell Viability Assay. The mean and standard deviation of three independent experiments are shown.

**FIGURE 8:**
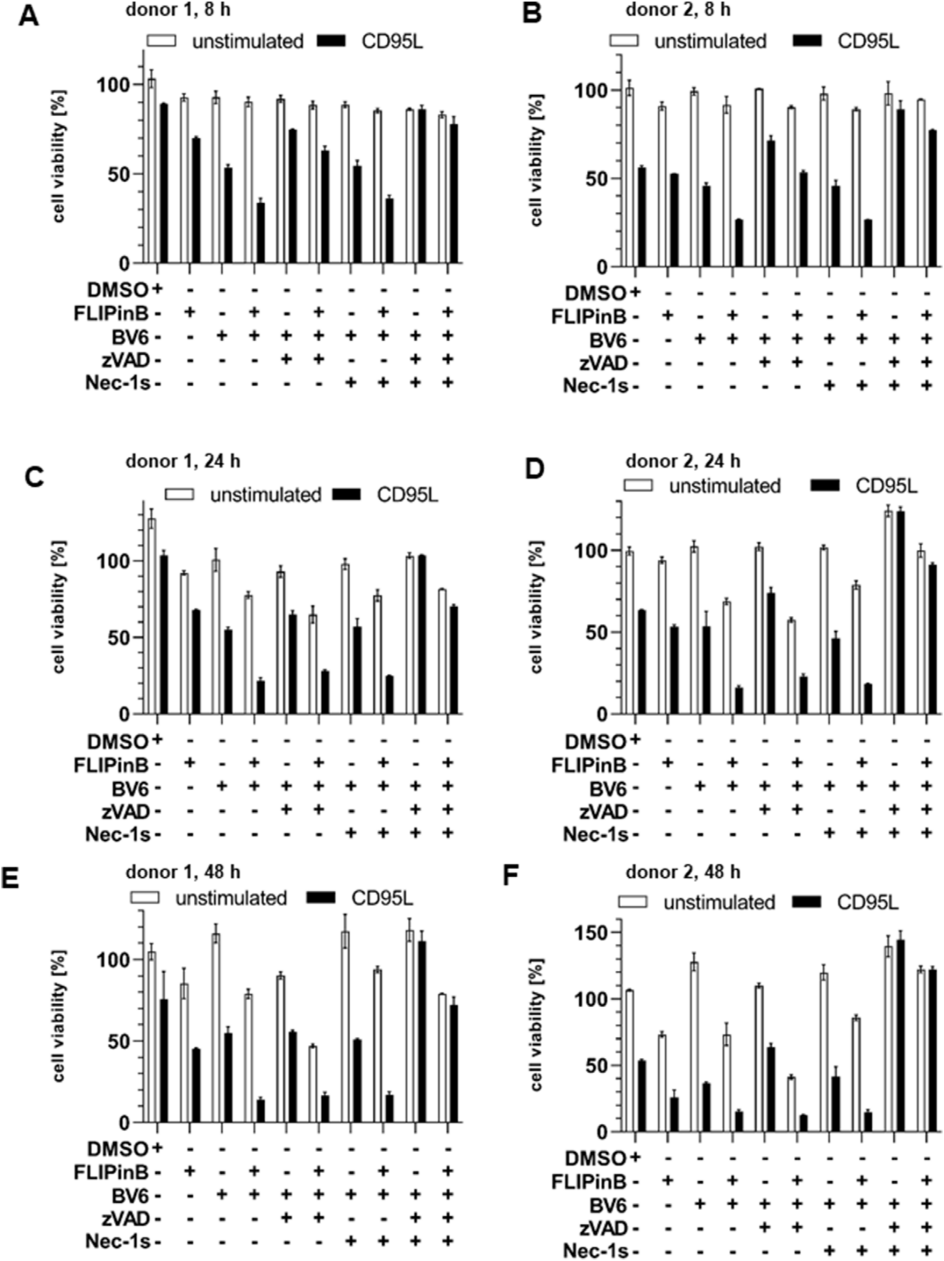
CD95L/BV6/FLIPin co-treatment enhances cell viability loss in human T cells **(A–F)** Human peripheral blood T cells from different donors were pretreated with 20 µM FLIPinB for 2 h and pretreated for 1 h with 5 μM BV6, 50 µM zVAD-fmk and 10 µM Nec-1s. Subsequently cells were stimulated with 100 ng/mL CD95L for 22 h. Cell viability was measured using the Cell Titer-Glo^®^-Luminescent Cell Viability Assay. The mean and standard deviation from one representative experiment are shown.

Since an increase in the cell death upon DL/BV6/FLIPin treatment was observed in the cancer cell lines, the effects of this co-treatment were also tested on the material from the primary cancer patients. In particular, cells isolated from AML patients were treated with DL/BV6 in combination with FLIPins as well as the caspase inhibitor zVAD-fmk or the necroptosis inhibitor Nec-1s (Figure 7). Consistent with our observations in AML cancer cells, this analysis showed that FLIPins enhanced the CD95L/BV6-induced the loss of cell viability in the samples from AML patients. Furthermore, FLIPins also slightly increased the loss of cell viability upon co-treatment with zVAD-fmk, namely upon co-treatment with CD95L/BV6/zVAD-fmk/FLIPin versus CD95L/BV6/zVAD-fmk. To test the effects of CD95L/BV6/FLIPin versus CD95L/BV6 co-treatment and CD95L/BV6/zVAD-fmk/FLIPin versus CD95L/BV6/zVAD-fmk co-treatment on non-cancer cells, we used day 6 activated primary T cells from healthy donors (Figure 8; Supplementary Figure S5). The day 6 primary cells were sensitive only to CD95L/BV6 and CD95L/BV6/FLIPin-mediated treatments. This was in line from the data of the Figure 2. Hence, CD95L stimulation can make both AML cells and non-tumor cells sensitive to BV6/FLIPin, with AML cells being much more sensitive to FLIPin/BV6 without CD95 stimulation than primary T cells.

## Discussion

Targeting the DED proteins c-FLIP and caspase-8 is attracting increasing attention due to their key role in controlling cell fate (Ivanisenko et al., 2022). The caspase-8/c-FLIP_L_ heterodimer is one of the key targets due to its important role in both apoptotic, necroptotic and other cell death pathways (Hughes et al., 2016; Tummers and Green, 2017; Pop et al., 2011; Muendlein et al., 2020; Newton et al., 2019; Davidovich et al., 2023; Liccardi et al., 2019). In previous studies, we developed a family of chemical compounds, FLIPins, that specifically target the caspase-8/c-FLIP_L_ heterodimer and tested their effects on DL stimulation. Here, to further explore the effects of FLIPin compounds, both of FLIPinB and its water-soluble version FLIPinBγ, we tested their effects on co-stimulation with DL and the SMAC mimetic BV6. FLIPins were found to be efficient in enhancing cell death upon co-stimulation with DL/BV6 in several cancer cells, which was accompanied by an increase in complex II formation.

**FIGURE 9:**
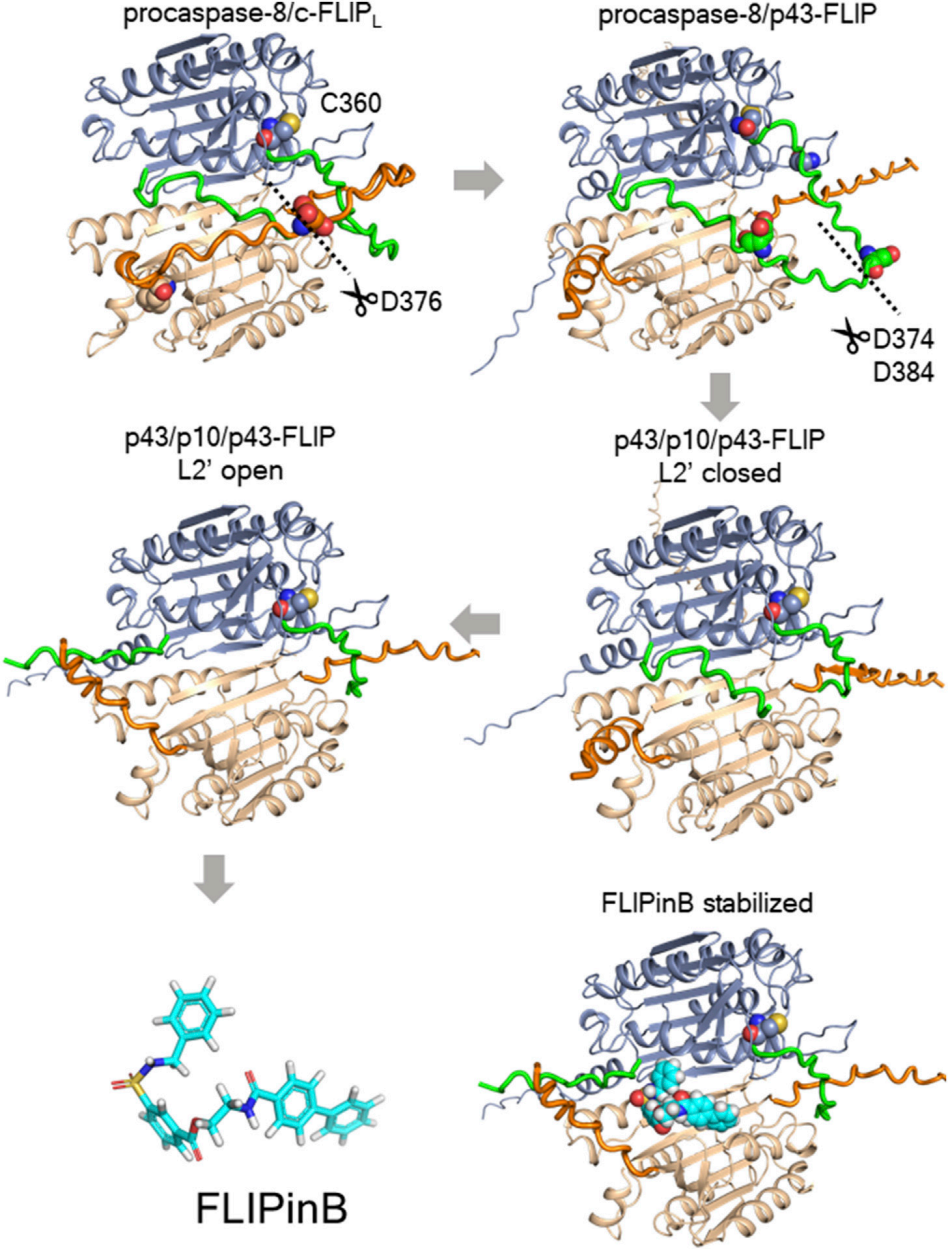
The potential mechanism of stabilization effects by FLIPin compounds. Molecular models of procaspase-8/c-FLIP_L_ heterodimer processing and stabilization by FLIPinB as predicted using AlphaFold2 and AlphaFold3 models. The p43/p10/p43-FLIP model was predicted using the AlphaFold2-multimer model via the ColabFold implementation. The models for procaspase-8/c-FLIP_L_, procaspase-8/p43-FLIP, and p43/p10/p43-FLIP were predicted using AlphaFold3. The structural model of FLIPinB bound to p43/p10/p43-FLIP was based on our previous model (Ivanisenko and Lavrik, 2020). In the visualizations, procaspase-8 regions are shown in blue, the L2 loop of procaspase-8 in green, c-FLIP_L_ in brown, the L2 loop of c-FLIP_L_ in orange, and FLIPinB in cyan.

An increase in complex II formation is fully consistent with recent findings using uncleavable c-FLIP_L_ (Martinez Lagunas et al., 2023). Specifically, the study by Lagunas et al. examined the effects of c-FLIP_L_ cleavage leading to the generation of p43-FLIP by generating an uncleavable variant of murine c-FLIP_L_ and testing its effects on the TNFα pathway in *in vivo* models (Martinez Lagunas et al., 2023). Mechanistically, uncleavable c-FLIP_L_ was shown to enhance complex II assembly upon TNFα stimulation and likely serves as an important scaffold for the assembly of this complex. Therefore, it is possible that FLIPins, which were designed to replace the cleaved portion of caspase-8 in the caspase-8/c-FLIP_L_ heterodimer, also increase the stability of the heterodimer, which in turn leads to the increased stability of complex II (Figure 9). Furthermore, the FLIPin compounds binding site is located in close proximity to the L2 loop comprising the aspartate residue of c-FLIP_L_, which leads to the generation of the p43-FLIP cleavage product (Figure 9). Thus, this region may be critical for the stability of the caspase-8/c-FLIP_L_ heterodimer, which in turn may serve as an important scaffold for complex II assembly. The molecular mechanism of the increase in complex II formation upon addition of FLIPin compounds remains to be elucidated in future studies.

The other possibility for these stabilizing effects of FLIPins could be due to possible interference with post-translational modifications (PTMs) of the C-terminal portions of procaspase-8 and c-FLIP_L_. The PTMs of the C-terminal region of these proteins such as phosphorylation, ubiquitination, methylation and others play a key role in the control of apoptosis induction (Ivanisenko et al., 2022; Wohlfromm et al., 2024; Seyrek et al., 2020; Seyrek et al., 2024). The binding of FLIPins could alter the conformation of this region, leading to possible effects on the corresponding PTMs, which in turn would promote the accumulation of the heterodimer.

Importantly, we used the SMAC mimetic BV6 in these experiments, which was reported to induce the production of TNFα (Varfolomeev et al., 2007; Vince et al., 2007). This, in turn may contribute to the induction of cell death under the conditions used in the study. It is likely that FLIPin is able to stabilise the complexes resulting from both TNFα/BV6 and CD95L/TRAIL/BV6 stimulation, as its action is suggested to be primed only by the presence of the heterodimer caspase-8/c-FLIP_L_. However, the detailed role of FLIPin on TNFα pathway activity and complex II assembly needs to be analysed in future studies, as it has not yet been addressed.

In this study, we also examined the potential of using DL/BV6/FLIPin in the treatment of cancer cells. With a number of recently developed SMAC mimetics being intensively studied in clinical trials, this direction is of major relevance (Morrish et al., 2020). In particular, we focused on AML cells, since targeting c-FLIP is suggested to be of paramount importance in these cells (McLornan et al., 2013; Humphreys et al., 2018). The effects of DL/BV6/FLIPin co-treatment resulted in a greater loss of cell viability of AML cancer cells compared to DL/BV6 co-treatment. The same difference was monitored in the experiments using the primary material from AML patients. However, a loss of cell viability of primary activated T cells was also observed with DL/BV6 treatment. Importantly, however, BV6 alone and the BV6/FLIPin combination did not show strong effects on primary T cells, suggesting the possibility for new therapeutic options. Therefore, in future studies, the detailed analysis of the primary T cells treated with lower doses of these stimulating agents should be considered in order to develop more promising therapeutic strategies based on FLIPin combinations. In addition, we also observed sensitization effects of FLIPins on pancreatic and colon cancer cells, while almost no effects were observed on human primary fibroblasts, which served as a model for normal, non-cancerous cells. Therefore, this type of cancer cells may also be considered for combination therapies with FLIPins in future studies.

Targeting the caspase-8/c-FLIP_L_ heterodimer is important for the development of novel therapeutic approaches as well as for gaining new insights into molecular mechanisms. In this study, we found that targeting this heterodimer via FLIPins enhances complex II assembly in the CD95L/BV6-mediated signaling pathway. The analysis of the role of the heterodimer as a scaffold is very important for future studies addressing the function of DED-containing complexes in apoptosis, necroptosis, inflammation and pyroptosis and may provide innovative directions for therapeutic approaches.

## Methods

### Cell lines

The pancreatic cancer cell line SUIT-020 was cultured in DMEM/Ham’s F-12 (Merck KGaA, Germany) supplemented with 10% heat-inactivated fetal calf serum (FCS) and 1% Penicillin-Streptomycin in 5% CO_2_. The colon cancer cell line HT29 was cultured in DMEM (Thermo Fisher Scientific, United States) supplemented with 10% heat-inactivated fetal calf serum (FCS) and 1% Penicillin-Streptomycin in 5% CO_2_. Primary human fibroblasts (#1451) and Murine hematopoietic 32D cells stably expressing human FLT3-ITD 598/599 were cultured in RPMI 1640 (Thermo Fisher Scientific, United States) supplemented with 10% heat-inactivated fetal calf serum (FCS) and 1% Penicillin-Streptomycin in 5% CO_2_. AML cell line MV4-11 was cultured in RPMI 1640 (Thermo Fisher Scientific, United States) supplemented with 10% heat-inactivated FCS and 0.02% Plasmocin and 5.8% Additivum (β-Mercaptoethanol, 1M HEPES buffer pH 7.2, 100 mM Sodium pyruvate, 200 mM L-Glutamine, 10x non-essential amino acids, 10 mg/ml L-Aspartic acid).

### Primary cell culture

Primary human T cells were obtained from anonymous healthy donors whose age and sex are unknown and not relevant to the experiments. Briefly, human peripheral blood mononuclear cells were purified by Ficoll gradient centrifugation (Biochrom, Germany) of heparinized blood and subsequently primary T cells by non-T cell depletion using the “Pan T cell isolation Kit II” (Miltenyi Biotec, Germany) (Arndt et al., 2013). T cells were cultured in RPMI 1640 medium (Thermo Fisher Scientific, United States) supplemented with 10% heat-inactivated fetal calf serum (FCS) and 1% Penicillin-Streptomycin in 5% CO_2_ and 1 μg/mL phytohemagglutinin (PHA, Remel^™^; #R30852801). After 24 h 25 U/mL IL-2 (Millipore Sigma; #11011456001) was added for 6 days. This study was approved by the Ethics Committee of the University Hospital Magdeburg (141/19).

Cells from AML patients were obtained from bone marrow of patients (Table 1) (Hillert et al., 2019). Cells were isolated from heparinized blood and mononuclear cells (MNC) were purified by Histopaque densitiy gradient centrifugation. MNCs were cultured in RPMI 1640 (Thermo Fisher Scientific, United States) supplemented with 10% heat-inactivated FCS in 5% CO_2_. AML blasts were obtained from patient’s bone marrow following informed consent. The studies were approved by the institutional review board for Medical Faculty of Otto-von-Guericke-University, Magdeburg, Germany (115/08).

**TABLE 1:** AML patients.

Patient number	FLT3-ITD status	NPM1 status	Percentage of blasts in bone marrow (%)
1	positive	Mutated	5
2	positive	Wild type	69
3	positive	n.d	65
4	positive	Mutated	85
5	positive	n.d	99
6	positive	n.d	43

### Antibodies and reagents

The following antibodies were used for Western Blot analysis: monoclonal phospho-RIPK1 (Ser166) (#65746), monoclonal anti-RIPK1 XP (#3493), polyclonal anti-caspase-3 (#9662), polyclonal anti-PARP1 (#9542) and monoclonal anti-MLKL (#14493) antibodies from Cell Signaling Technology, United States; polyclonal anti-actin (A2103) antibodes from Sigma-Aldrich, Germany; anti-pMLKL (ab187091) antibodies from Abcam, Great Britain; monoclonal anti-caspase-10 (M059-3) antibody from MBL international, United States; monoclonal anti-FADD (clone 1C4), monoclonal anti-caspase-8 (clone C15), and monoclonal anti-c-FLIP (clone NF6) antibodies were a kind gift of Prof. P.H. Krammer, (DKFZ, Heidelberg). Horseradish peroxidase-conjugated goat anti-mouse IgG1, -2b, goat anti-rabbit and rabbit anti-goat antibodies were from Southern Biotech (United States). Monoclonal anti-caspase-8 (clone C15) was used for immunoprecipitations (IPs). All chemicals were of analytical grade and purchased from Merck (Germany) or Sigma (Germany). Recombinant LZ-CD95L was produced as described (Fricker et al., 2010). BV6 was provided by Genentech, Inc. (# OR-502922), the pan-caspase inhibitor zVAD-fmk (BAC-4026865.0025) was from Bachem Holding, Switzerland, FLIPinB (Amb1202053) and FLIPinBγ (Amb37832612) was from Ambinter (France) and SuperFasLigand (SFL) (ALX-522-020-C005) was from Enzo Life Science. The RIPK1 inhibitor 7-Cl-O-Nec-1 (Nec-1s; 5.04297.0001) was obtained from Merck, Germany.

FLIPinB was dissolved in DMSO, therefore DMSO was used as a control in the experiments involving FLIPinB. FLIPinBγ was dissolved in water. Most of the experiments were performed with FLIPinB, but for some experiments, especially for AML cells and AML material from patients, FLIPinBγ was used.

### Western blot analysis and immunoprecipitations

Western Blot analysis was done with 1 × 10^6^ SUIT-020 or HT29 cells. The Western Blot analysis of total cellular lysates was carried out in accordance with our previous reports (Schmidt et al., 2015). The analysis of Western Blot images was performed by Image Lab 5.1 Software (BioRad). Immunoprecipitations (IPs) from 8 × 10^6^ SUIT-020 or HT29 cells were carried out as previously reported (Pietkiewicz et al., 2015b). In particular, 2 µg of anti-caspase-8 antibody (clone C15), anti-RIPK1 antibody (#3493, Cell Signaling) or anti-pRIPK1 antibody (#65746 Cell Signaling) and Sepharose A beads (Th. Geyer GmbH, Germany) were added to the cells. Beads control was performed via the pulldown with Sepharose A beads only. All IPs were rotated at 4°C overnight, washed four times with PBS and prepared for Western Blot analysis. For denaturating RIPK1 IP (den. RIPK1 IP) we used the protocol developed by us before (Wohlfromm et al., 2024). In particular, 10% SDS was added to the lysates of HT29 cells to a final concentration of 1% and shaked for 5 min at 95°C. The denaturated lysates were divided into lysate control (10%) and IP (90%).

### Cell viability assay and caspase-3/7 activity assay

1.2 × 10^4^ SUIT-020 or HT29 cells were seeded in 96 well plates a day before the experiments. 2 × 10^4^ 32D-FLT3-ITD, MV4-11 cells and MNCs, primary T cells were seeded on the day of experiment. After described incubation times of indicated treatments, 50 µL of the CellTiter-Glo^®^ 2.0 (CellTiter-Glo^®^ 2.0 Assay, Promega, Germany) or Caspase-Glo^®^ 3/7 (Caspase-Glo^®^ 3/7 Assay, Promega, Germany) solution was added to each sample. Measurements were accomplished according to manufacturer’s instructions. The luminescence intensity was measured by a microplate reader Infinite M200pro (Tecan, Switzerland). The viability of untreated cells was normalized to 100% and the caspase-3/7 activity of untreated cells was normalized to 1 RU (relative unit). Every condition was performed in duplicate.

### Synergism calculation

The data from the Figure 1 were used to calculate if the combined treatment leads to synergistic effects. As FLIPinB is only acting in combination with a DL we calculated the IC50 values considering the combinations of two stimulations: (FLIPinB/CD95L) and (BV6). These IC50 values were used to calculate the synergism. Values below 1 were taken as synergistic effects. The combined treatment in SUIT-020 led to synergistic effects while in HT29 cells only treatments with 250 ng/mL CD95L/FLIPinB were synergistic.

The synergism was further calculated using the Loewe formula:

$$x=\frac{a}{A}+\frac{b}{B}$$

x: calculated value, x < 1 synergism, x = 1 additive effects, x > 1 antagonism.

a: dosis component a.
A: IC50 value component a.
b: dosis component b.
B: IC50 value component b.

The IC50 value was plotted in a diagram and the calculated x was included in this diagram. Everything below the IC50 linear graph was taken as synergism.

### Statistical analysis

Unpaired one-way-ANOVA test with Tukey *post hoc* test was done using Graphpad Prism 8 software. The following values were used: ****p < 0.0001; ***p < 0.001; **p < 0.01; *p < 0.05; ns not significant.

### Molecular modelling

The molecular models of procaspase-8/c-FLIP_L_, procaspase-8/p43-FLIP, and p43/p10/p43-FLIP in the L2′ loop closed state were computed using the AlphaFold3 web server (Abramson et al., 2024; Mirdita et al., 2022). The molecular model of p43/p10/p43-FLIP in the L2′open state was predicted using the AlphaFold2-Multimer model via the ColabFold (v1.5.5) server (Mirdita et al., 2022; Jumper et al., 2021). c-FLIP_L_ included the 239-480 amino acid regions (UniProt ID O15519), procaspase-8 included the 236-479 regions (UniProt ID Q14790). p43/p10 included the 236-374 and 385-479 regions (UniProt ID Q14790). p43-FLIP included the 239-376 and 377-480 regions (UniProt ID O15519). The structural model of FLIPinB bound to p43/p10/p43-FLIP was derived by structural superimposition of the FLIPinB bound model derived from (Hillert et al., 2020b) on the AlphaFold2-multimer-based prediction using PyMOL software (https://pymol.org/2/).

## Data Availability

The original contributions presented in the study are included in the article/Supplementary Material, further inquiries can be directed to the corresponding author.
