# Supplementary material for: Targeting caspase-8/c-FLIPL heterodimer in complex II promotes DL-mediated cell death
Source: Front Cell Dev Biol. 2024 Sep 30;12:1471216. doi: 10.3389/fcell.2024.1471216 (PMC11471875; doi:10.3389/fcell.2024.1471216)
Supplement: Supplementary file 1 [file Presentation1.PPTX]

## Slide 1
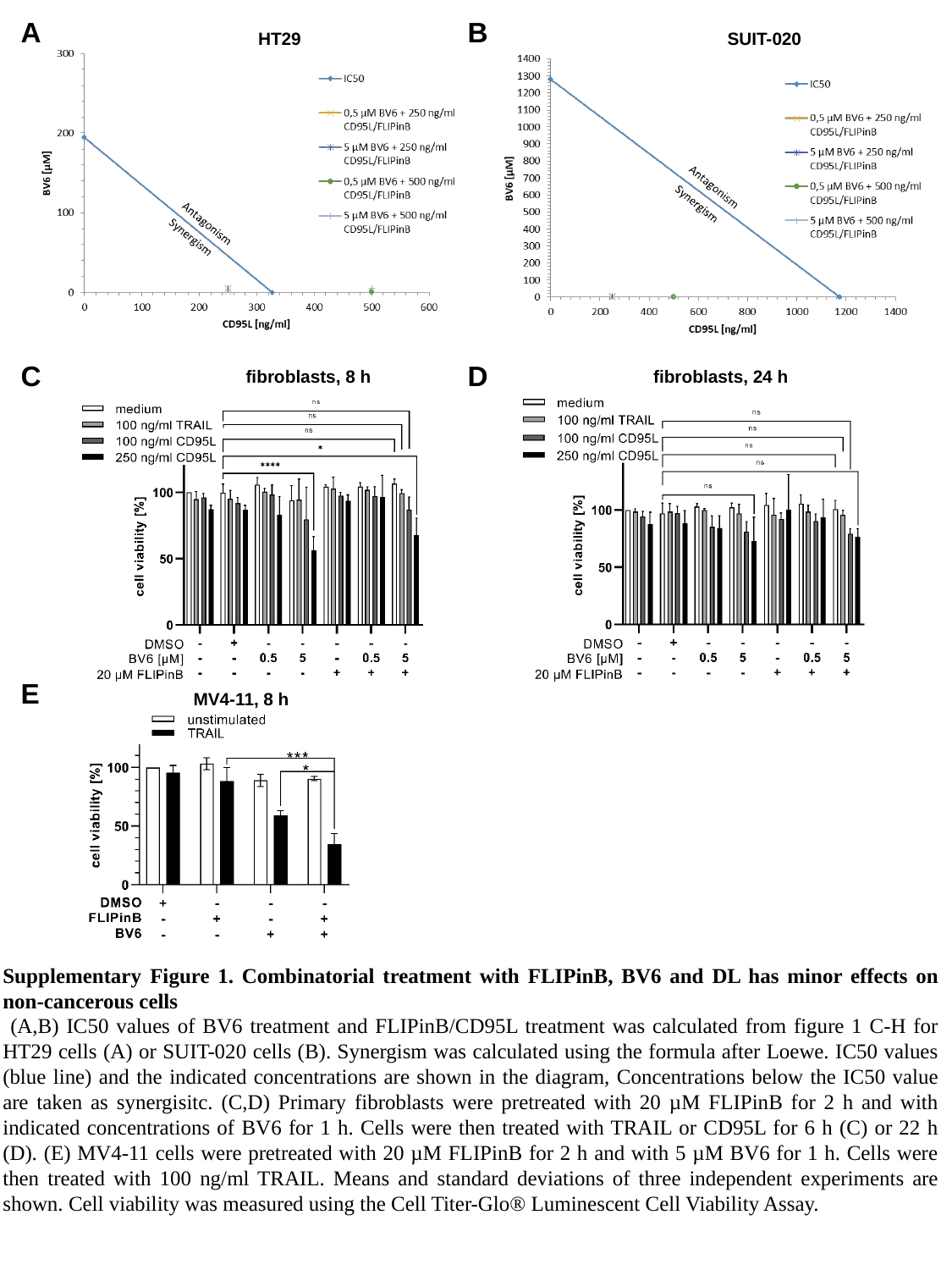

A
B
HT29
SUIT-020
C
D
fibroblasts, 8 h
fibroblasts, 24 h
E
MV4-11, 8 h
Supplementary Figure 1. Combinatorial treatment with FLIPinB, BV6 and DL has minor effects on non-cancerous cells
 (A,B) IC50 values of BV6 treatment and FLIPinB/CD95L treatment was calculated from figure 1 C-H for HT29 cells (A) or SUIT-020 cells (B). Synergism was calculated using the formula after Loewe. IC50 values (blue line) and the indicated concentrations are shown in the diagram, Concentrations below the IC50 value are taken as synergisitc. (C,D) Primary fibroblasts were pretreated with 20 µM FLIPinB for 2 h and with indicated concentrations of BV6 for 1 h. Cells were then treated with TRAIL or CD95L for 6 h (C) or 22 h (D). (E) MV4-11 cells were pretreated with 20 µM FLIPinB for 2 h and with 5 µM BV6 for 1 h. Cells were then treated with 100 ng/ml TRAIL. Means and standard deviations of three independent experiments are shown. Cell viability was measured using the Cell Titer-Glo® Luminescent Cell Viability Assay.

## Slide 2
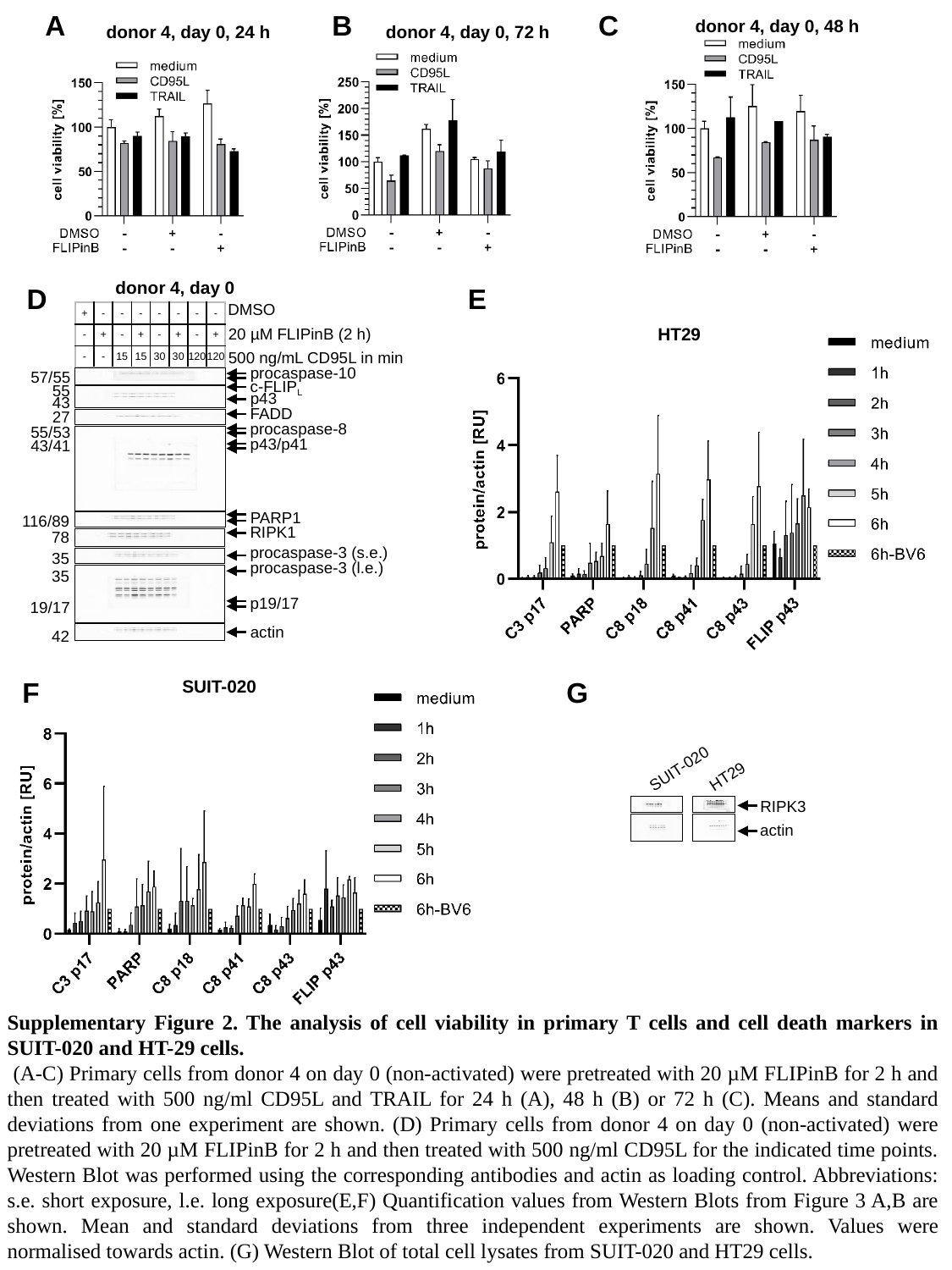

A
B
C
donor 4, day 0, 48 h
donor 4, day 0, 24 h
donor 4, day 0, 72 h
donor 4, day 0
D
E
DMSO
| + | - | - | - | - | - | - | - |
| --- | --- | --- | --- | --- | --- | --- | --- |
| - | + | - | + | - | + | - | + |
| - | - | 15 | 15 | 30 | 30 | 120 | 120 |
HT29
20 µM FLIPinB (2 h)
500 ng/mL CD95L in min
procaspase-10
57/55
c-FLIPL
55
p43
43
FADD
27
procaspase-8
55/53
p43/p41
43/41
PARP1
116/89
RIPK1
78
procaspase-3 (s.e.)
35
procaspase-3 (l.e.)
35
p19/17
19/17
actin
42
F
G
SUIT-020
SUIT-020
HT29
RIPK3
actin
Supplementary Figure 2. The analysis of cell viability in primary T cells and cell death markers in SUIT-020 and HT-29 cells.
 (A-C) Primary cells from donor 4 on day 0 (non-activated) were pretreated with 20 µM FLIPinB for 2 h and then treated with 500 ng/ml CD95L and TRAIL for 24 h (A), 48 h (B) or 72 h (C). Means and standard deviations from one experiment are shown. (D) Primary cells from donor 4 on day 0 (non-activated) were pretreated with 20 µM FLIPinB for 2 h and then treated with 500 ng/ml CD95L for the indicated time points. Western Blot was performed using the corresponding antibodies and actin as loading control. Abbreviations: s.e. short exposure, l.e. long exposure(E,F) Quantification values from Western Blots from Figure 3 A,B are shown. Mean and standard deviations from three independent experiments are shown. Values were normalised towards actin. (G) Western Blot of total cell lysates from SUIT-020 and HT29 cells.

## Slide 3
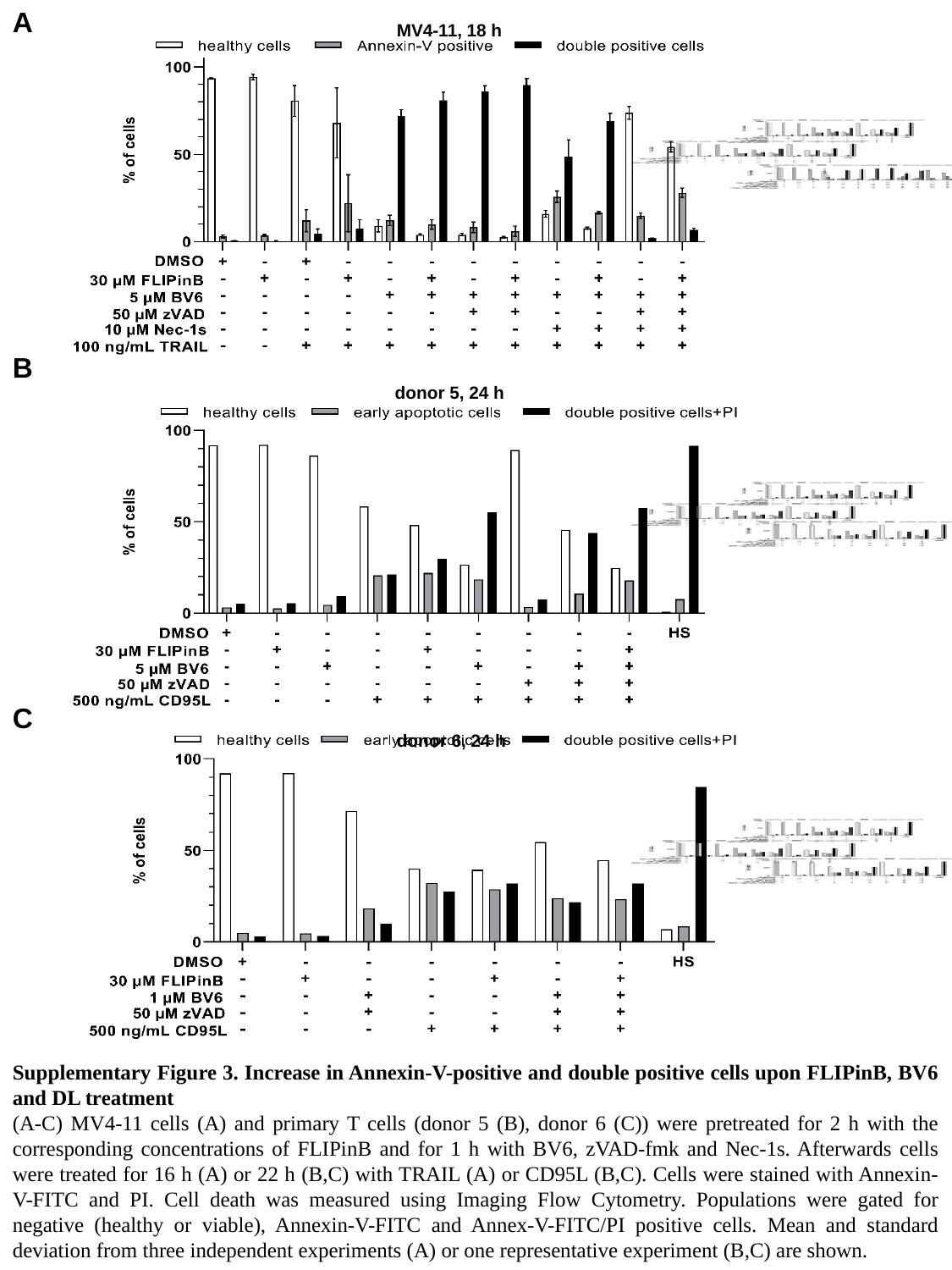

A
MV4-11, 18 h
B
donor 5, 24 h
C
donor 6, 24 h
Supplementary Figure 3. Increase in Annexin-V-positive and double positive cells upon FLIPinB, BV6 and DL treatment
(A-C) MV4-11 cells (A) and primary T cells (donor 5 (B), donor 6 (C)) were pretreated for 2 h with the corresponding concentrations of FLIPinB and for 1 h with BV6, zVAD-fmk and Nec-1s. Afterwards cells were treated for 16 h (A) or 22 h (B,C) with TRAIL (A) or CD95L (B,C). Cells were stained with Annexin-V-FITC and PI. Cell death was measured using Imaging Flow Cytometry. Populations were gated for negative (healthy or viable), Annexin-V-FITC and Annex-V-FITC/PI positive cells. Mean and standard deviation from three independent experiments (A) or one representative experiment (B,C) are shown.

## Slide 4
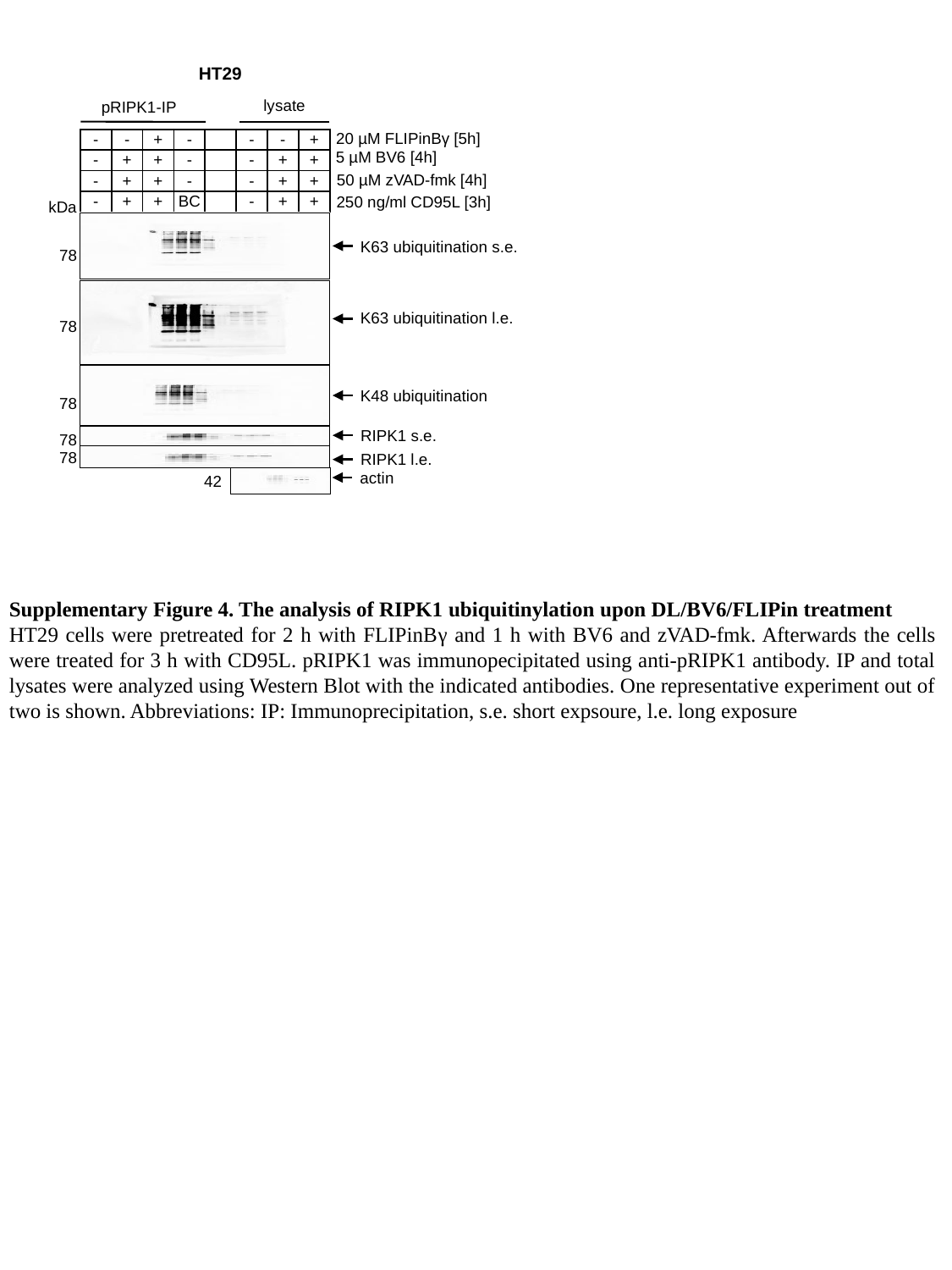

HT29
lysate
pRIPK1-IP
20 µM FLIPinBγ [5h]
| - | - | + | - | | - | - | + |
| --- | --- | --- | --- | --- | --- | --- | --- |
| - | + | + | - | | - | + | + |
| - | + | + | - | | - | + | + |
| - | + | + | BC | | - | + | + |
5 µM BV6 [4h]
50 µM zVAD-fmk [4h]
250 ng/ml CD95L [3h]
kDa
K63 ubiquitination s.e.
78
K63 ubiquitination l.e.
78
K48 ubiquitination
78
RIPK1 s.e.
78
78
RIPK1 l.e.
actin
42
Supplementary Figure 4. The analysis of RIPK1 ubiquitinylation upon DL/BV6/FLIPin treatment
HT29 cells were pretreated for 2 h with FLIPinBγ and 1 h with BV6 and zVAD-fmk. Afterwards the cells were treated for 3 h with CD95L. pRIPK1 was immunopecipitated using anti-pRIPK1 antibody. IP and total lysates were analyzed using Western Blot with the indicated antibodies. One representative experiment out of two is shown. Abbreviations: IP: Immunoprecipitation, s.e. short expsoure, l.e. long exposure

## Slide 5
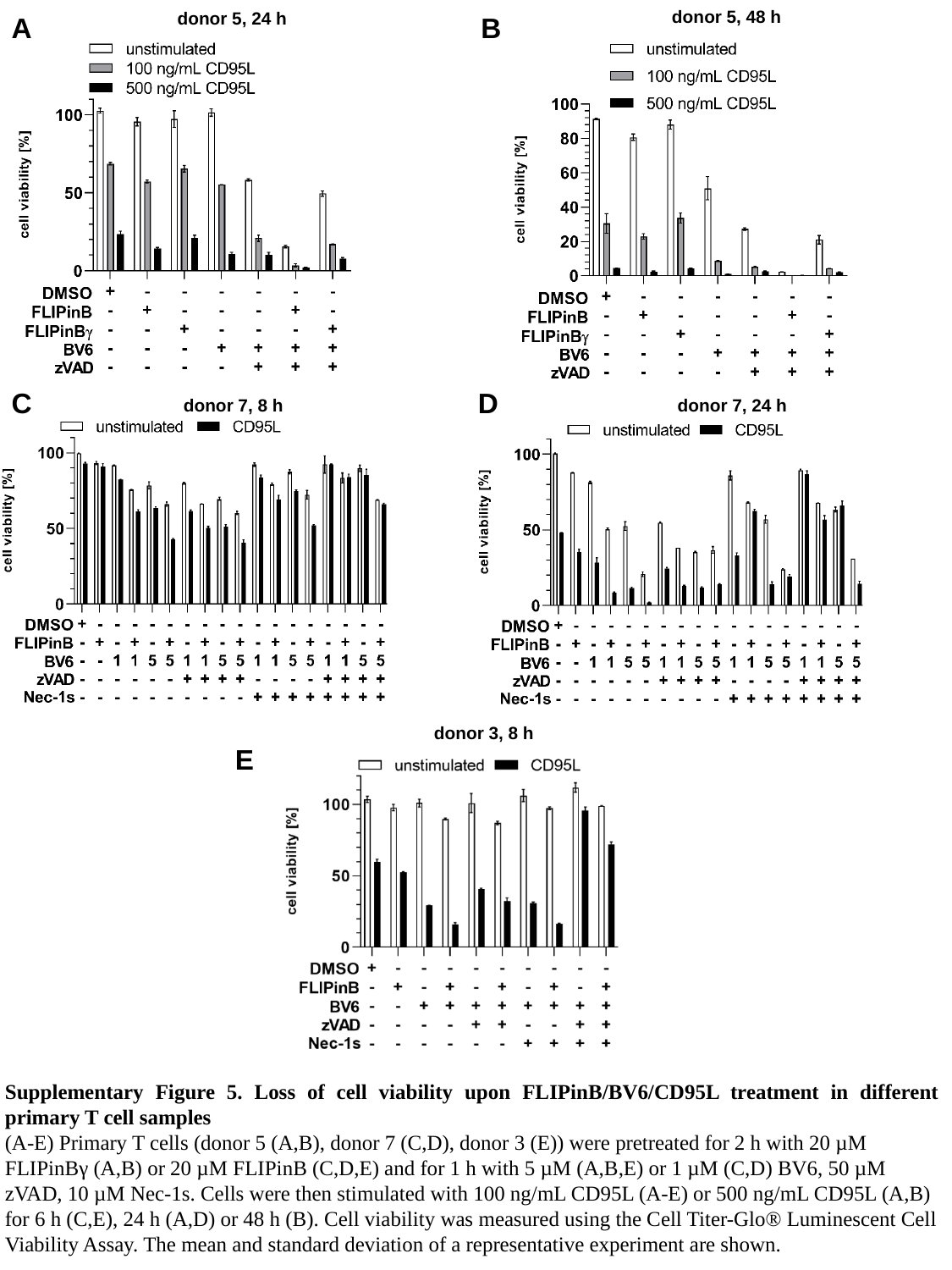

donor 5, 48 h
donor 5, 24 h
A
B
C
D
donor 7, 8 h
donor 7, 24 h
donor 3, 8 h
E
Supplementary Figure 5. Loss of cell viability upon FLIPinB/BV6/CD95L treatment in different primary T cell samples
(A-E) Primary T cells (donor 5 (A,B), donor 7 (C,D), donor 3 (E)) were pretreated for 2 h with 20 µM FLIPinBγ (A,B) or 20 µM FLIPinB (C,D,E) and for 1 h with 5 µM (A,B,E) or 1 µM (C,D) BV6, 50 µM zVAD, 10 µM Nec-1s. Cells were then stimulated with 100 ng/mL CD95L (A-E) or 500 ng/mL CD95L (A,B) for 6 h (C,E), 24 h (A,D) or 48 h (B). Cell viability was measured using the Cell Titer-Glo® Luminescent Cell Viability Assay. The mean and standard deviation of a representative experiment are shown.
