# Supplementary figures and images for: Targeting caspase-8/c-FLIPL heterodimer in complex II promotes DL-mediated cell death
Source: Front Cell Dev Biol. 2024 Sep 30;12:1471216. doi: 10.3389/fcell.2024.1471216 (PMC11471875; doi:10.3389/fcell.2024.1471216)

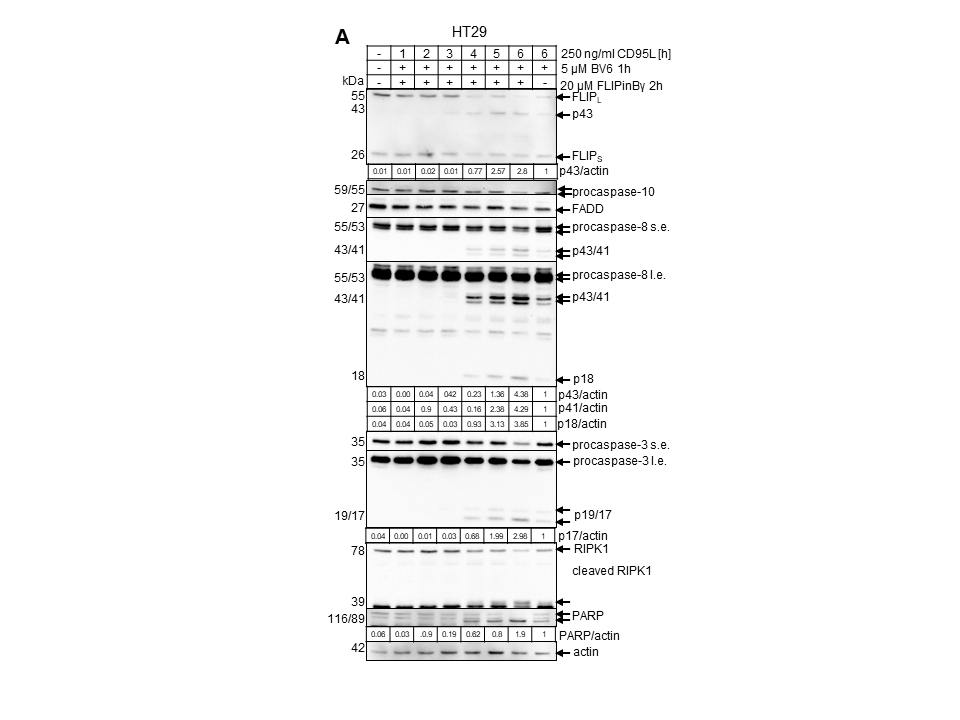

Supplement: Supplementary file 2 [file Presentation2.zip › figure3a-HT29-rawdata/Folie1.TIF]

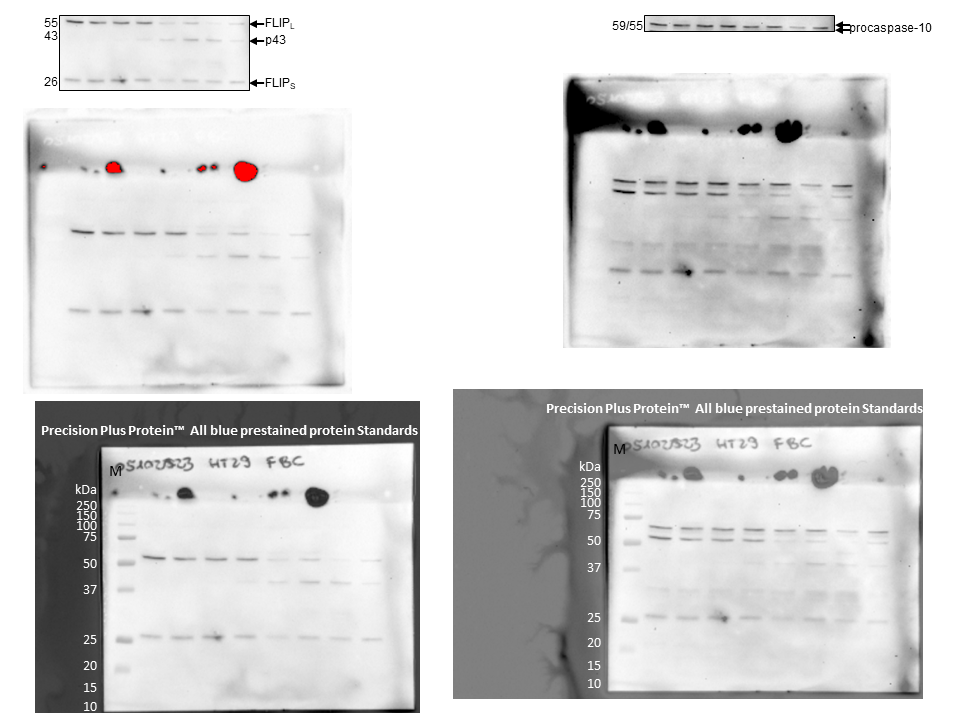

Supplement: Supplementary file 2 [file Presentation2.zip › figure3a-HT29-rawdata/Folie2.TIF]

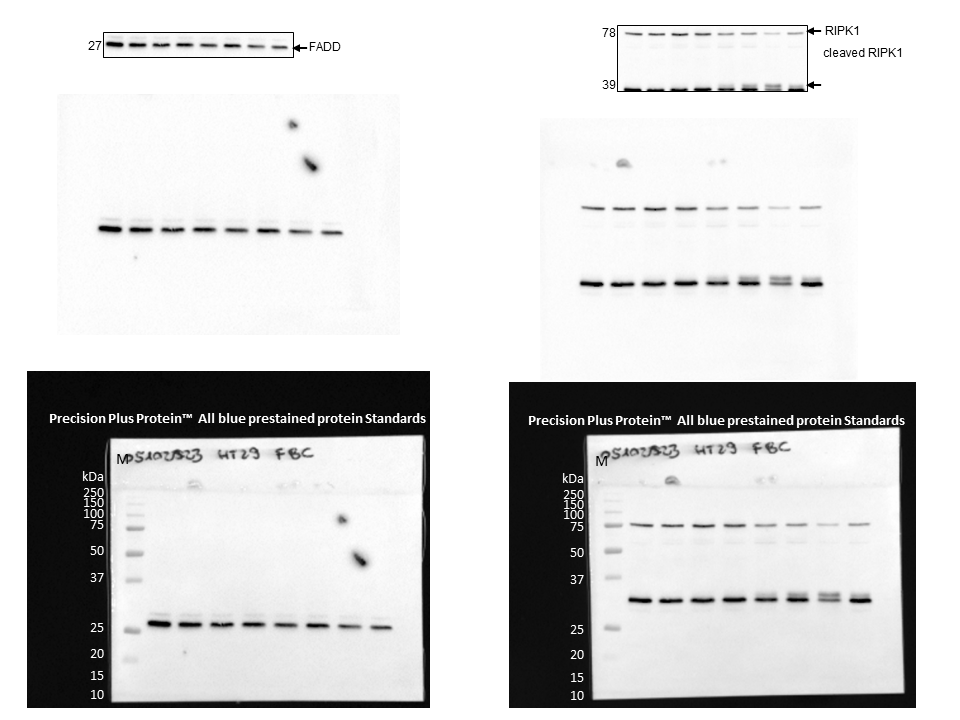

Supplement: Supplementary file 2 [file Presentation2.zip › figure3a-HT29-rawdata/Folie3.TIF]

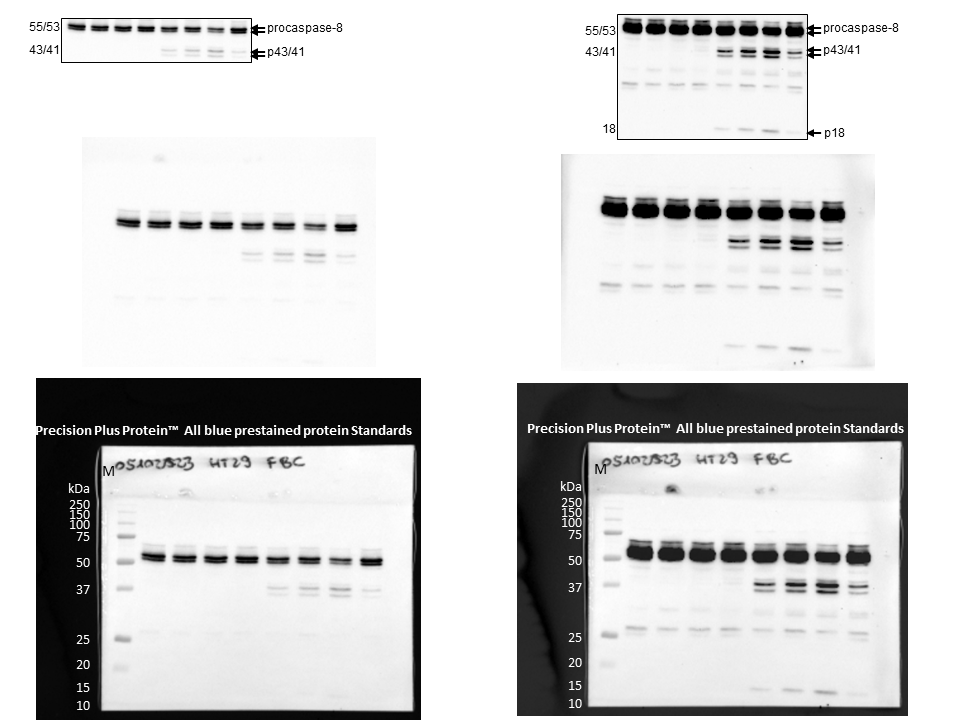

Supplement: Supplementary file 2 [file Presentation2.zip › figure3a-HT29-rawdata/Folie4.TIF]

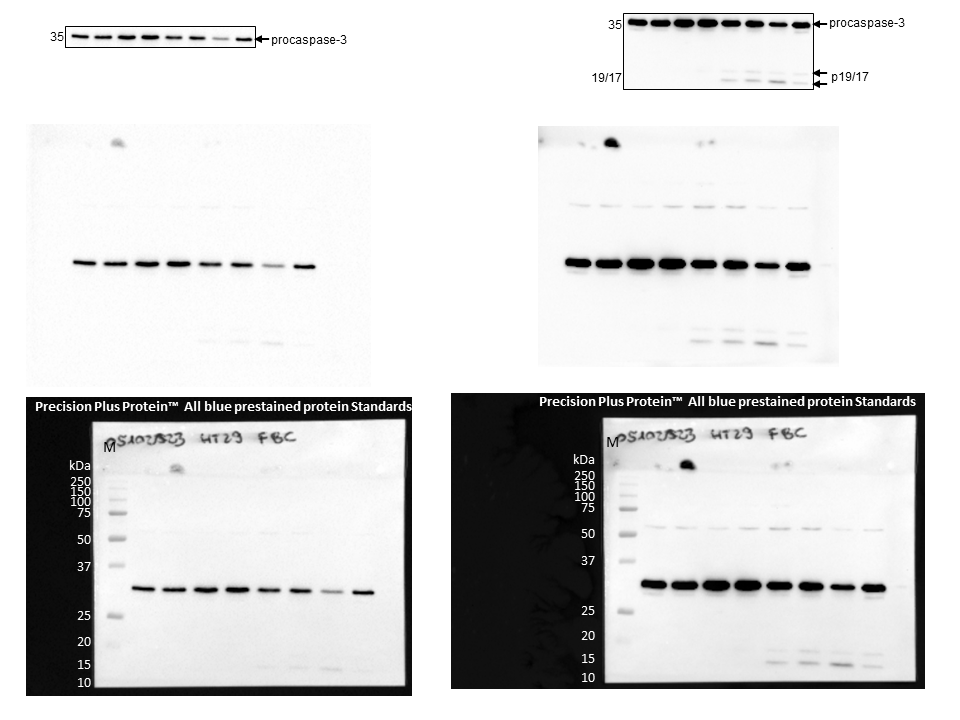

Supplement: Supplementary file 2 [file Presentation2.zip › figure3a-HT29-rawdata/Folie5.TIF]

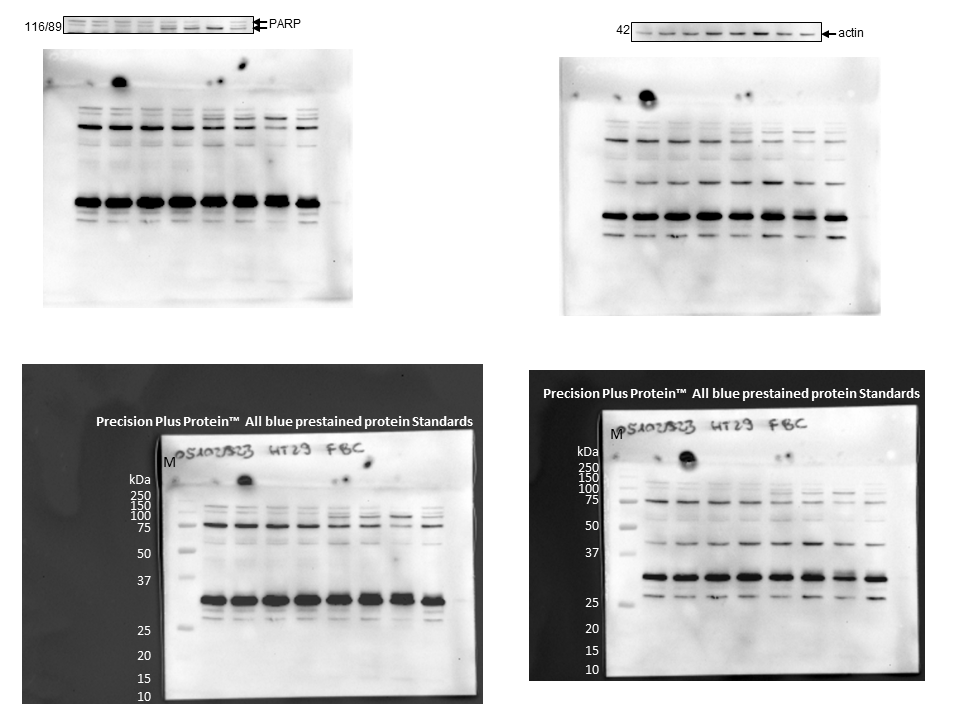

Supplement: Supplementary file 2 [file Presentation2.zip › figure3a-HT29-rawdata/Folie6.TIF]

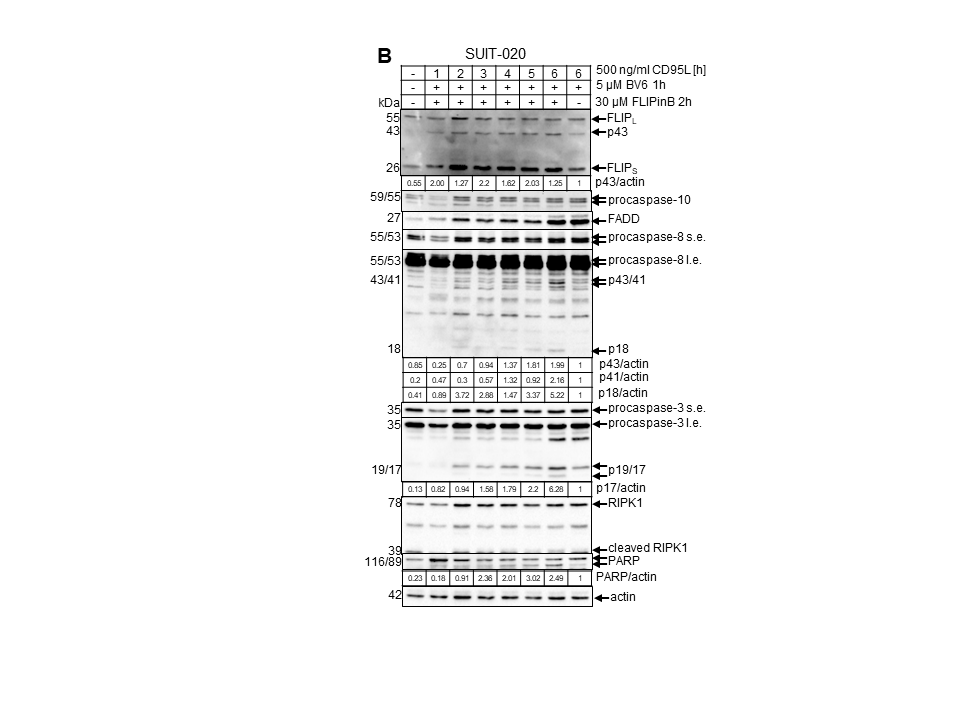

Supplement: Supplementary file 2 [file Presentation2.zip › figure3b-suit-rawdata/Folie1.TIF]

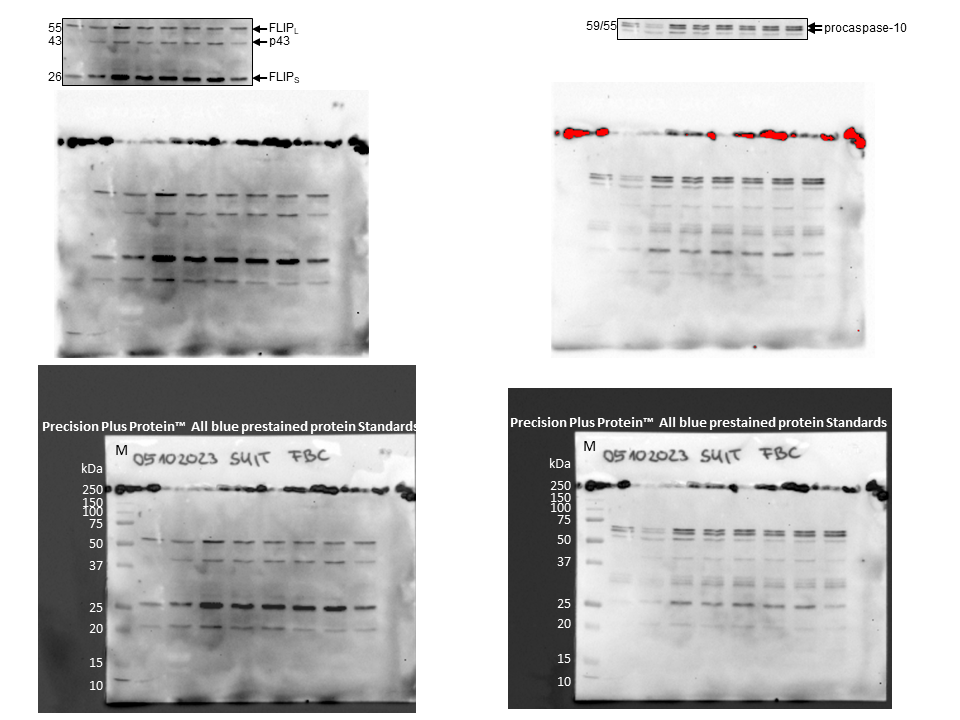

Supplement: Supplementary file 2 [file Presentation2.zip › figure3b-suit-rawdata/Folie2.TIF]

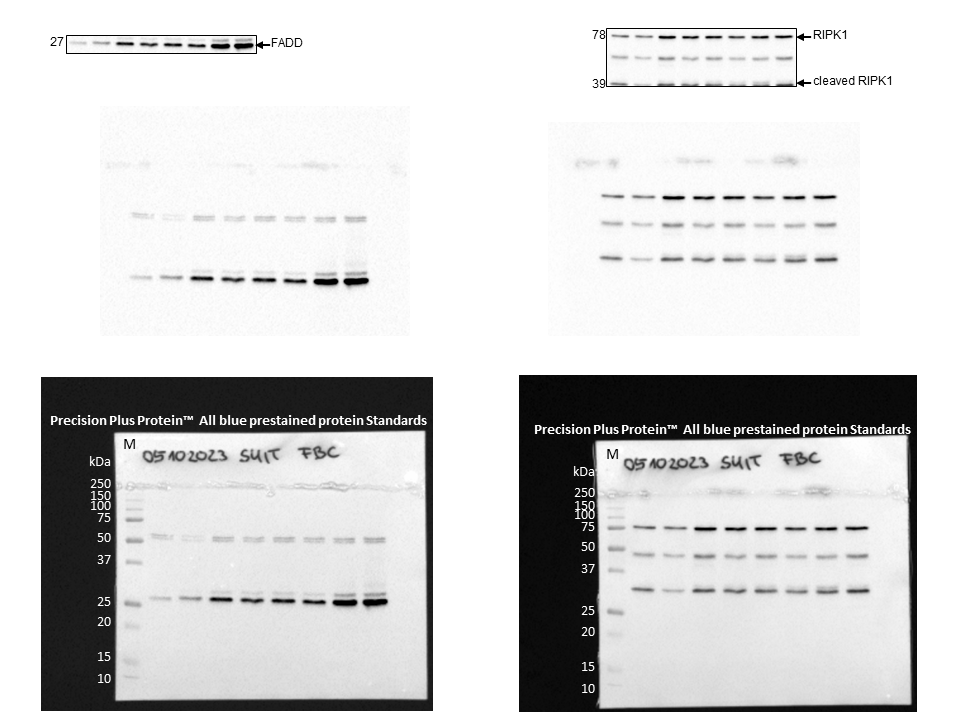

Supplement: Supplementary file 2 [file Presentation2.zip › figure3b-suit-rawdata/Folie3.TIF]

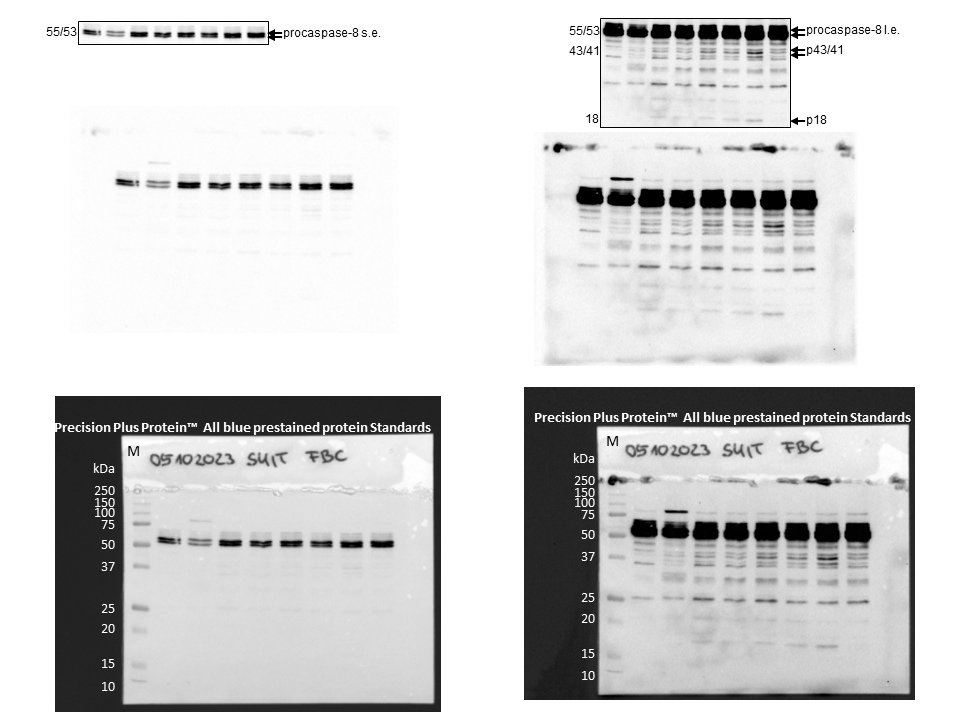

Supplement: Supplementary file 2 [file Presentation2.zip › figure3b-suit-rawdata/Folie4.TIF]

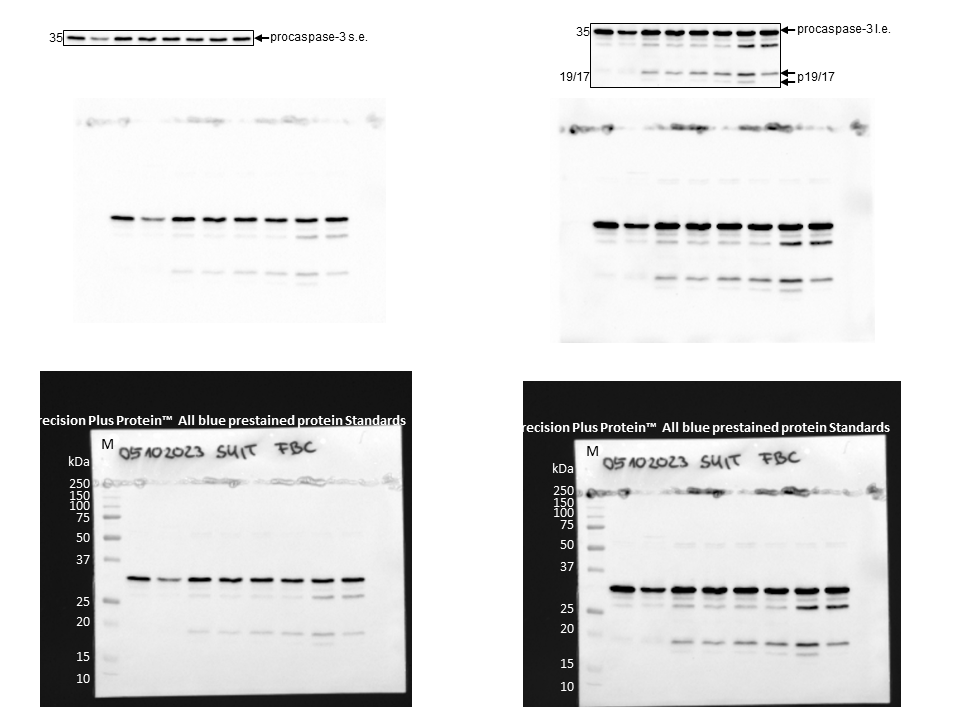

Supplement: Supplementary file 2 [file Presentation2.zip › figure3b-suit-rawdata/Folie5.TIF]

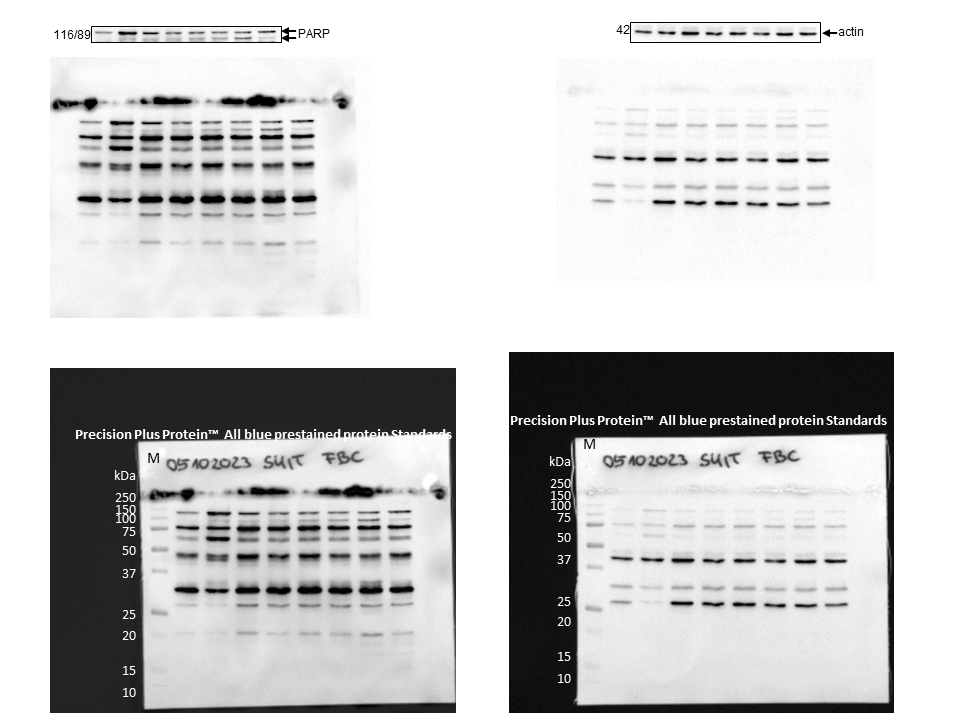

Supplement: Supplementary file 2 [file Presentation2.zip › figure3b-suit-rawdata/Folie6.TIF]

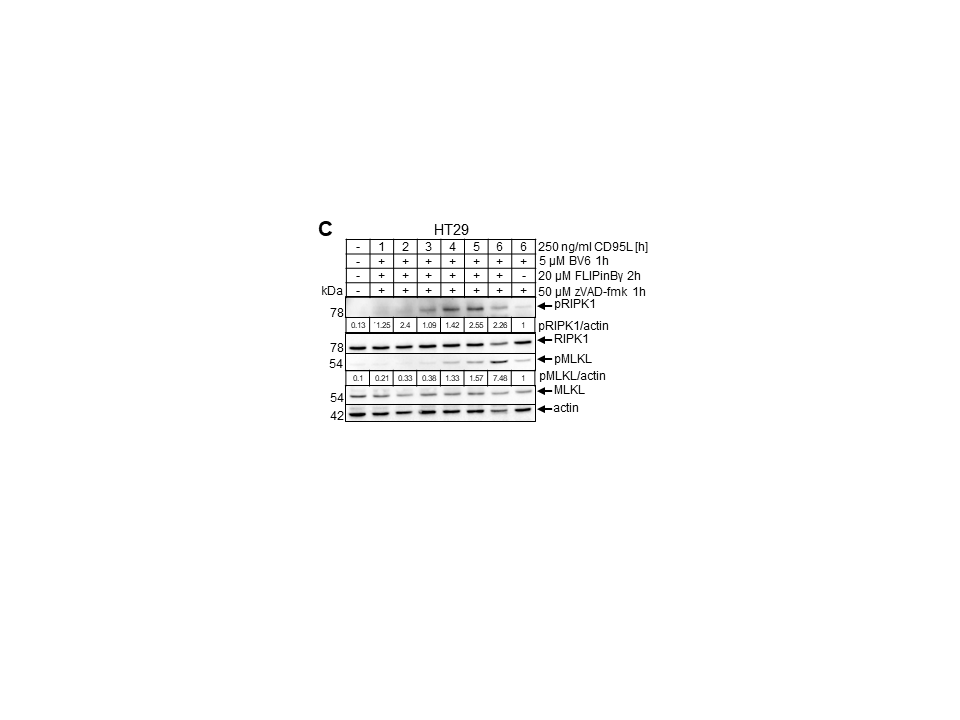

Supplement: Supplementary file 2 [file Presentation2.zip › figure4a-rawdata-HT29-zvad/Folie1.TIF]

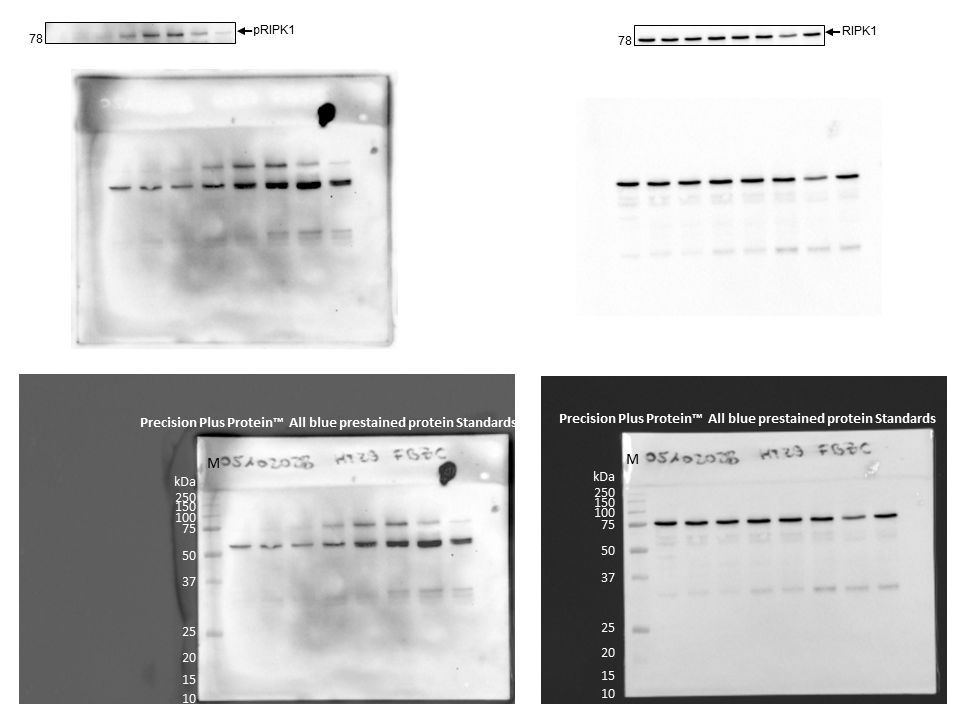

Supplement: Supplementary file 2 [file Presentation2.zip › figure4a-rawdata-HT29-zvad/Folie2.TIF]

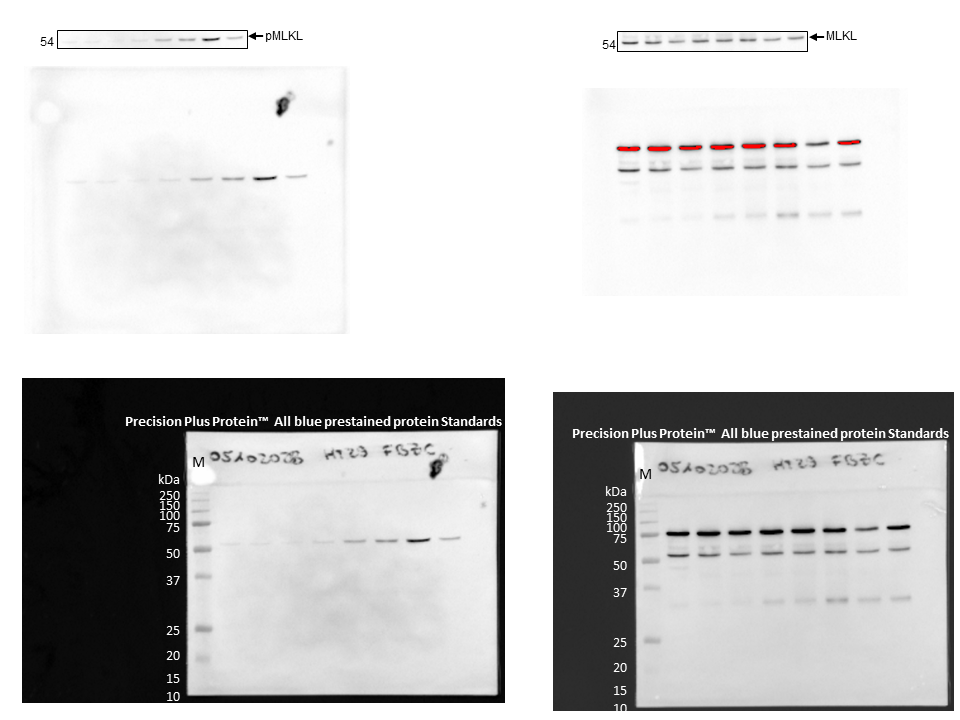

Supplement: Supplementary file 2 [file Presentation2.zip › figure4a-rawdata-HT29-zvad/Folie3.TIF]

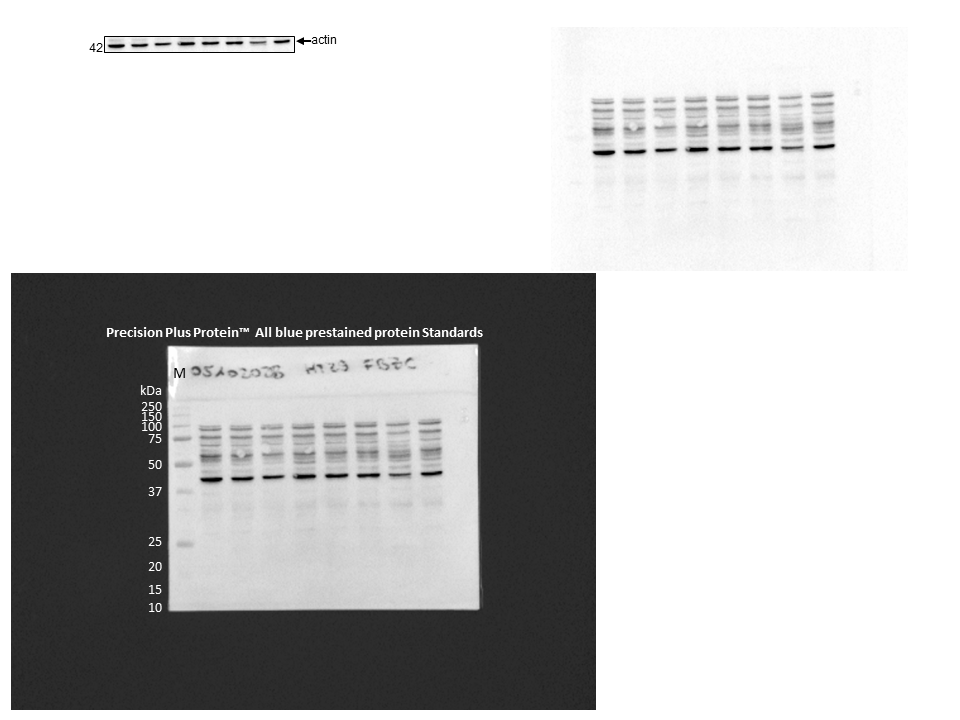

Supplement: Supplementary file 2 [file Presentation2.zip › figure4a-rawdata-HT29-zvad/Folie4.TIF]

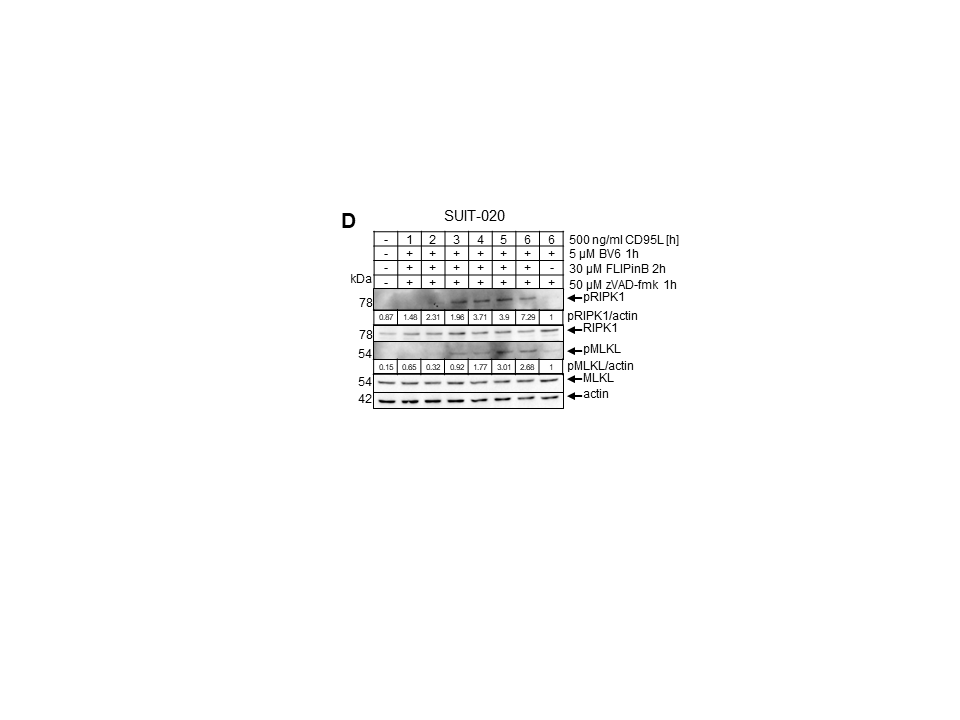

Supplement: Supplementary file 2 [file Presentation2.zip › figure4b-SUIT-zVAD/Folie1.TIF]

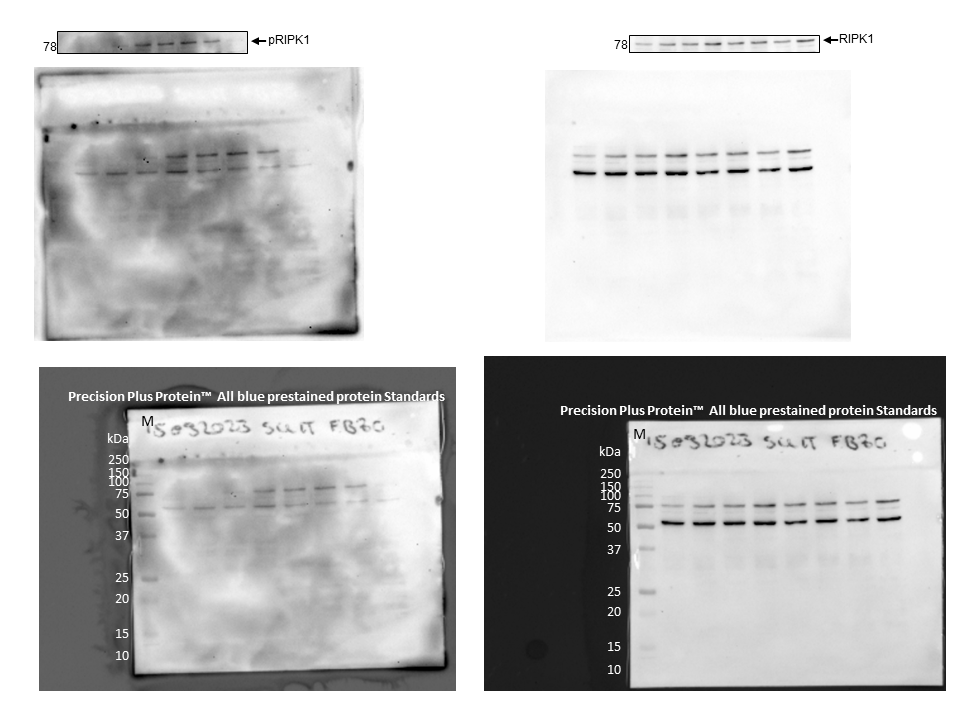

Supplement: Supplementary file 2 [file Presentation2.zip › figure4b-SUIT-zVAD/Folie2.TIF]

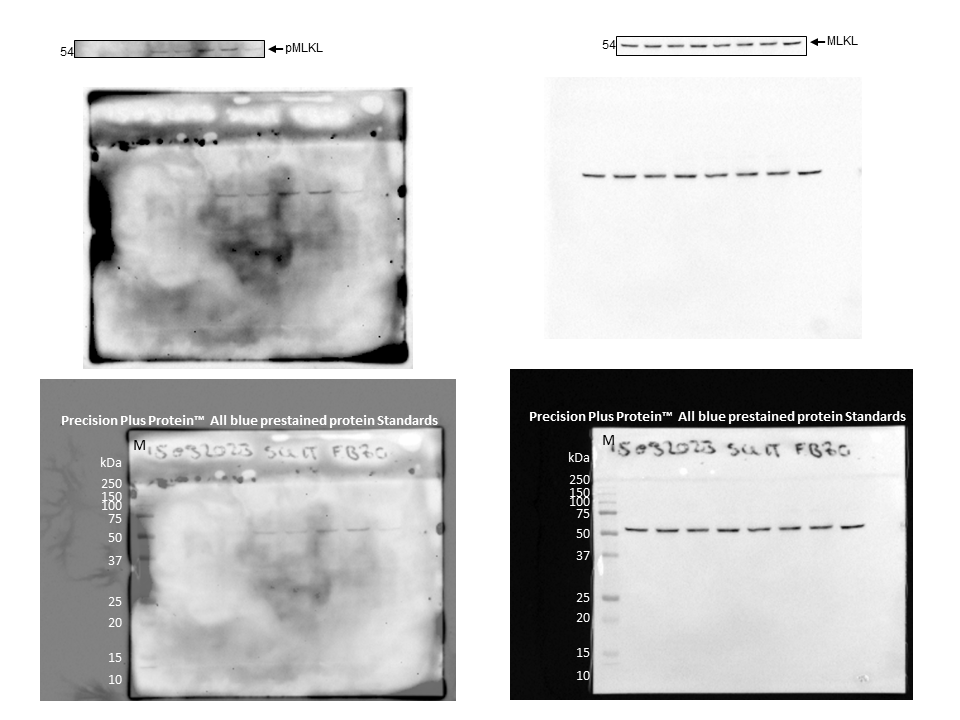

Supplement: Supplementary file 2 [file Presentation2.zip › figure4b-SUIT-zVAD/Folie3.TIF]

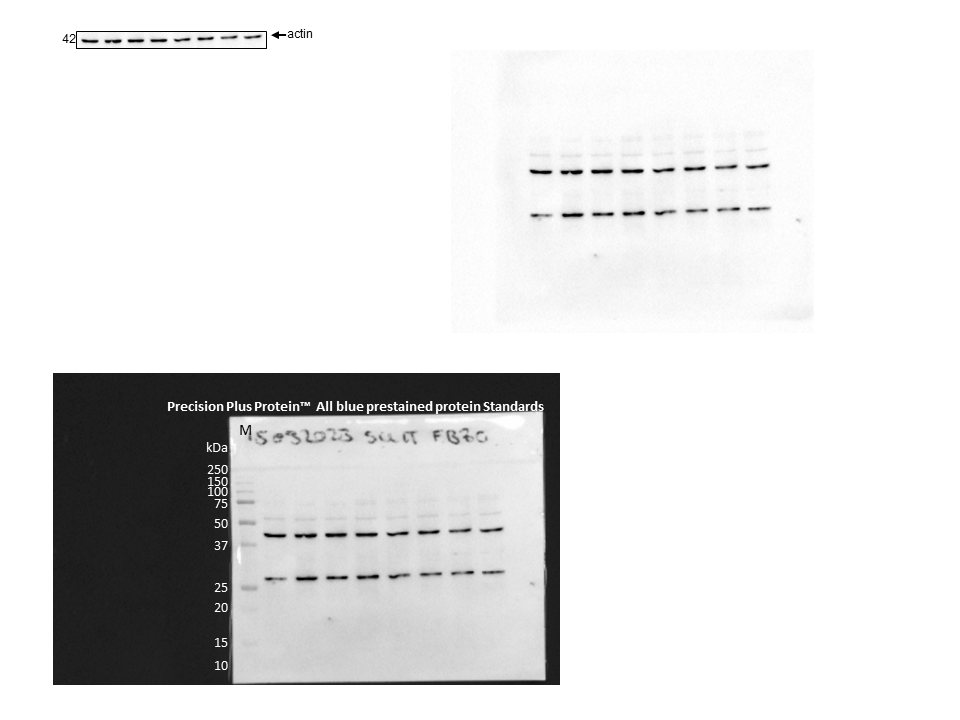

Supplement: Supplementary file 2 [file Presentation2.zip › figure4b-SUIT-zVAD/Folie4.TIF]

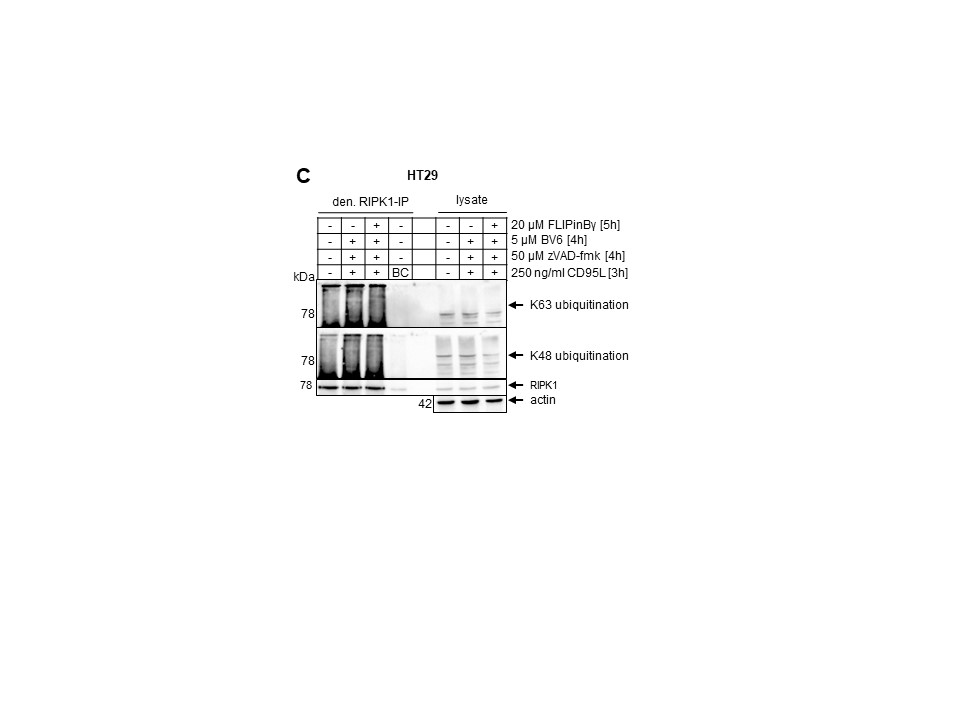

Supplement: Supplementary file 2 [file Presentation2.zip › figure4c-denRIPK1IP/Folie1.JPG]

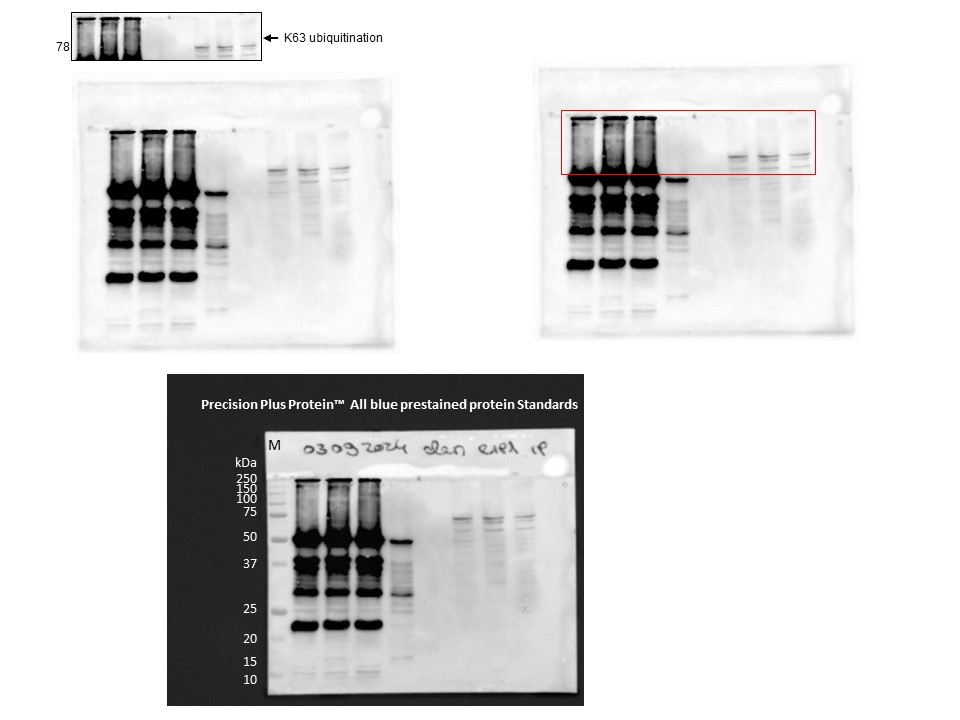

Supplement: Supplementary file 2 [file Presentation2.zip › figure4c-denRIPK1IP/Folie2.JPG]

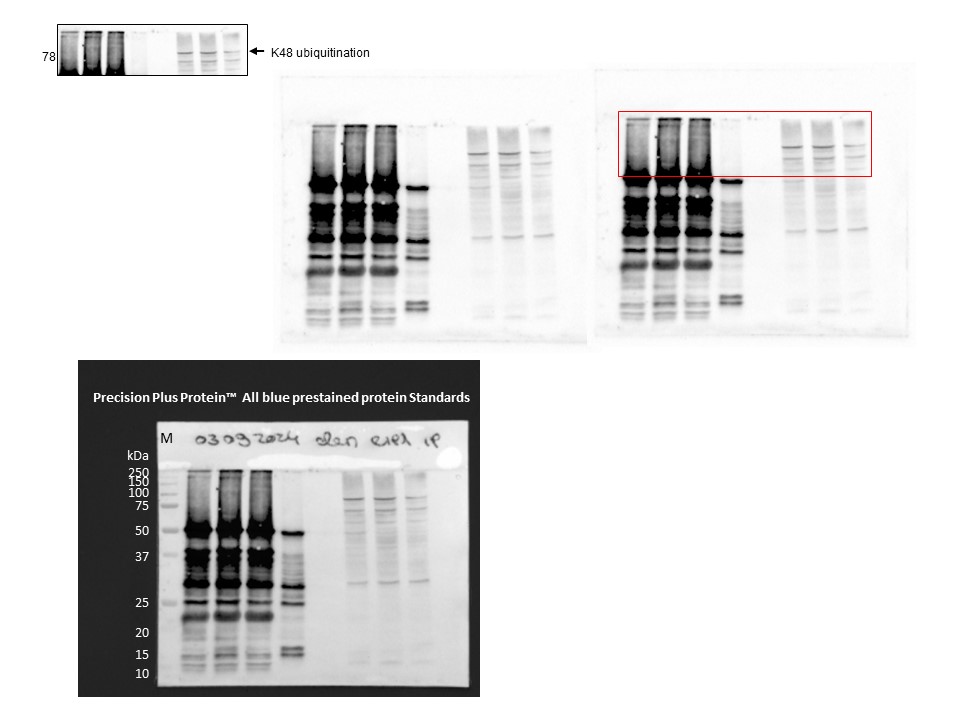

Supplement: Supplementary file 2 [file Presentation2.zip › figure4c-denRIPK1IP/Folie3.JPG]

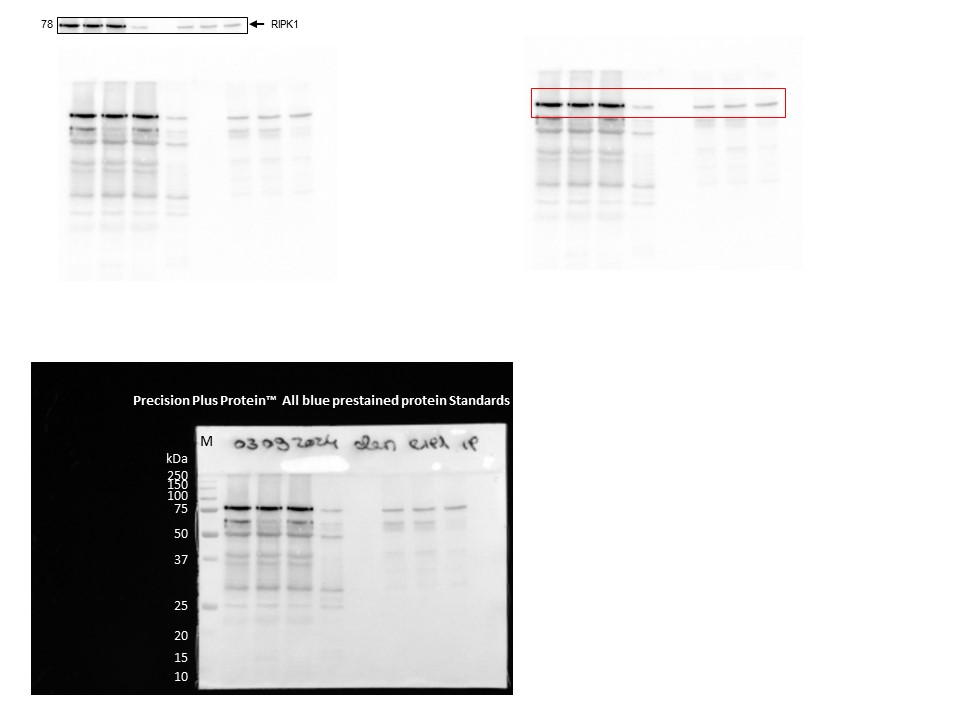

Supplement: Supplementary file 2 [file Presentation2.zip › figure4c-denRIPK1IP/Folie4.JPG]

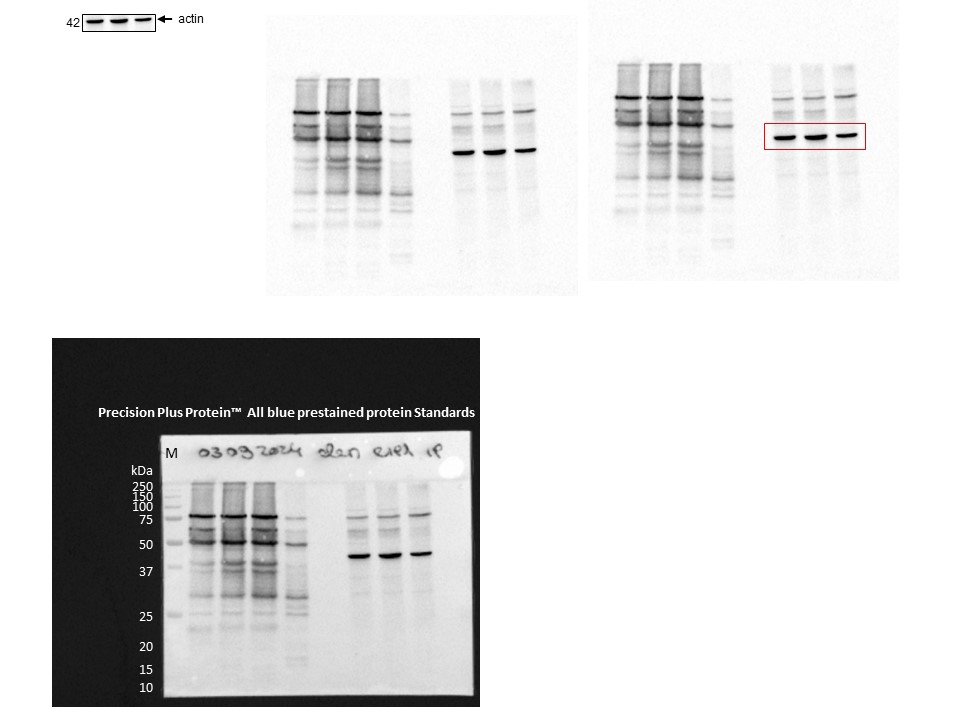

Supplement: Supplementary file 2 [file Presentation2.zip › figure4c-denRIPK1IP/Folie5.JPG]

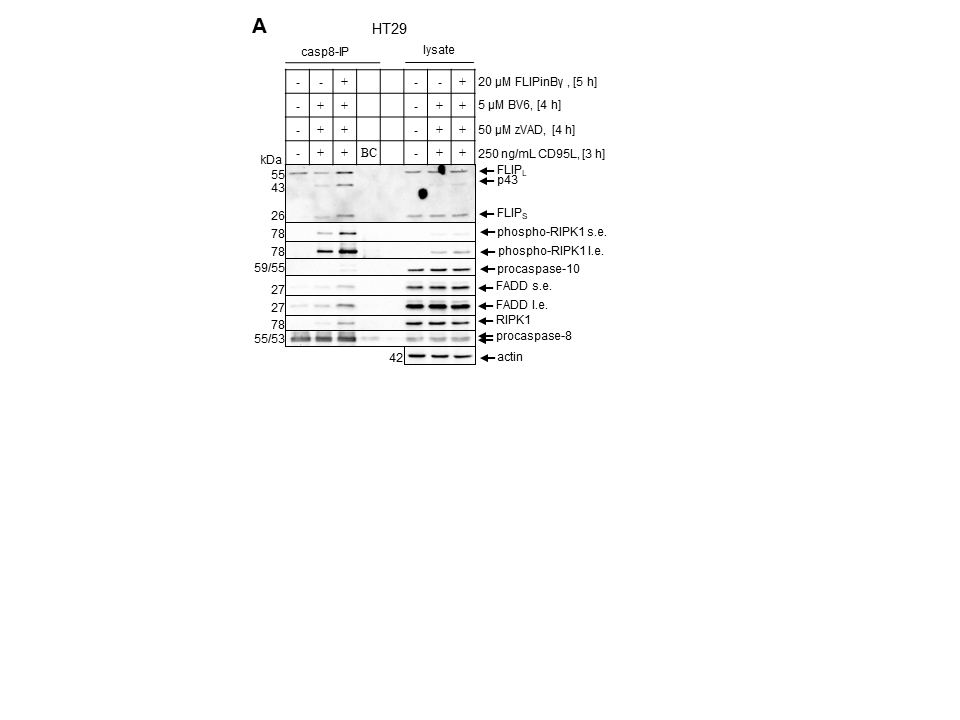

Supplement: Supplementary file 2 [file Presentation2.zip › figure6a-HT29-C8IP-rawdata/Folie1.TIF]

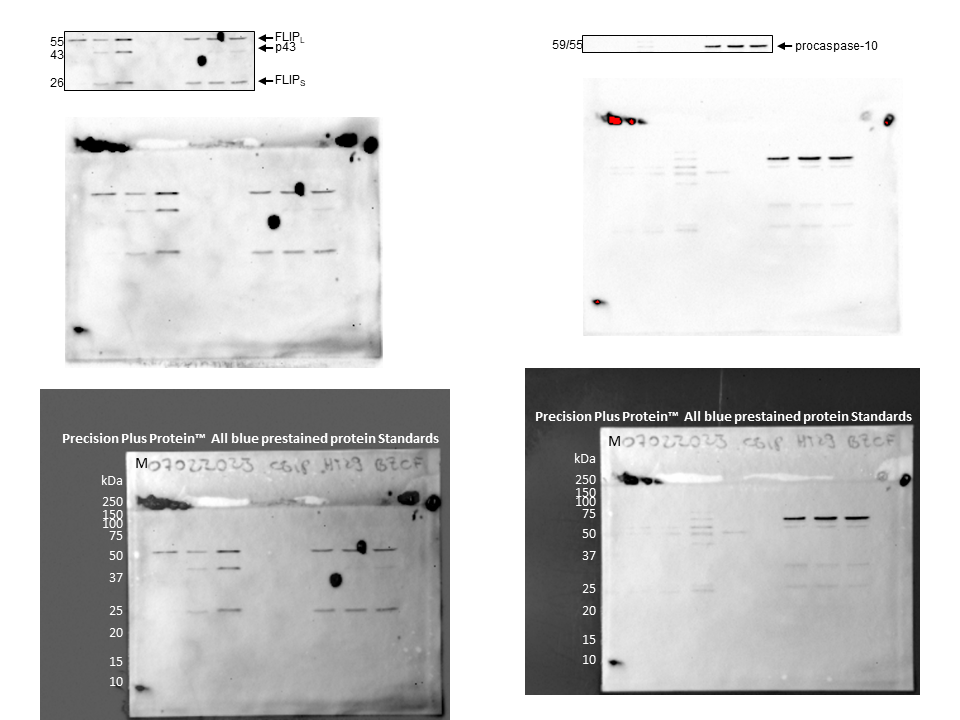

Supplement: Supplementary file 2 [file Presentation2.zip › figure6a-HT29-C8IP-rawdata/Folie2.TIF]

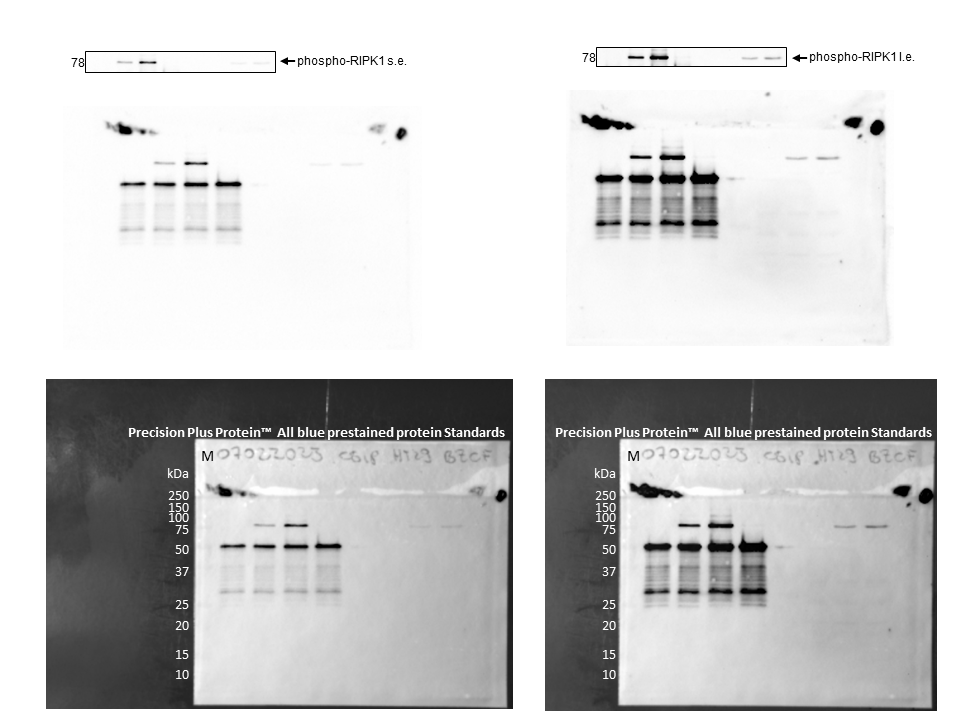

Supplement: Supplementary file 2 [file Presentation2.zip › figure6a-HT29-C8IP-rawdata/Folie3.TIF]

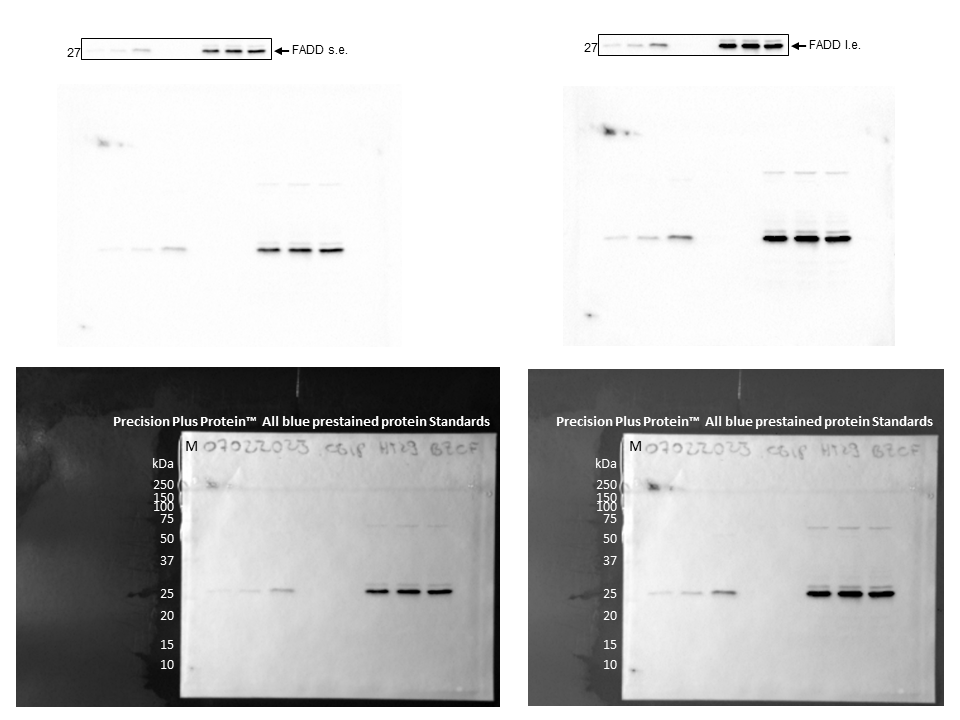

Supplement: Supplementary file 2 [file Presentation2.zip › figure6a-HT29-C8IP-rawdata/Folie4.TIF]

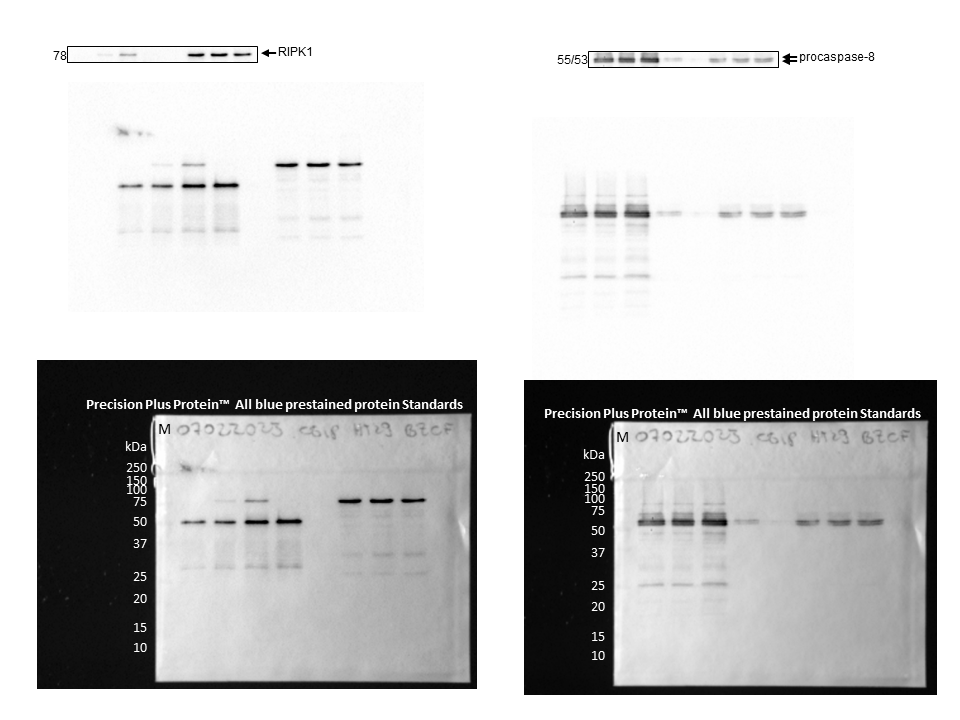

Supplement: Supplementary file 2 [file Presentation2.zip › figure6a-HT29-C8IP-rawdata/Folie5.TIF]

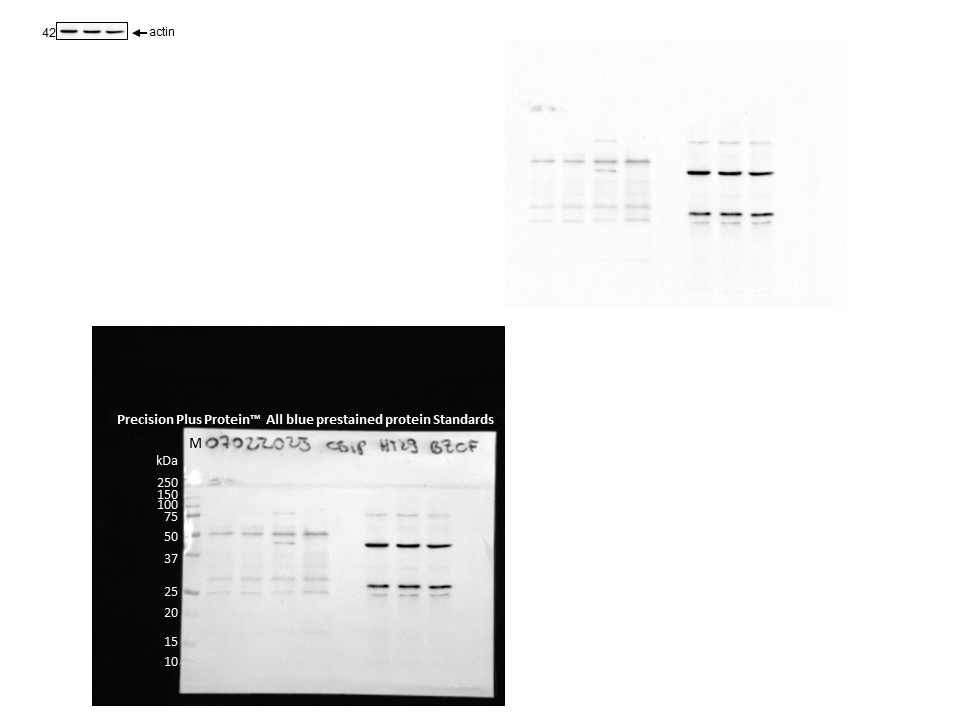

Supplement: Supplementary file 2 [file Presentation2.zip › figure6a-HT29-C8IP-rawdata/Folie6.TIF]

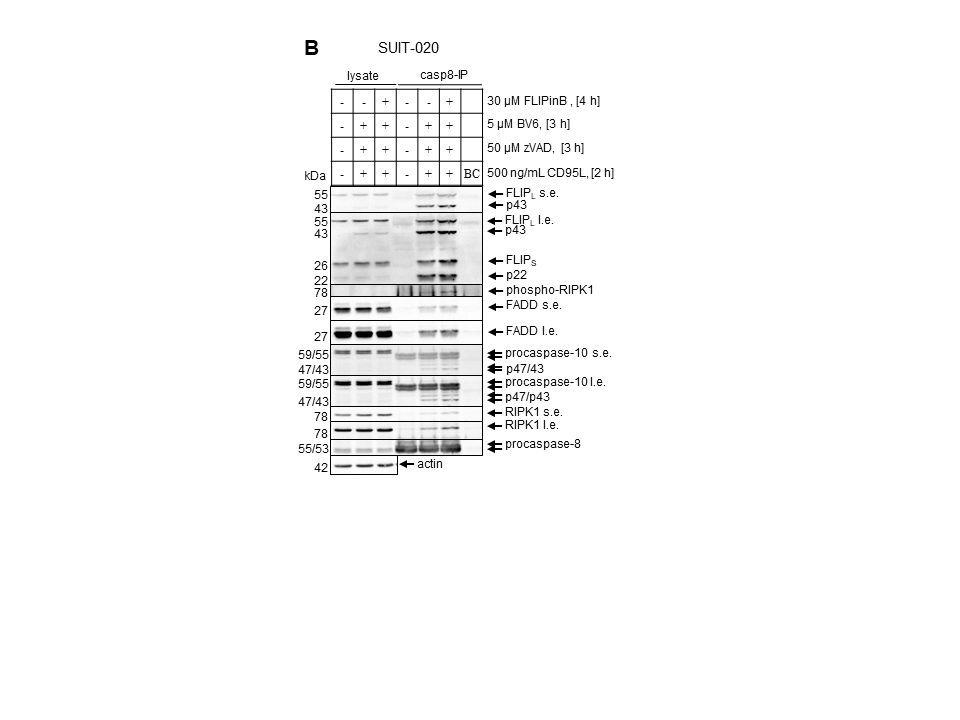

Supplement: Supplementary file 2 [file Presentation2.zip › figure6b-SUIT-C8IP/Folie1.TIF]

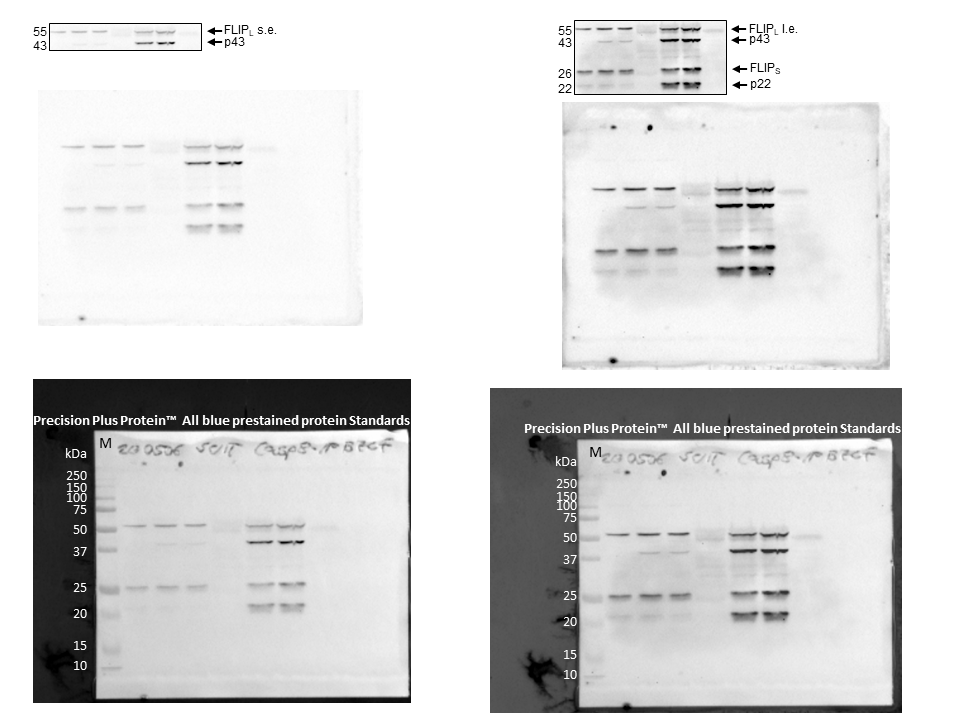

Supplement: Supplementary file 2 [file Presentation2.zip › figure6b-SUIT-C8IP/Folie2.TIF]

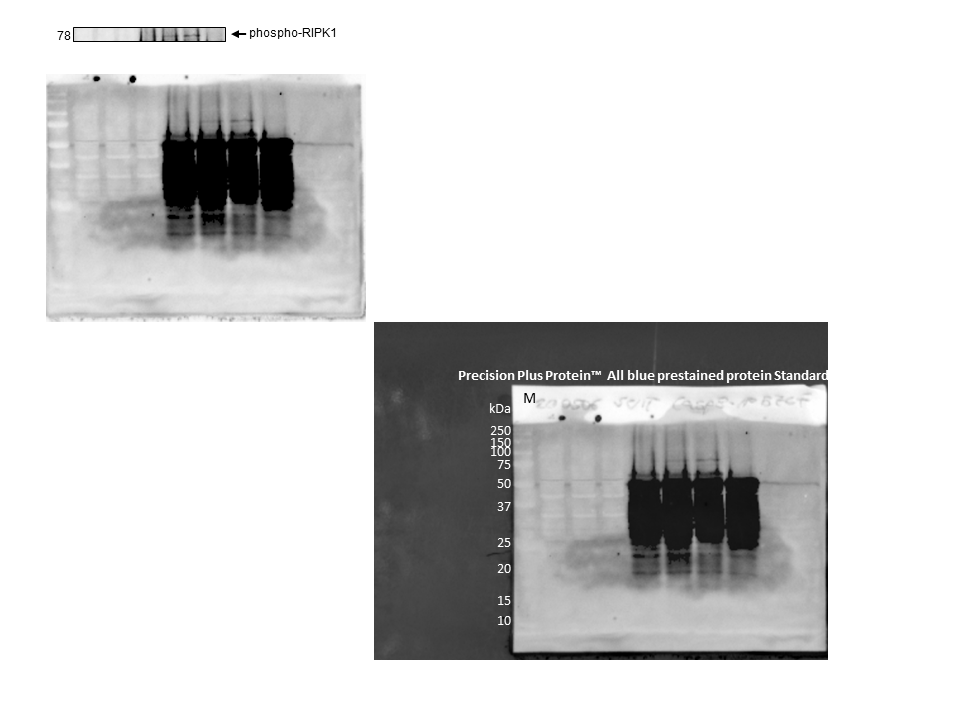

Supplement: Supplementary file 2 [file Presentation2.zip › figure6b-SUIT-C8IP/Folie3.TIF]

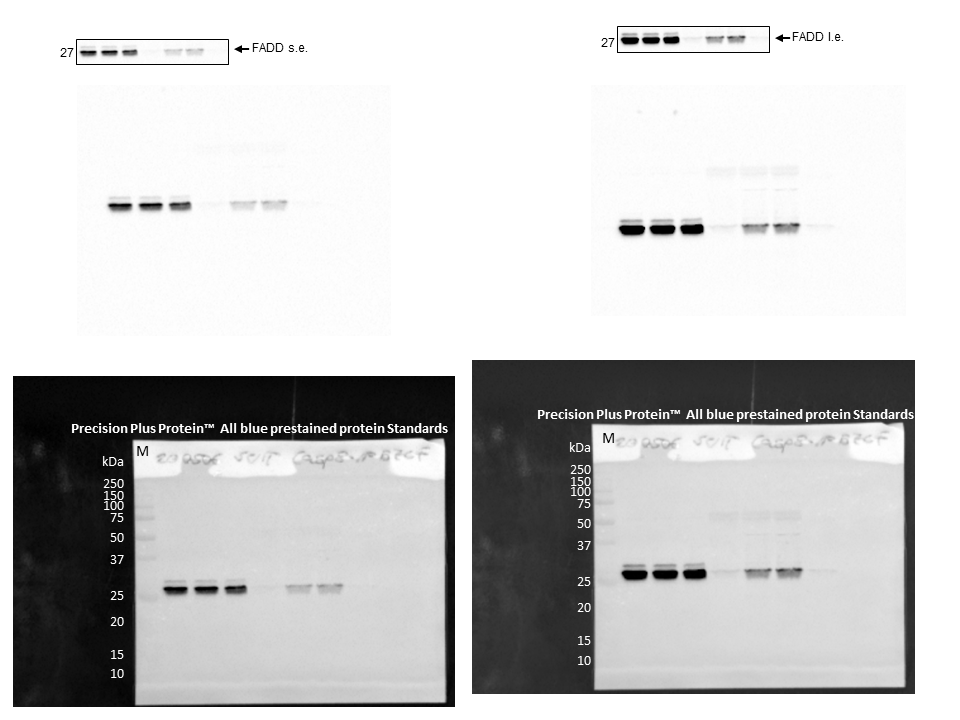

Supplement: Supplementary file 2 [file Presentation2.zip › figure6b-SUIT-C8IP/Folie4.TIF]

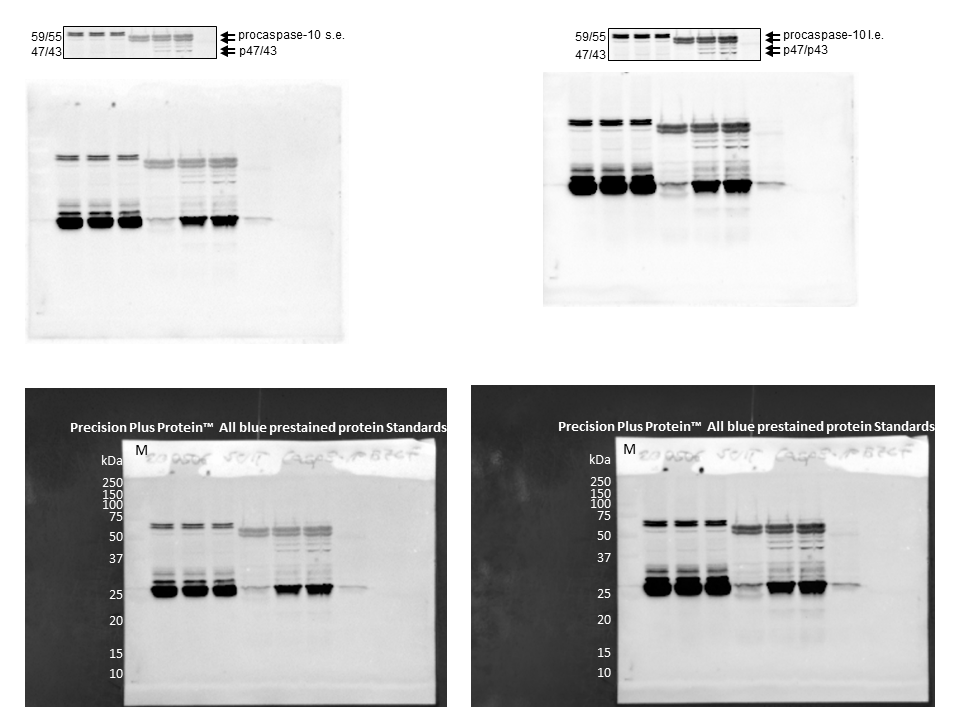

Supplement: Supplementary file 2 [file Presentation2.zip › figure6b-SUIT-C8IP/Folie5.TIF]

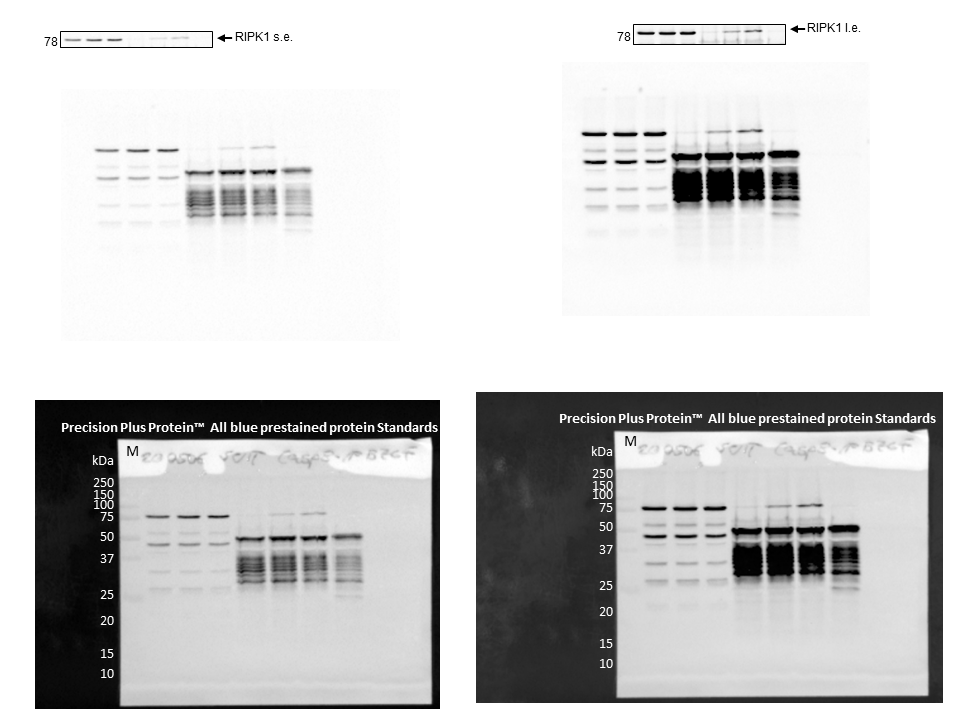

Supplement: Supplementary file 2 [file Presentation2.zip › figure6b-SUIT-C8IP/Folie6.TIF]

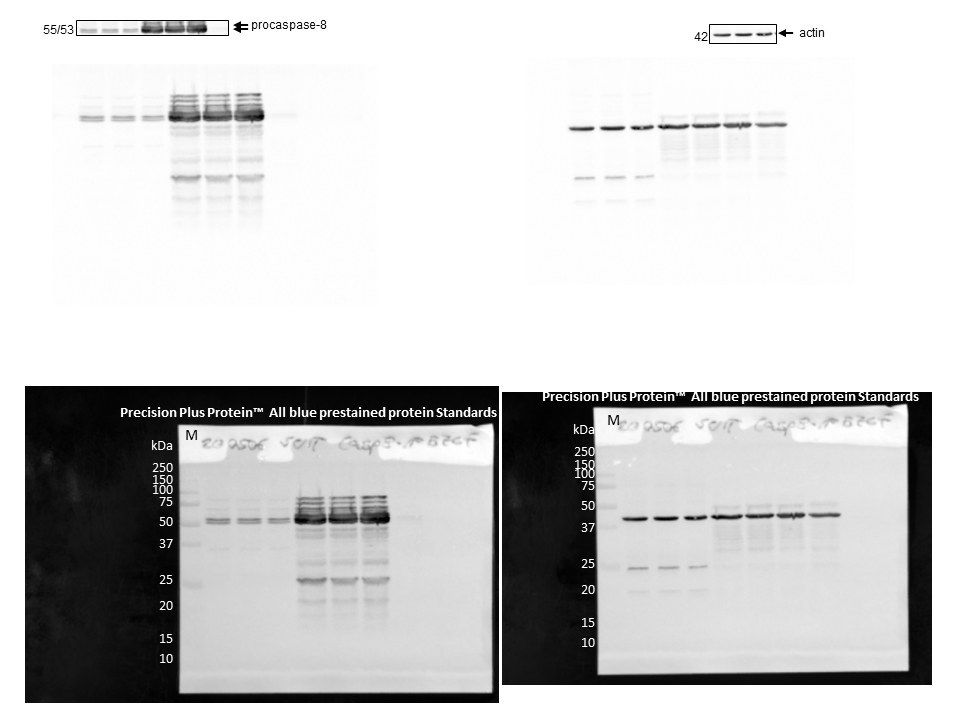

Supplement: Supplementary file 2 [file Presentation2.zip › figure6b-SUIT-C8IP/Folie7.TIF]

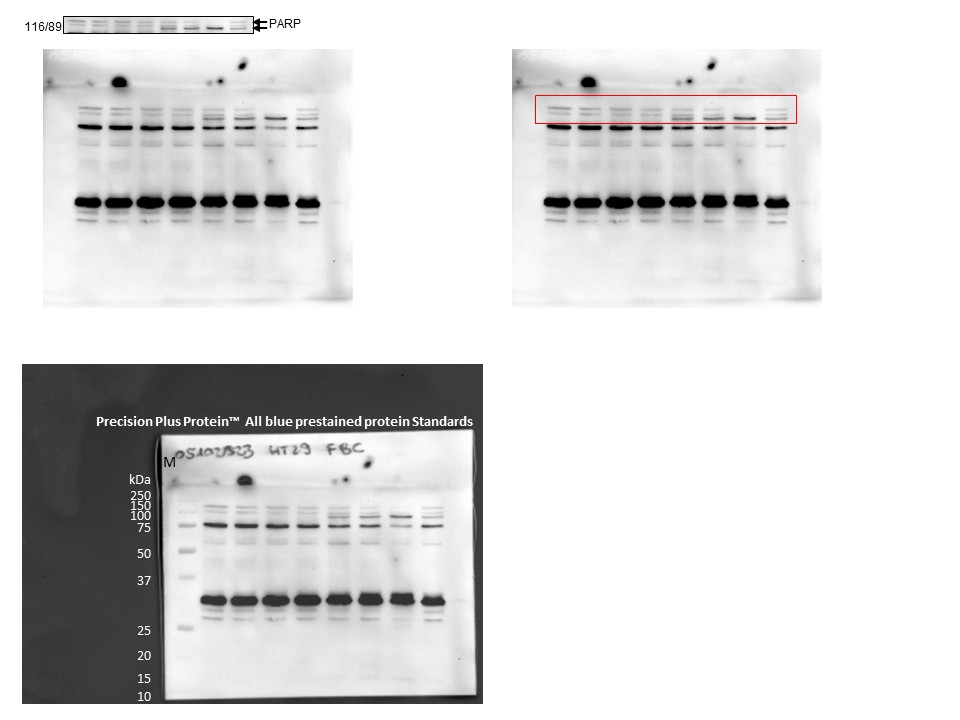

Supplement: Supplementary file 2 [file Presentation2.zip › marked-WB/HT29-rawdata-marked/Folie10.TIF]

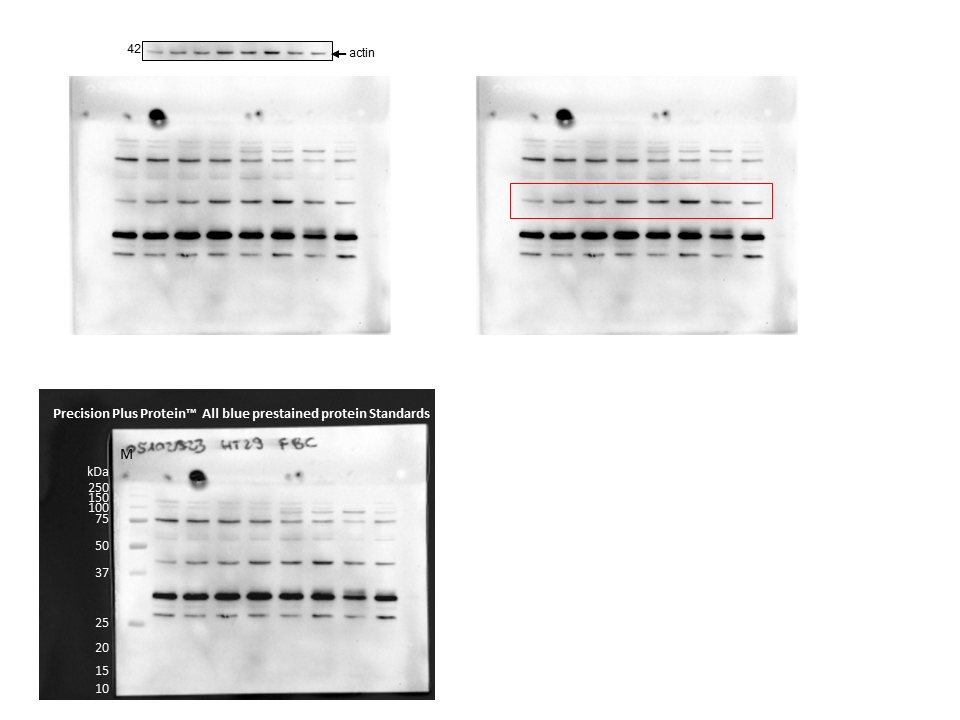

Supplement: Supplementary file 2 [file Presentation2.zip › marked-WB/HT29-rawdata-marked/Folie11.TIF]

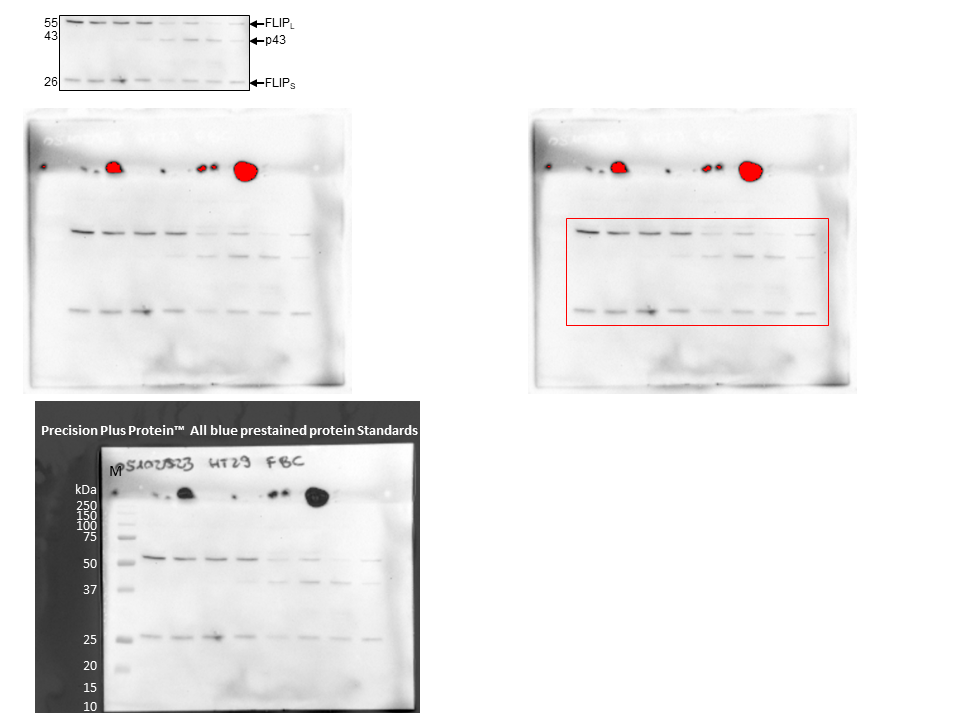

Supplement: Supplementary file 2 [file Presentation2.zip › marked-WB/HT29-rawdata-marked/Folie2.TIF]

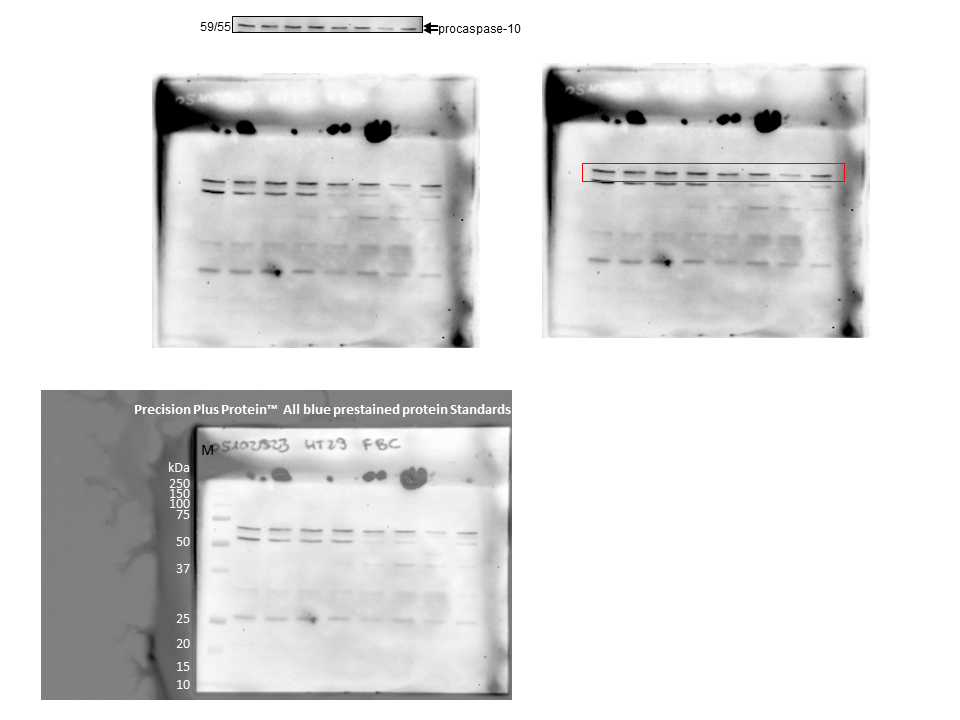

Supplement: Supplementary file 2 [file Presentation2.zip › marked-WB/HT29-rawdata-marked/Folie3.TIF]

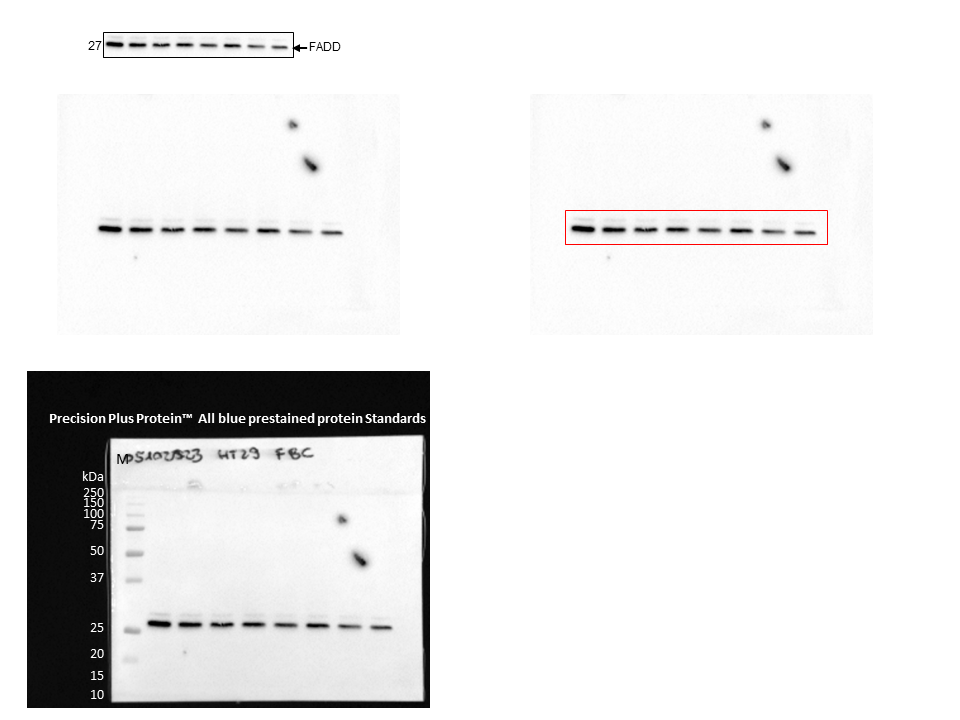

Supplement: Supplementary file 2 [file Presentation2.zip › marked-WB/HT29-rawdata-marked/Folie4.TIF]

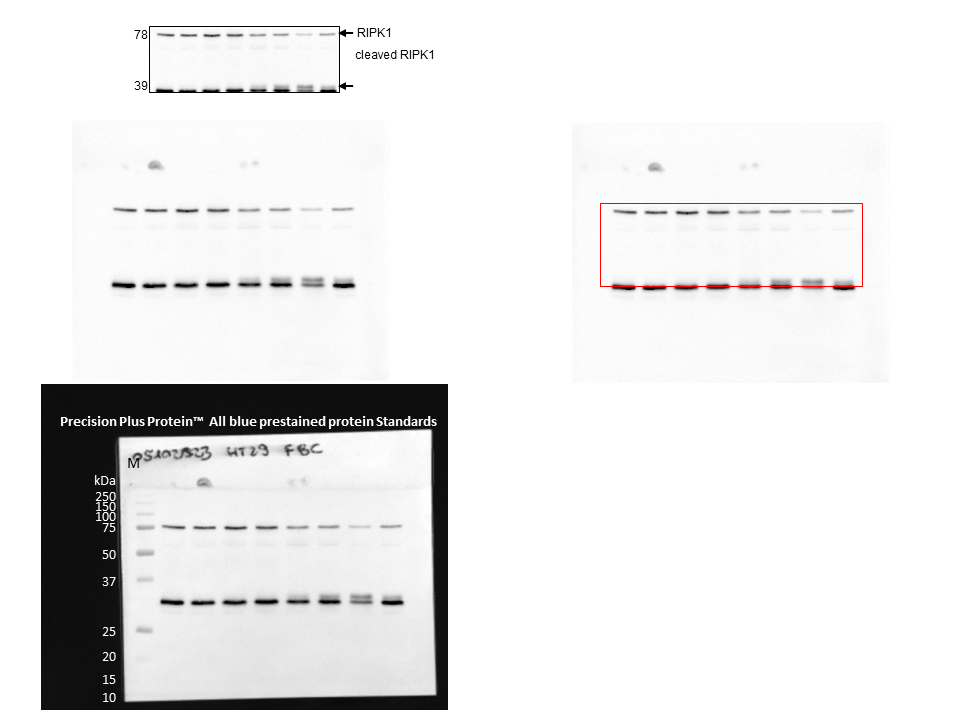

Supplement: Supplementary file 2 [file Presentation2.zip › marked-WB/HT29-rawdata-marked/Folie5.TIF]

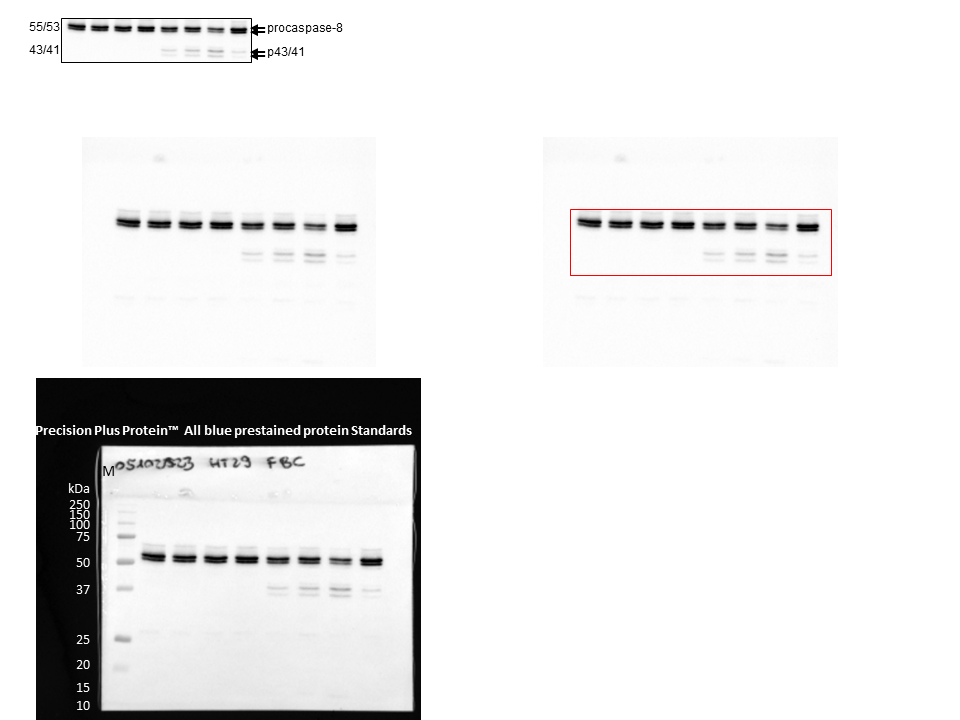

Supplement: Supplementary file 2 [file Presentation2.zip › marked-WB/HT29-rawdata-marked/Folie6.TIF]

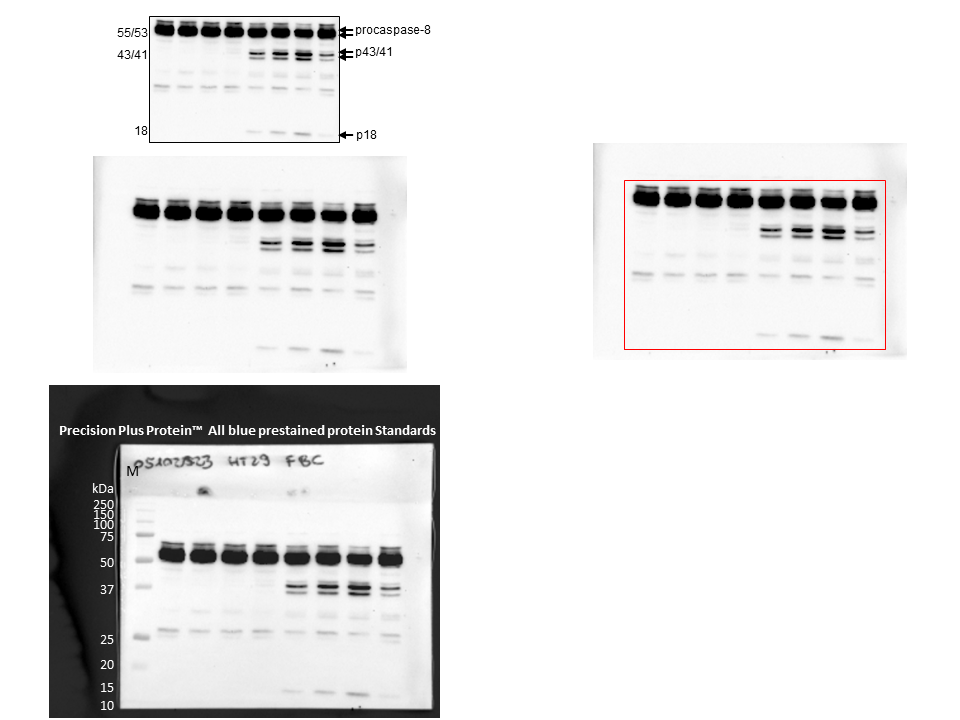

Supplement: Supplementary file 2 [file Presentation2.zip › marked-WB/HT29-rawdata-marked/Folie7.TIF]

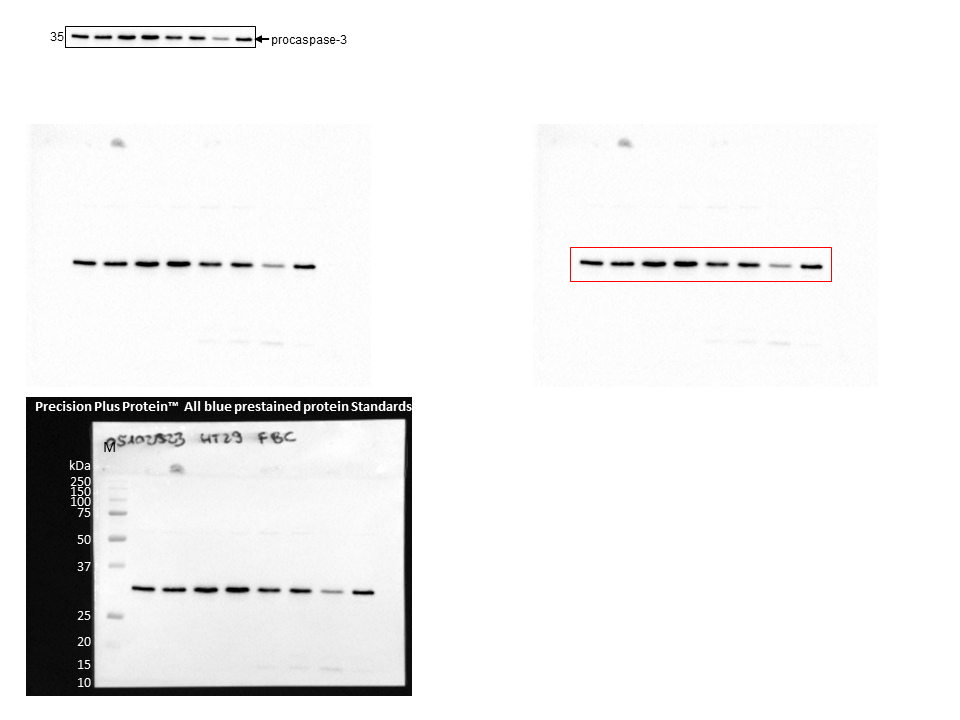

Supplement: Supplementary file 2 [file Presentation2.zip › marked-WB/HT29-rawdata-marked/Folie8.TIF]

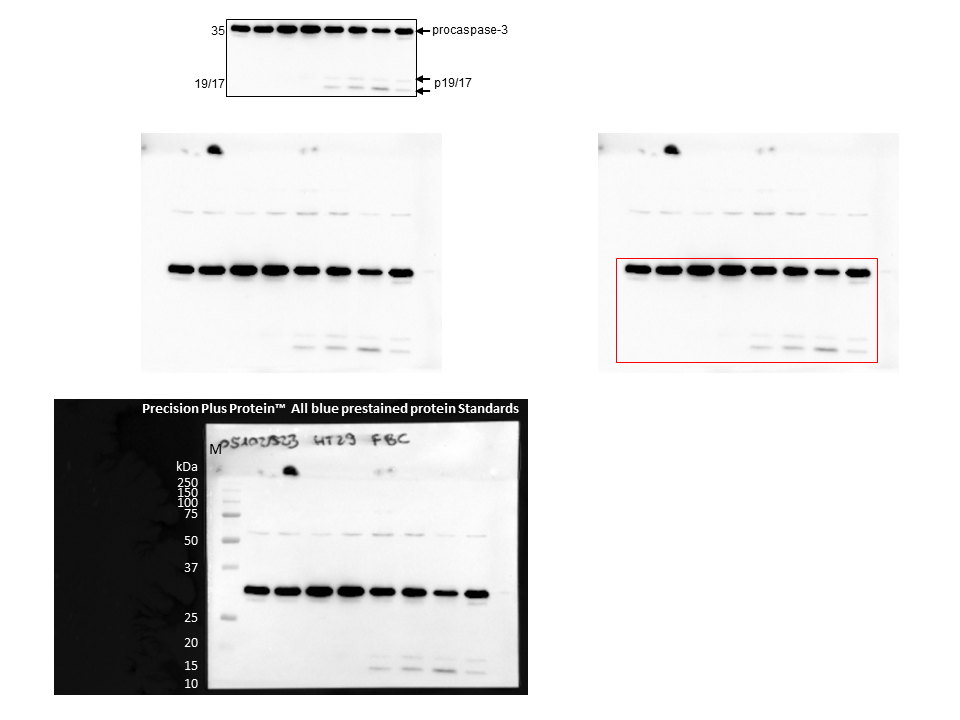

Supplement: Supplementary file 2 [file Presentation2.zip › marked-WB/HT29-rawdata-marked/Folie9.TIF]

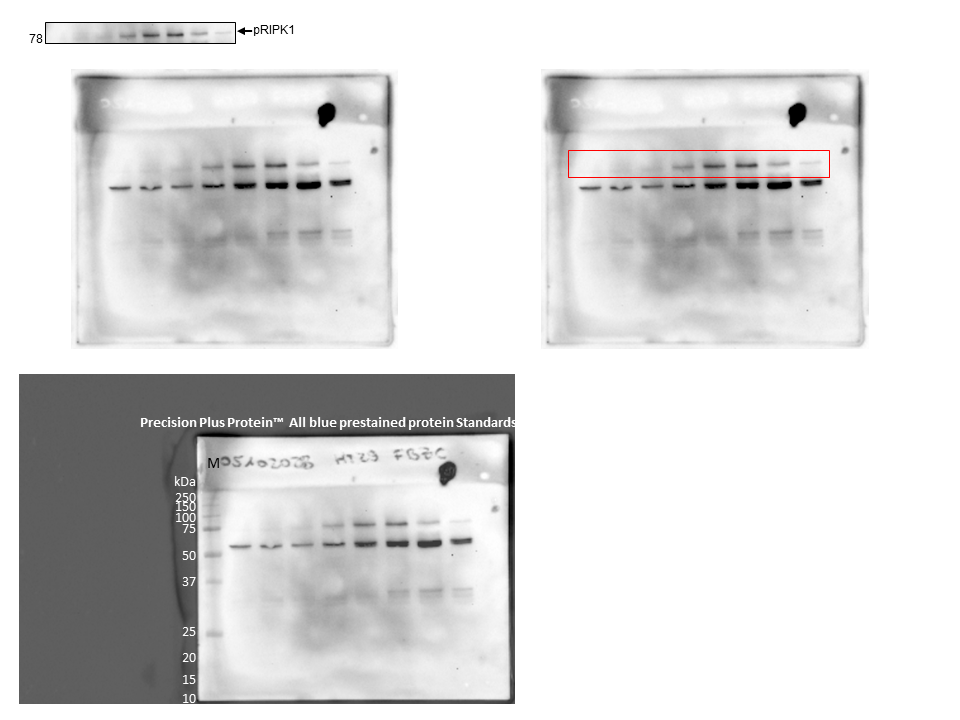

Supplement: Supplementary file 2 [file Presentation2.zip › marked-WB/rawdata-HT29-zvad-marked/Folie2.TIF]

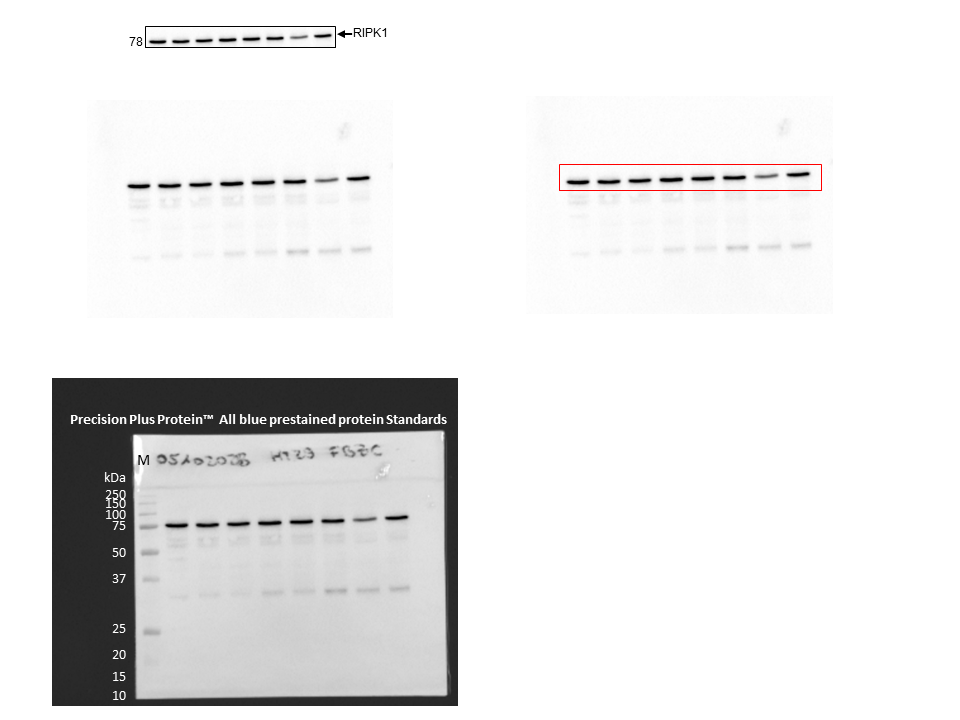

Supplement: Supplementary file 2 [file Presentation2.zip › marked-WB/rawdata-HT29-zvad-marked/Folie3.TIF]

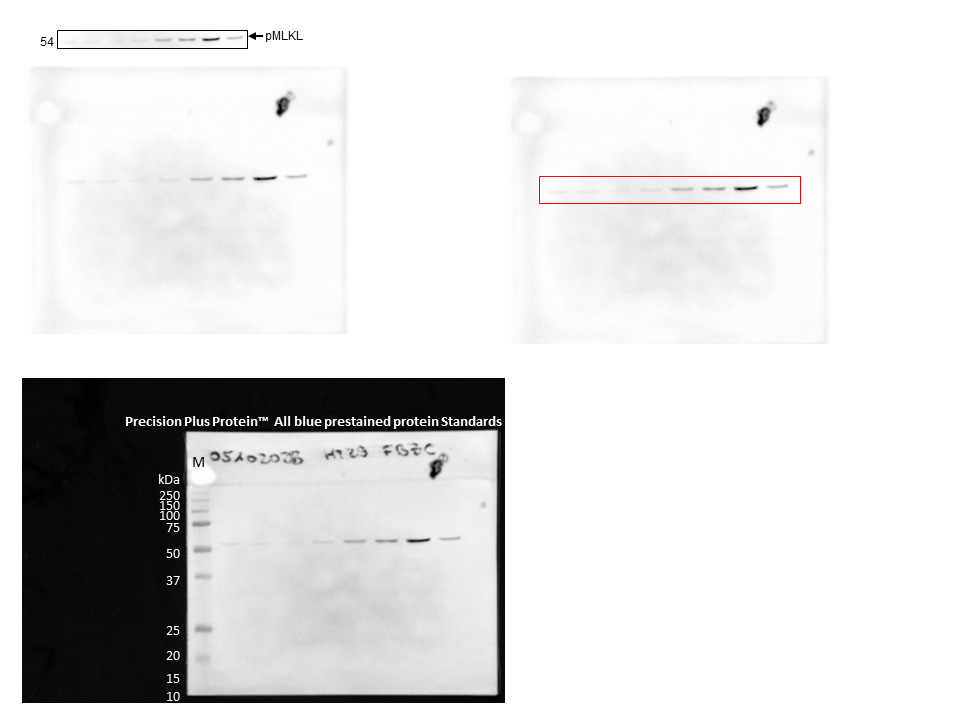

Supplement: Supplementary file 2 [file Presentation2.zip › marked-WB/rawdata-HT29-zvad-marked/Folie4.TIF]

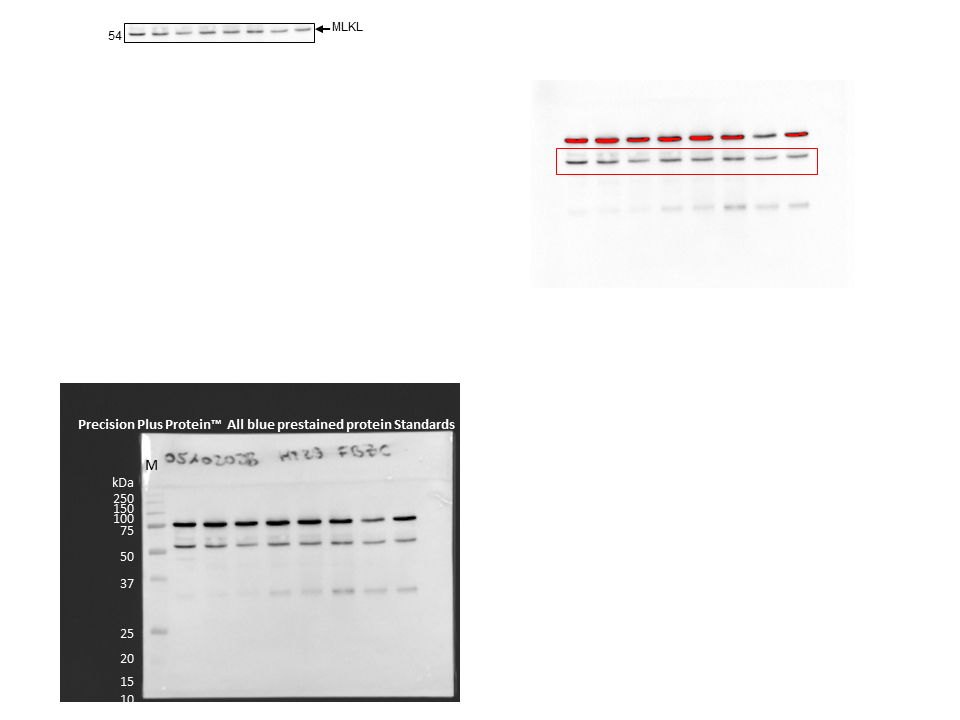

Supplement: Supplementary file 2 [file Presentation2.zip › marked-WB/rawdata-HT29-zvad-marked/Folie5.TIF]

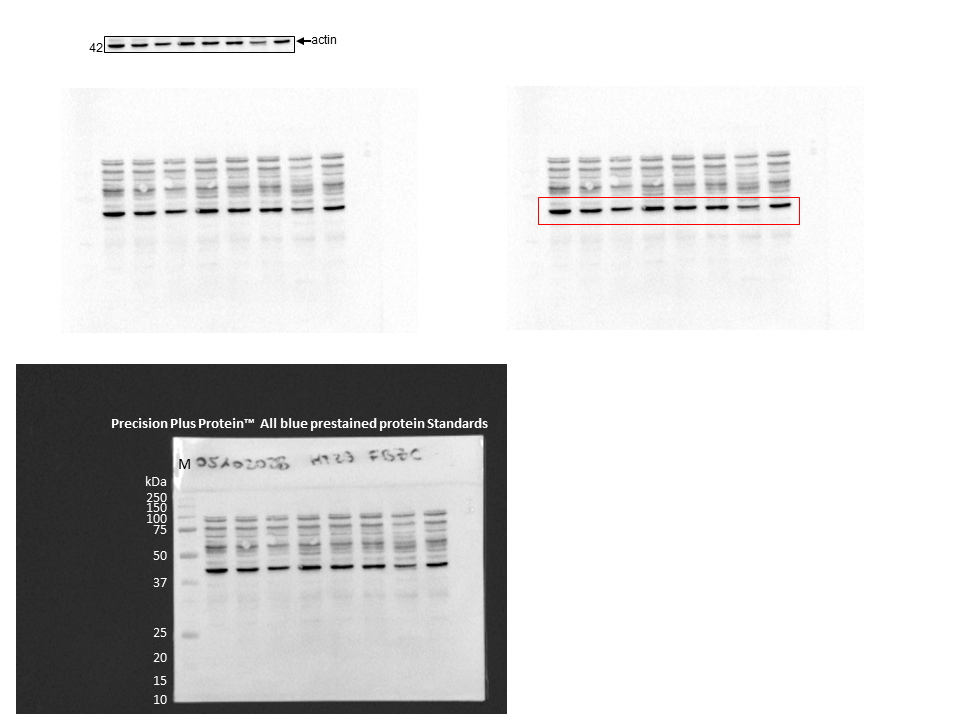

Supplement: Supplementary file 2 [file Presentation2.zip › marked-WB/rawdata-HT29-zvad-marked/Folie6.TIF]

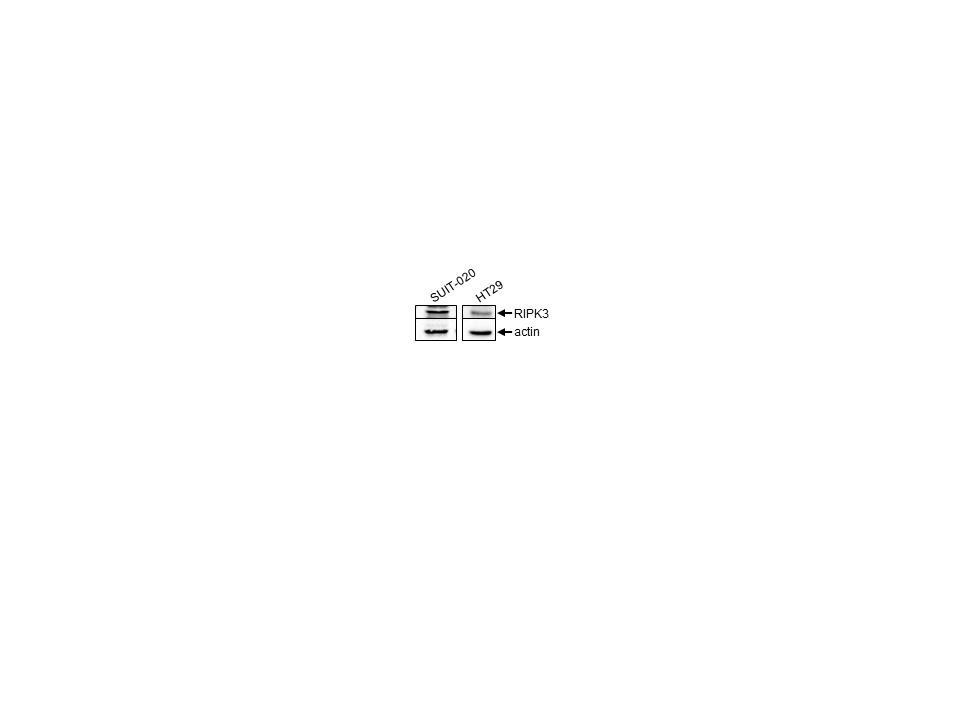

Supplement: Supplementary file 2 [file Presentation2.zip › marked-WB/RIPK3blot-rawdata-marked/Folie1.TIF]

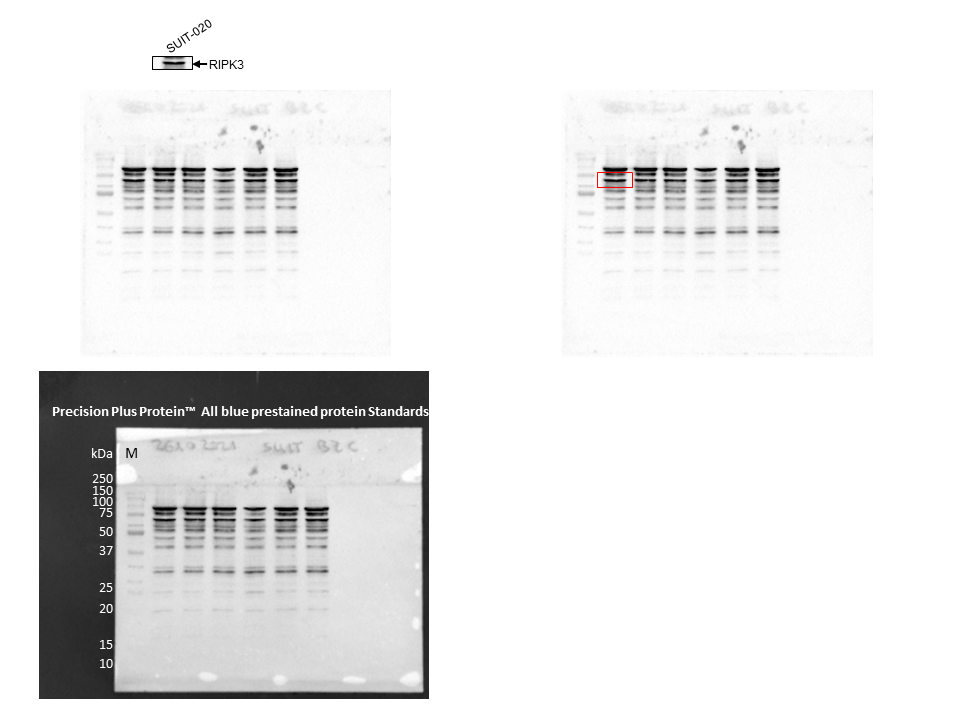

Supplement: Supplementary file 2 [file Presentation2.zip › marked-WB/RIPK3blot-rawdata-marked/Folie2.TIF]

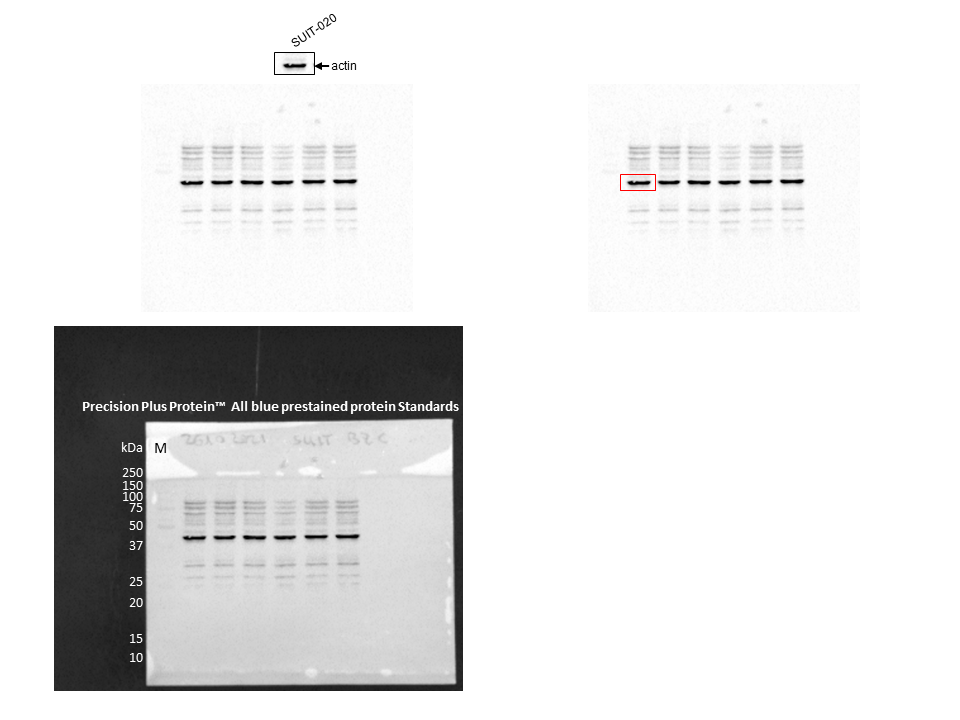

Supplement: Supplementary file 2 [file Presentation2.zip › marked-WB/RIPK3blot-rawdata-marked/Folie3.TIF]

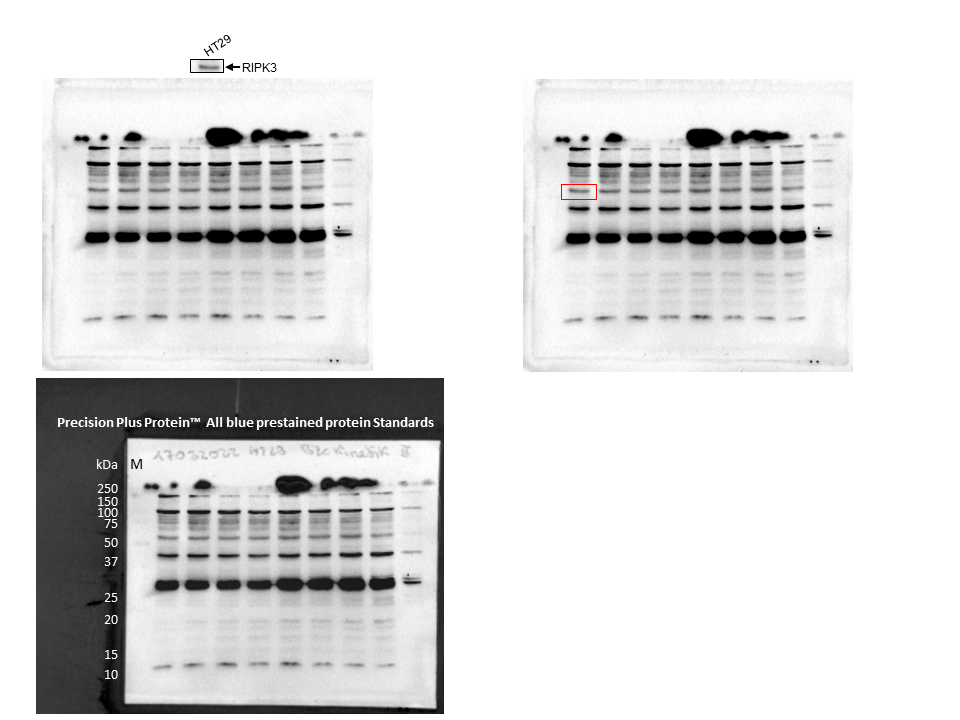

Supplement: Supplementary file 2 [file Presentation2.zip › marked-WB/RIPK3blot-rawdata-marked/Folie4.TIF]

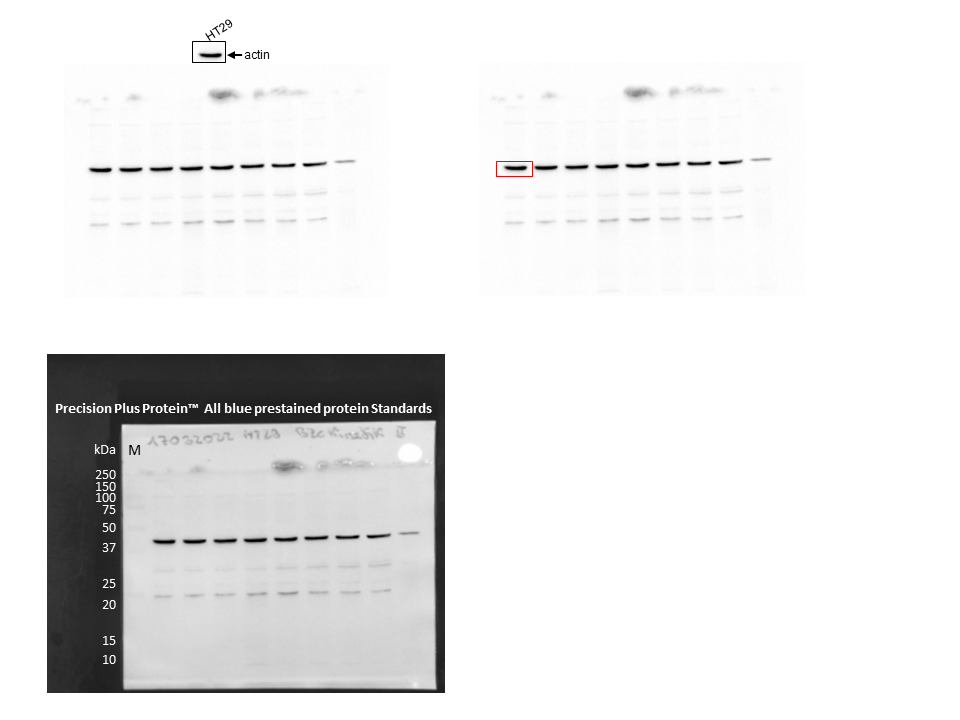

Supplement: Supplementary file 2 [file Presentation2.zip › marked-WB/RIPK3blot-rawdata-marked/Folie5.TIF]

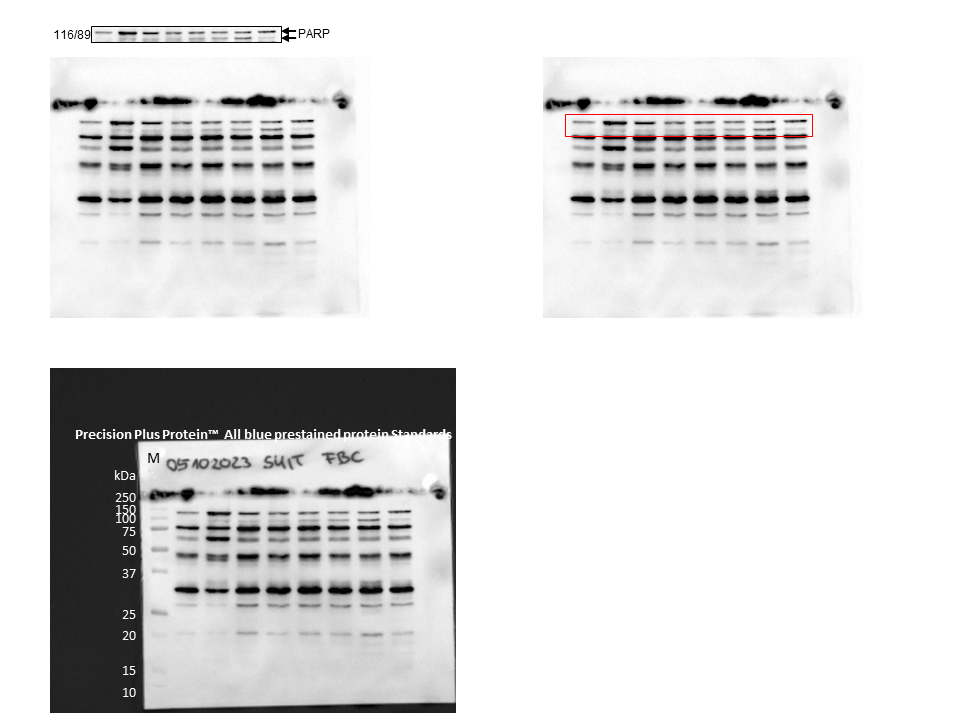

Supplement: Supplementary file 2 [file Presentation2.zip › marked-WB/suit-rawdata-marked/Folie10.TIF]

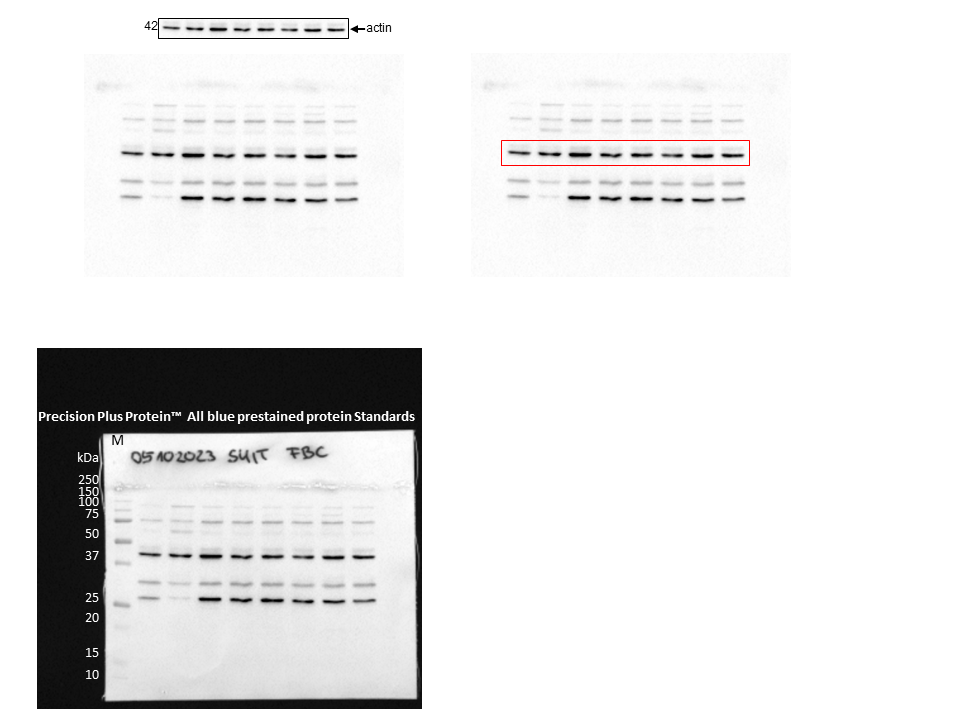

Supplement: Supplementary file 2 [file Presentation2.zip › marked-WB/suit-rawdata-marked/Folie11.TIF]

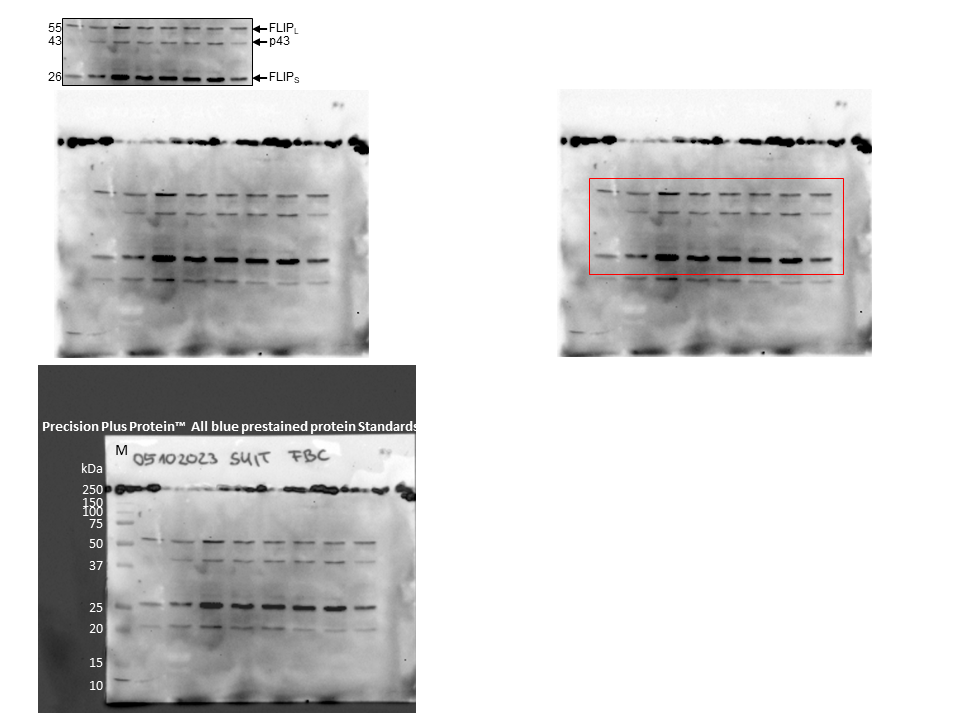

Supplement: Supplementary file 2 [file Presentation2.zip › marked-WB/suit-rawdata-marked/Folie2.TIF]

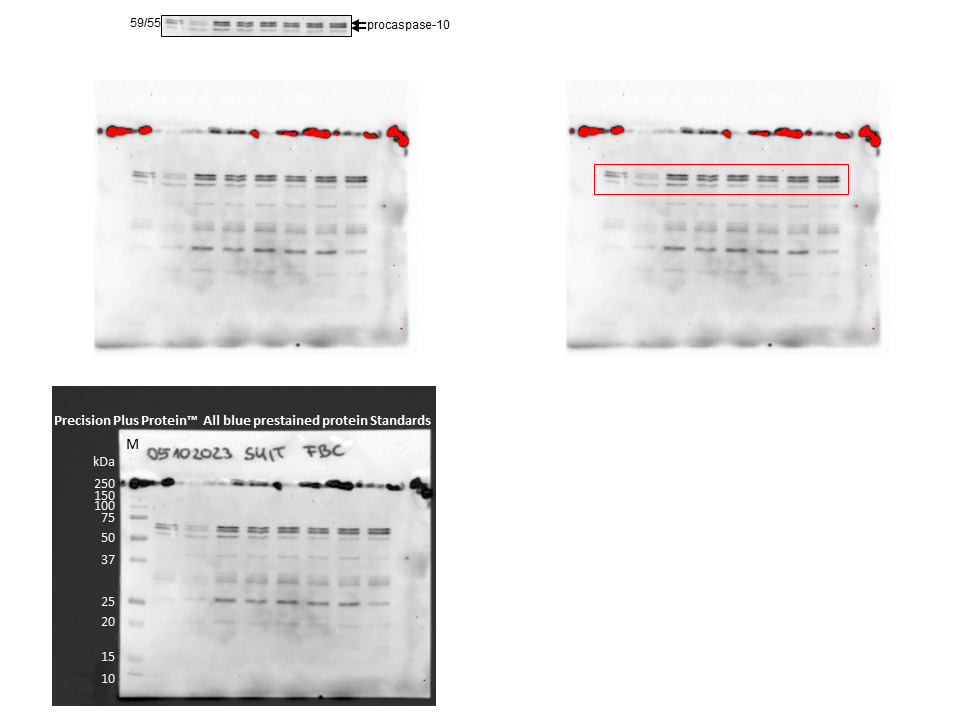

Supplement: Supplementary file 2 [file Presentation2.zip › marked-WB/suit-rawdata-marked/Folie3.TIF]

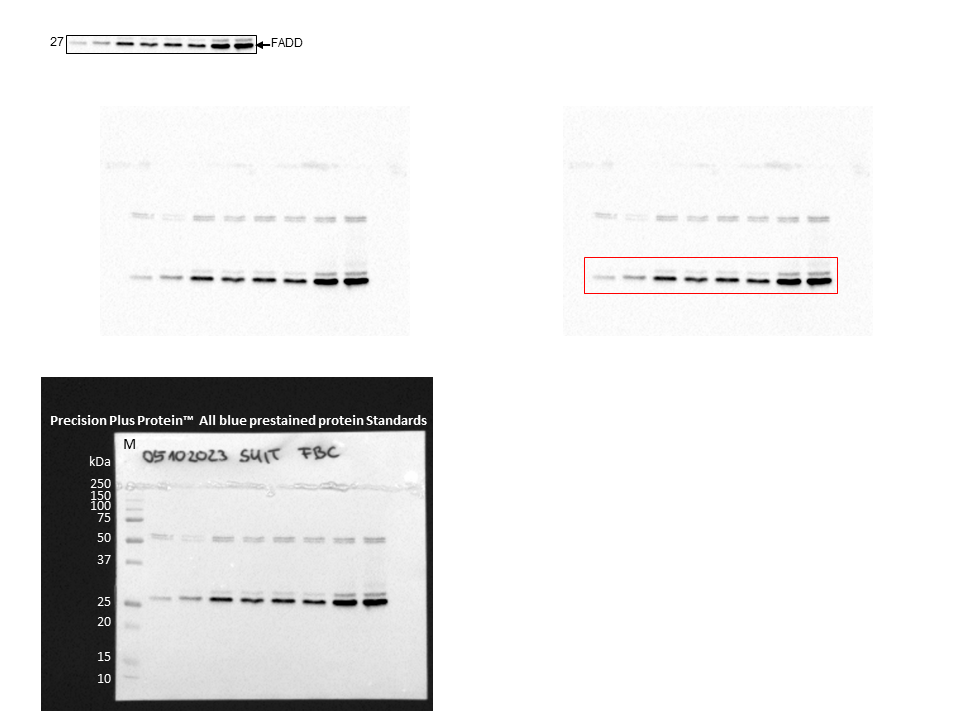

Supplement: Supplementary file 2 [file Presentation2.zip › marked-WB/suit-rawdata-marked/Folie4.TIF]

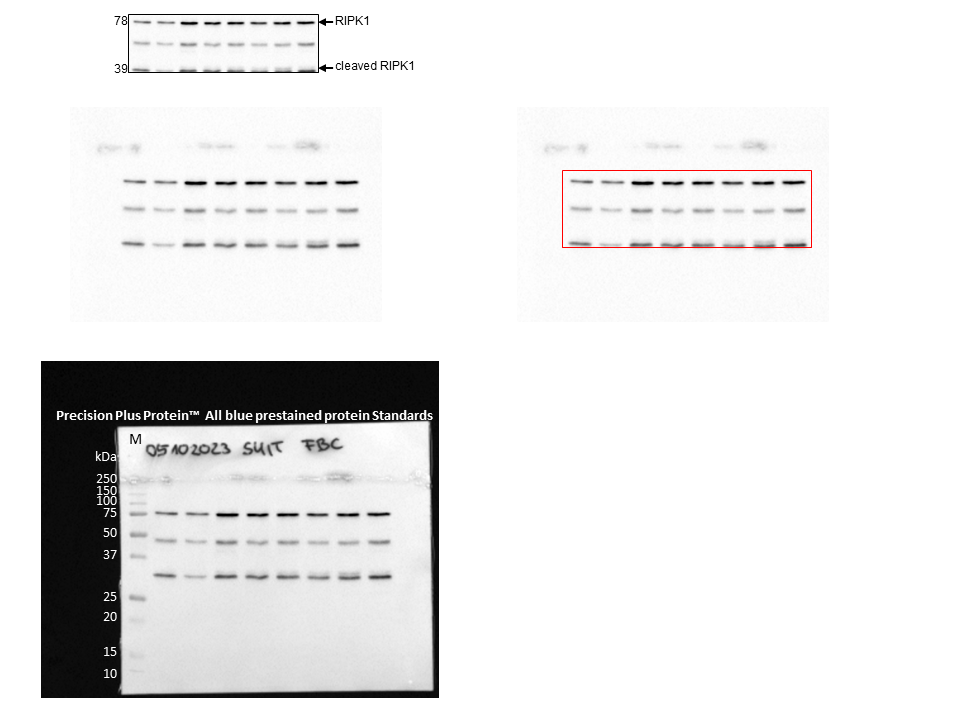

Supplement: Supplementary file 2 [file Presentation2.zip › marked-WB/suit-rawdata-marked/Folie5.TIF]

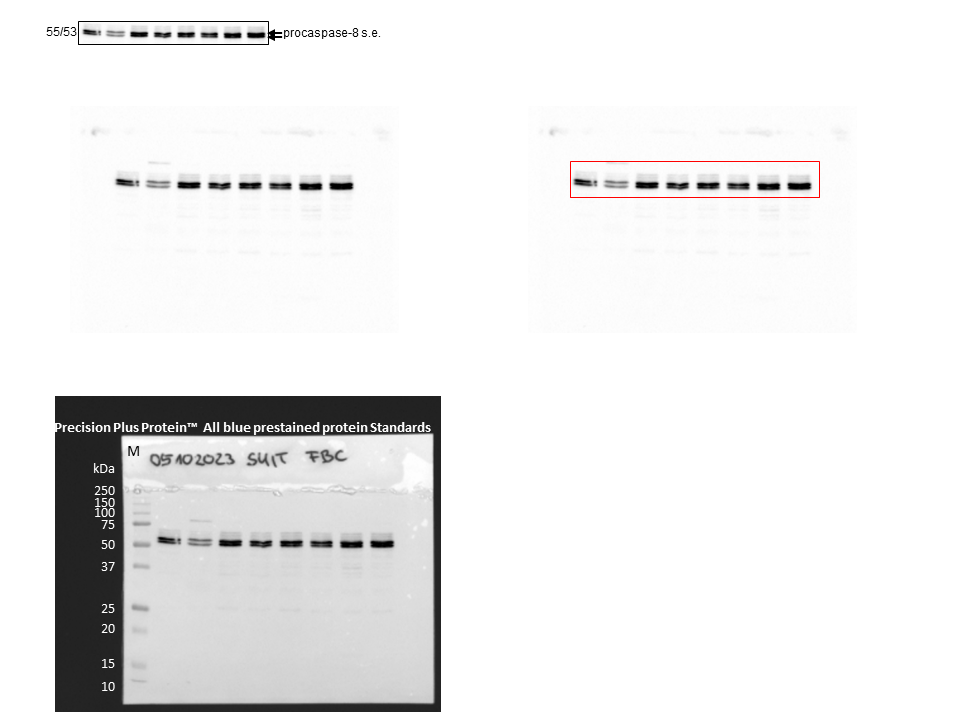

Supplement: Supplementary file 2 [file Presentation2.zip › marked-WB/suit-rawdata-marked/Folie6.TIF]

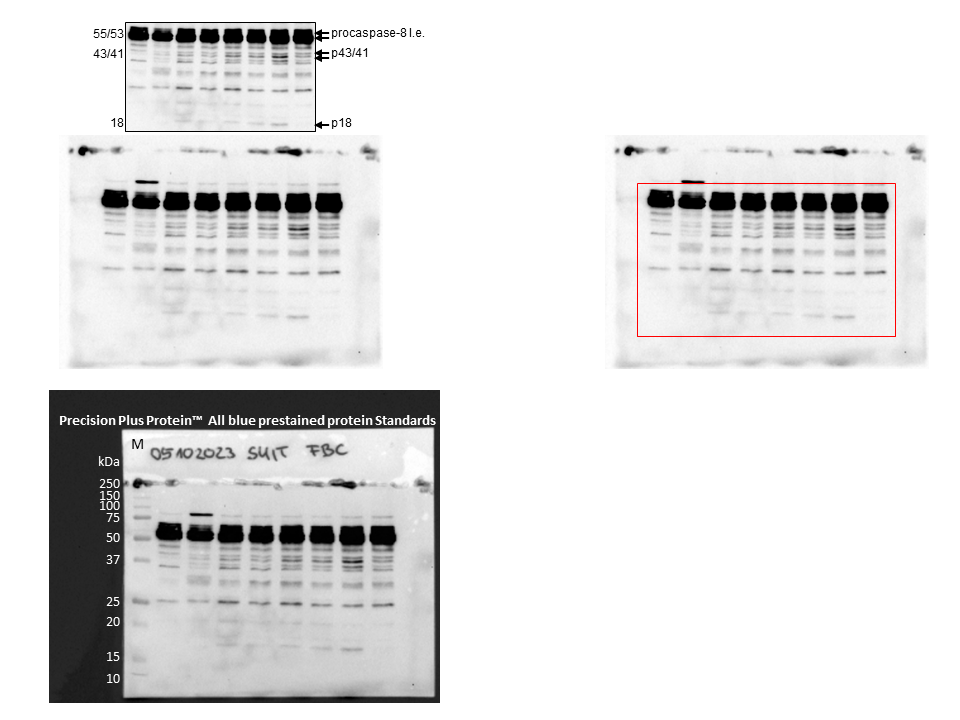

Supplement: Supplementary file 2 [file Presentation2.zip › marked-WB/suit-rawdata-marked/Folie7.TIF]

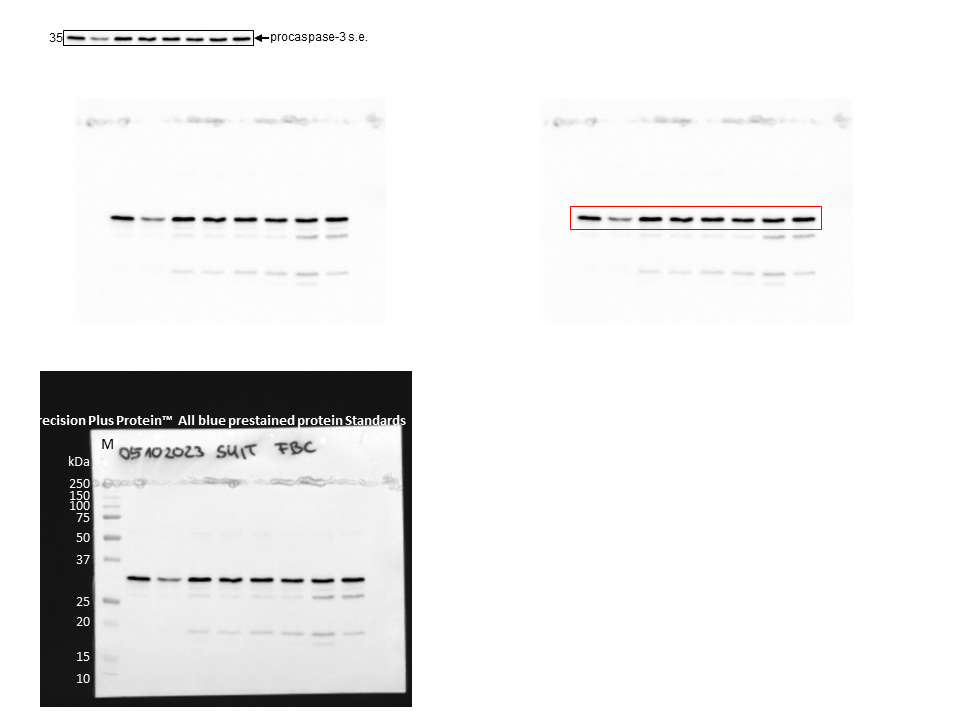

Supplement: Supplementary file 2 [file Presentation2.zip › marked-WB/suit-rawdata-marked/Folie8.TIF]

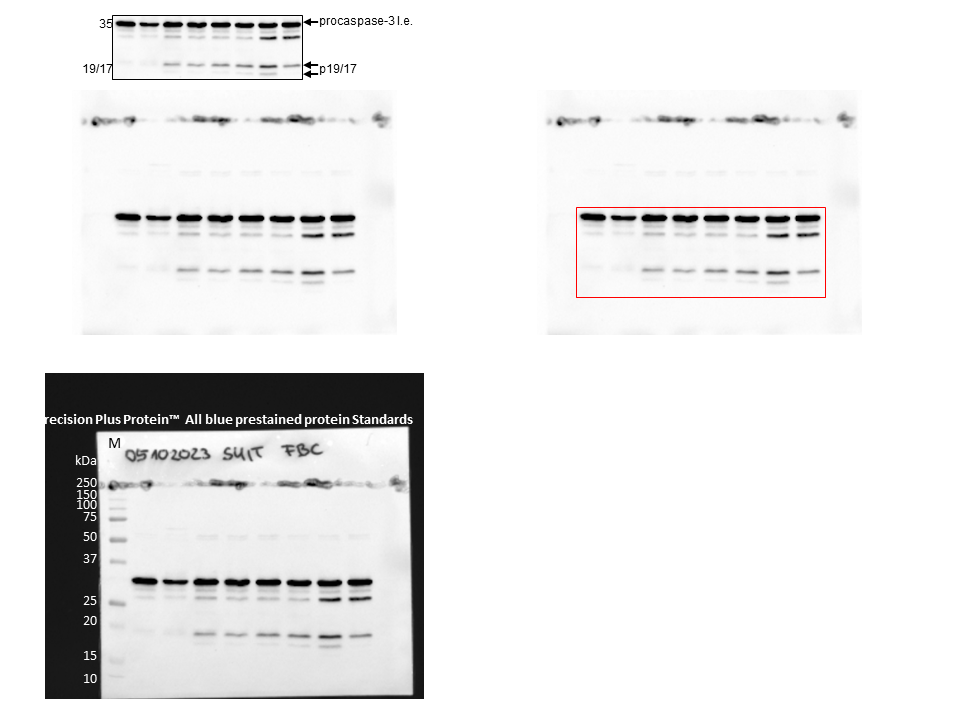

Supplement: Supplementary file 2 [file Presentation2.zip › marked-WB/suit-rawdata-marked/Folie9.TIF]

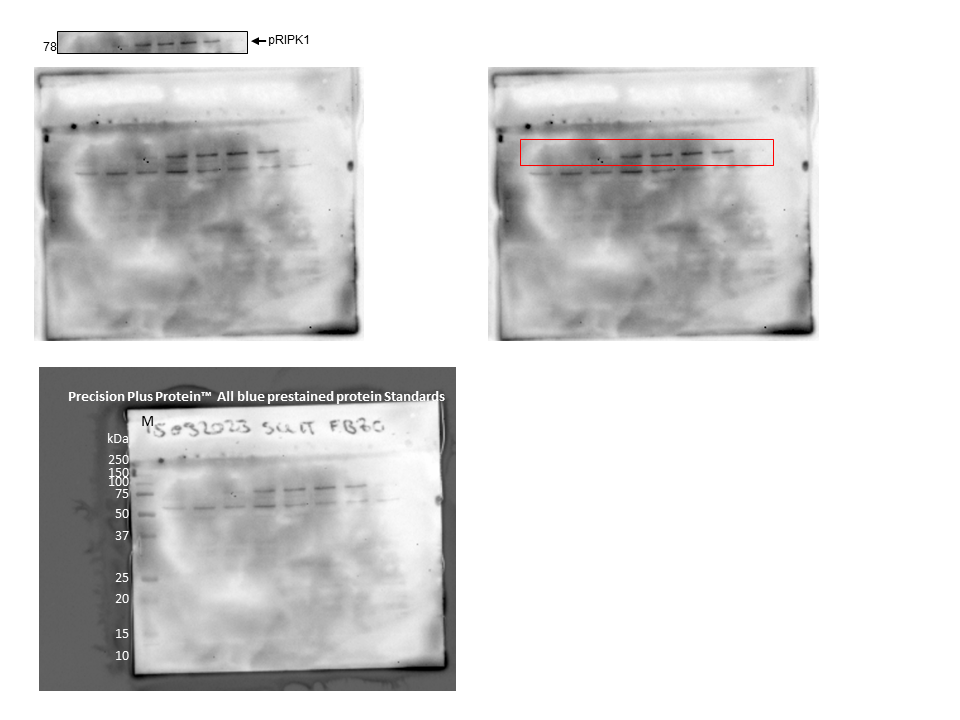

Supplement: Supplementary file 2 [file Presentation2.zip › marked-WB/SUIT-zVAD-marked/Folie2.TIF]

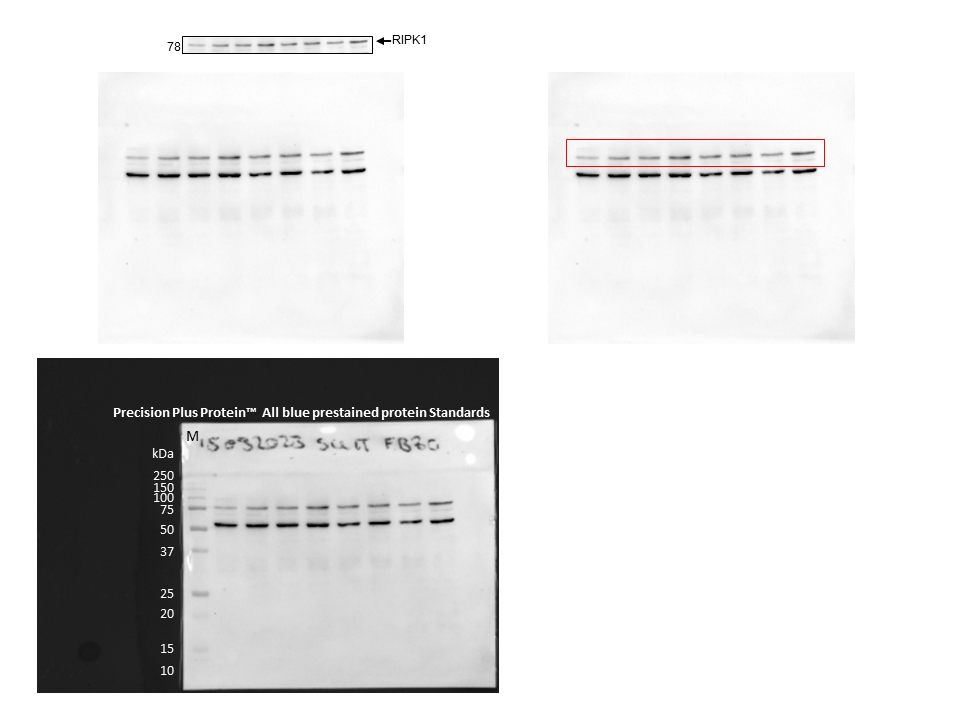

Supplement: Supplementary file 2 [file Presentation2.zip › marked-WB/SUIT-zVAD-marked/Folie3.TIF]

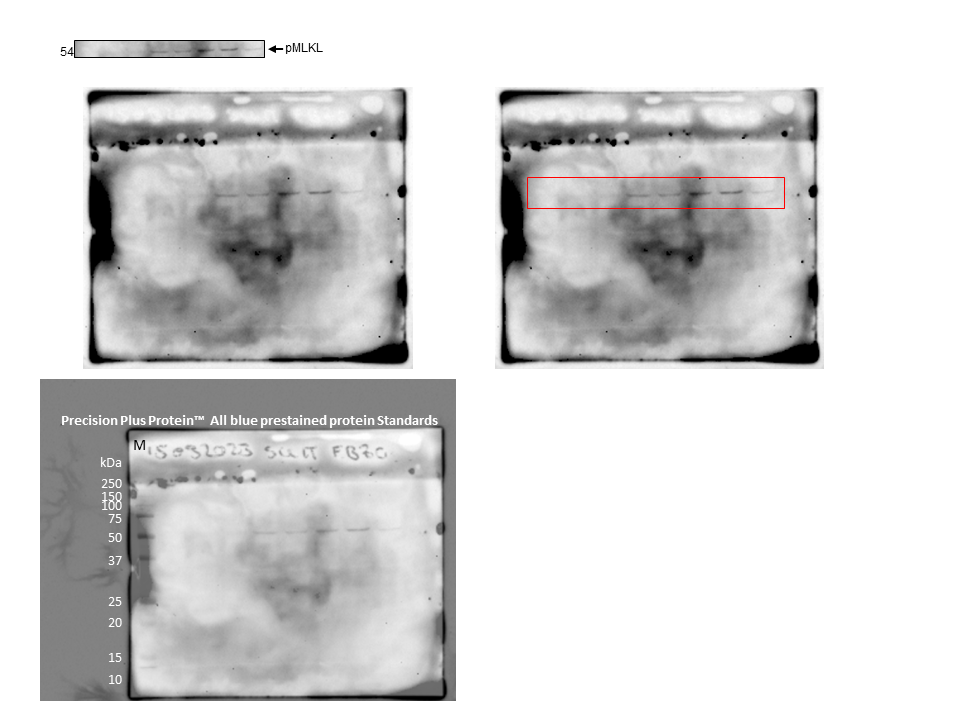

Supplement: Supplementary file 2 [file Presentation2.zip › marked-WB/SUIT-zVAD-marked/Folie4.TIF]

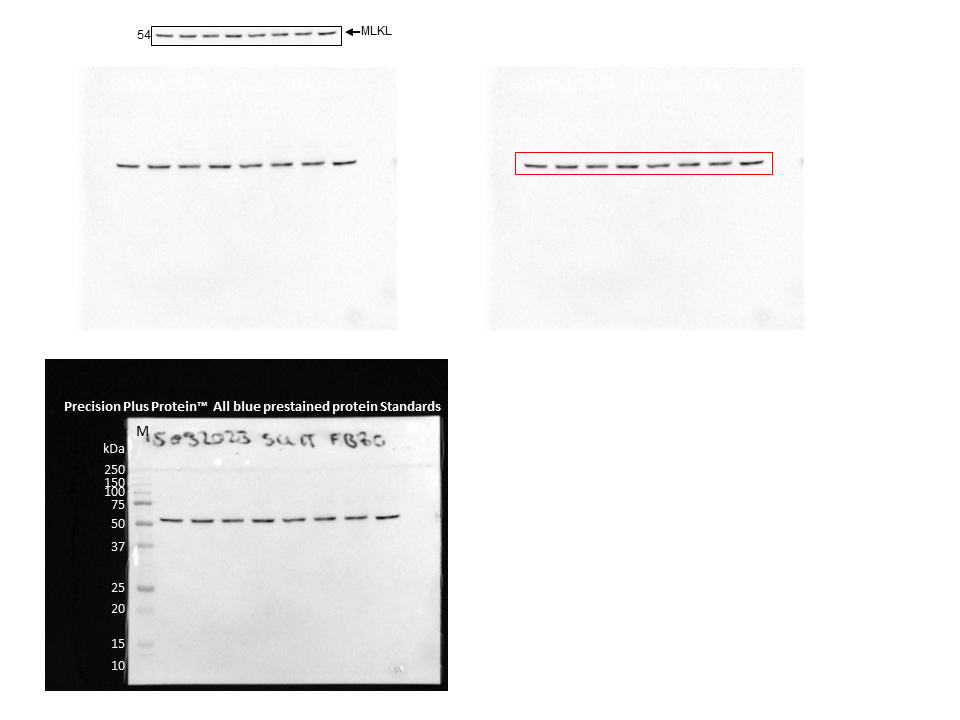

Supplement: Supplementary file 2 [file Presentation2.zip › marked-WB/SUIT-zVAD-marked/Folie5.TIF]

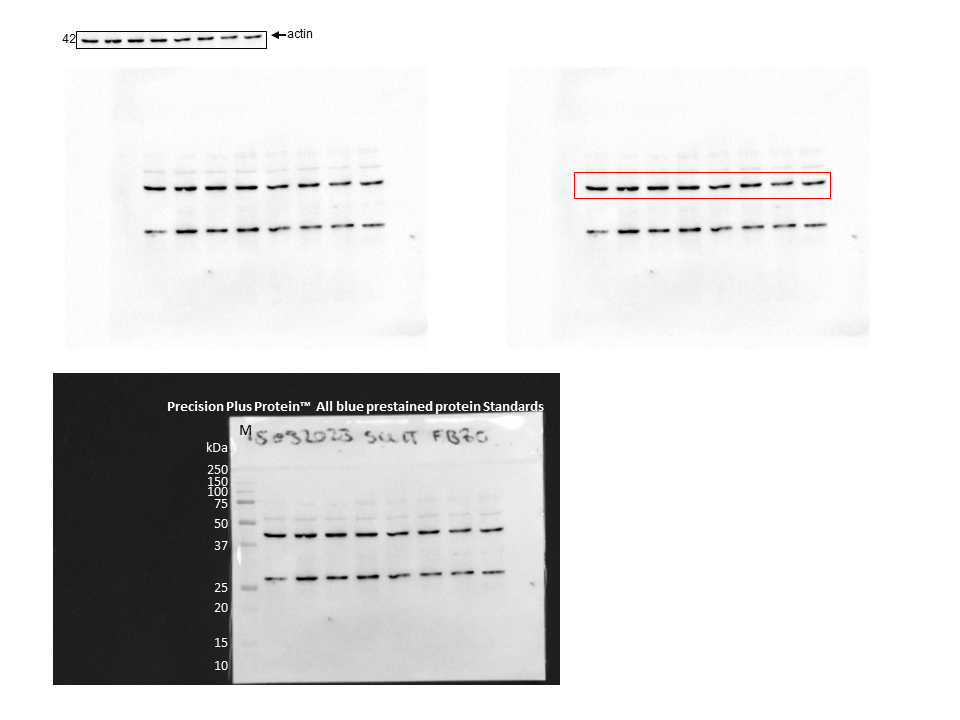

Supplement: Supplementary file 2 [file Presentation2.zip › marked-WB/SUIT-zVAD-marked/Folie6.TIF]

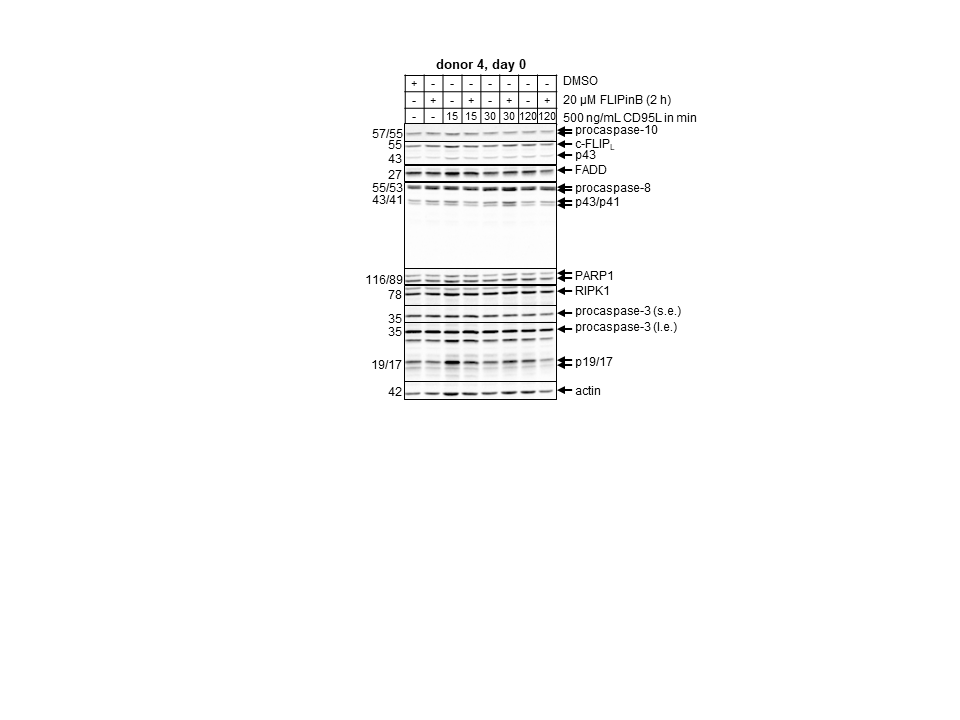

Supplement: Supplementary file 2 [file Presentation2.zip › marked-WB/Tcells-rawdata-marked/Folie1.TIF]

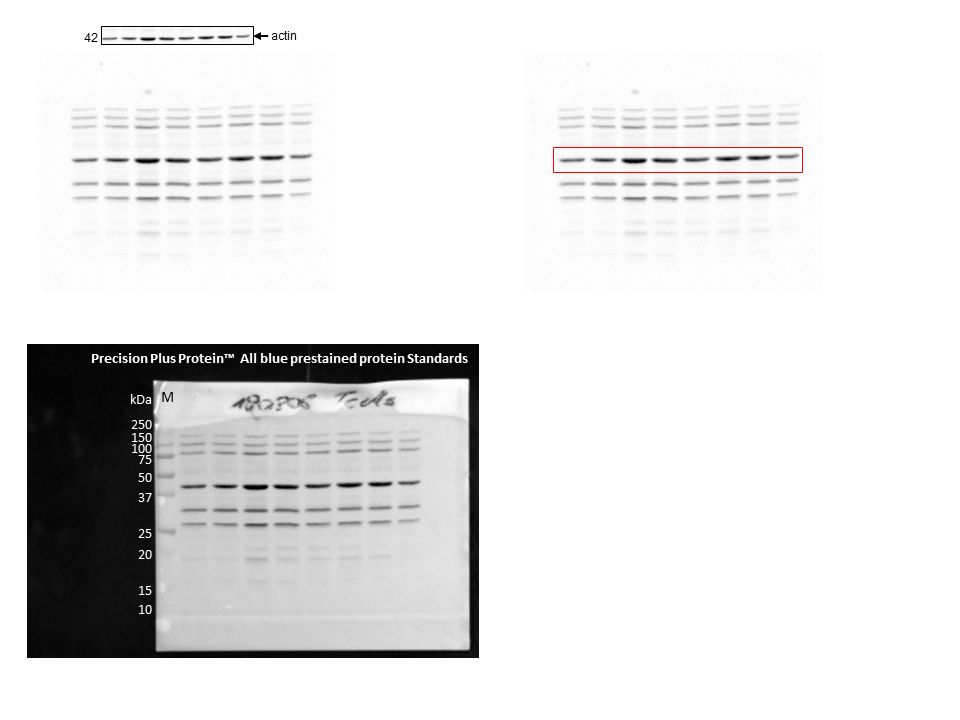

Supplement: Supplementary file 2 [file Presentation2.zip › marked-WB/Tcells-rawdata-marked/Folie10.TIF]

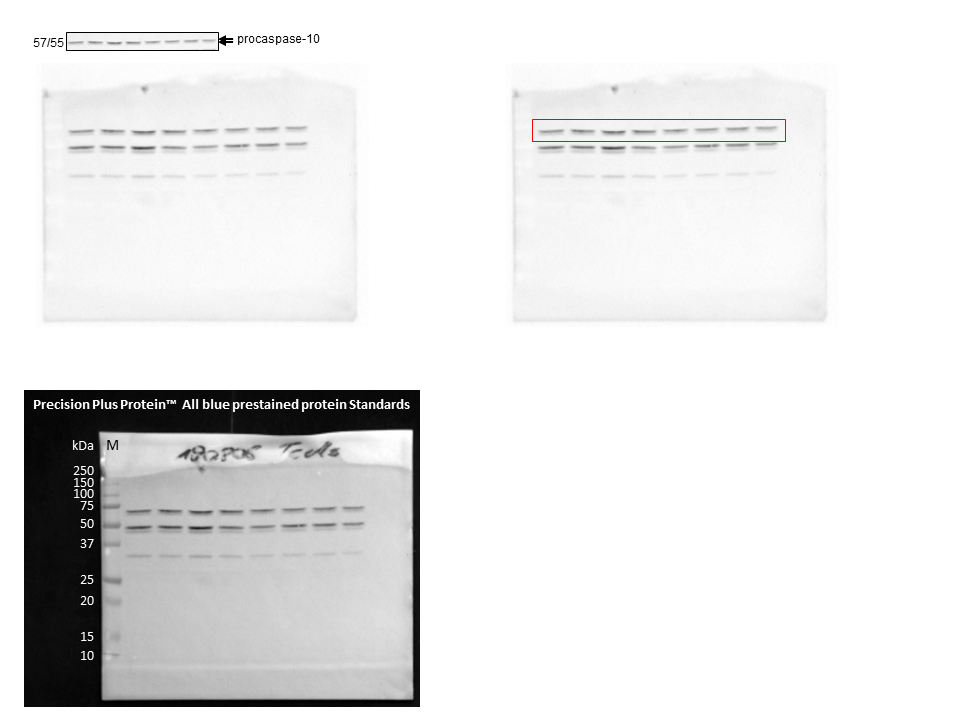

Supplement: Supplementary file 2 [file Presentation2.zip › marked-WB/Tcells-rawdata-marked/Folie2.TIF]

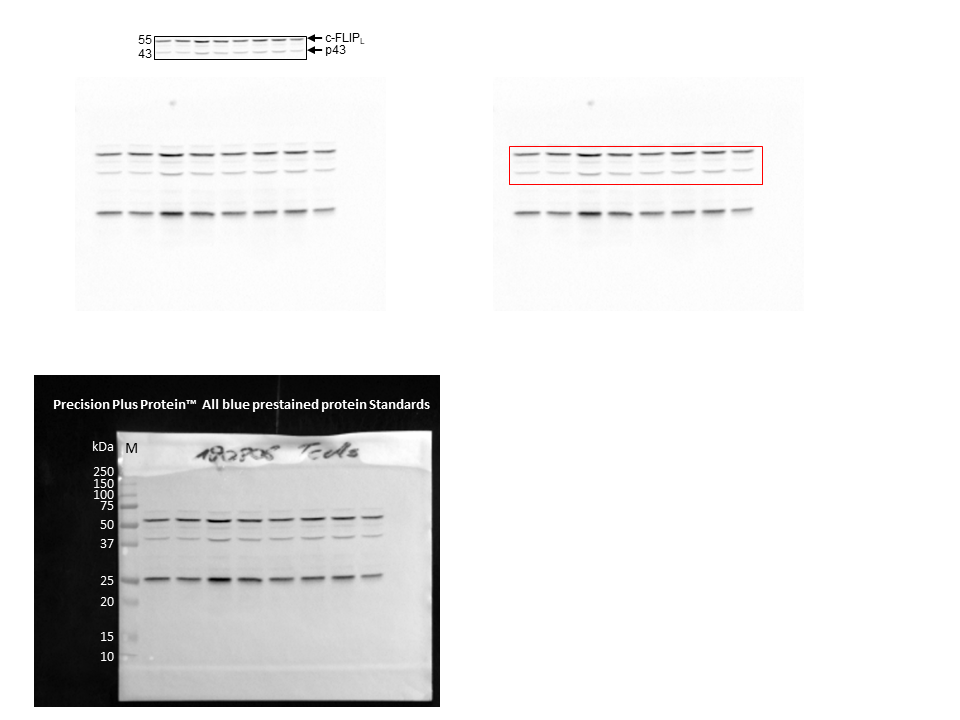

Supplement: Supplementary file 2 [file Presentation2.zip › marked-WB/Tcells-rawdata-marked/Folie3.TIF]

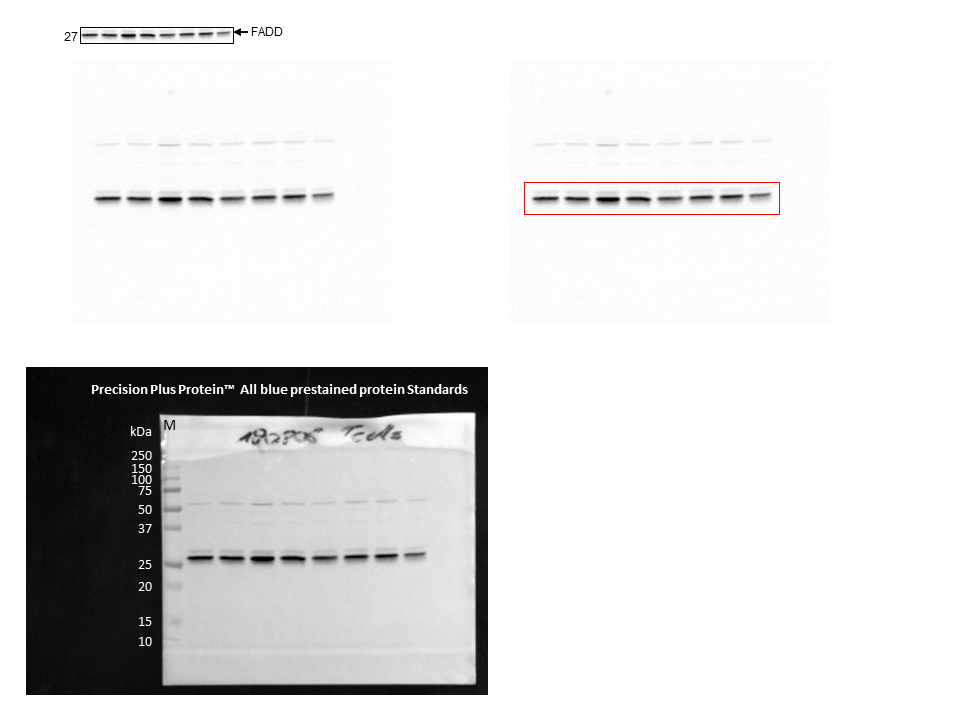

Supplement: Supplementary file 2 [file Presentation2.zip › marked-WB/Tcells-rawdata-marked/Folie4.TIF]

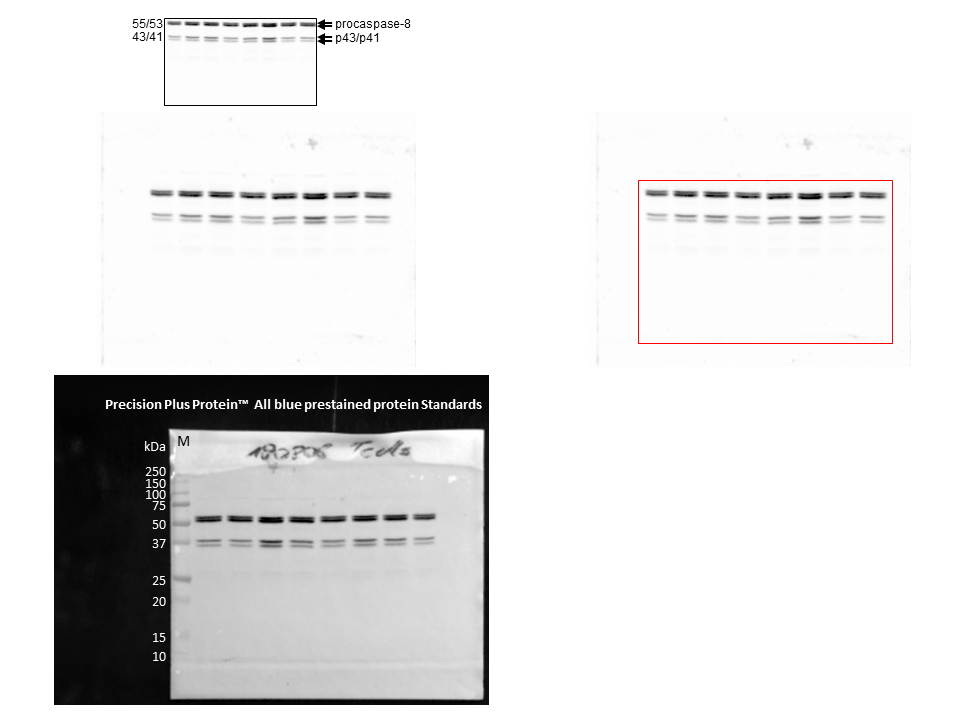

Supplement: Supplementary file 2 [file Presentation2.zip › marked-WB/Tcells-rawdata-marked/Folie5.TIF]

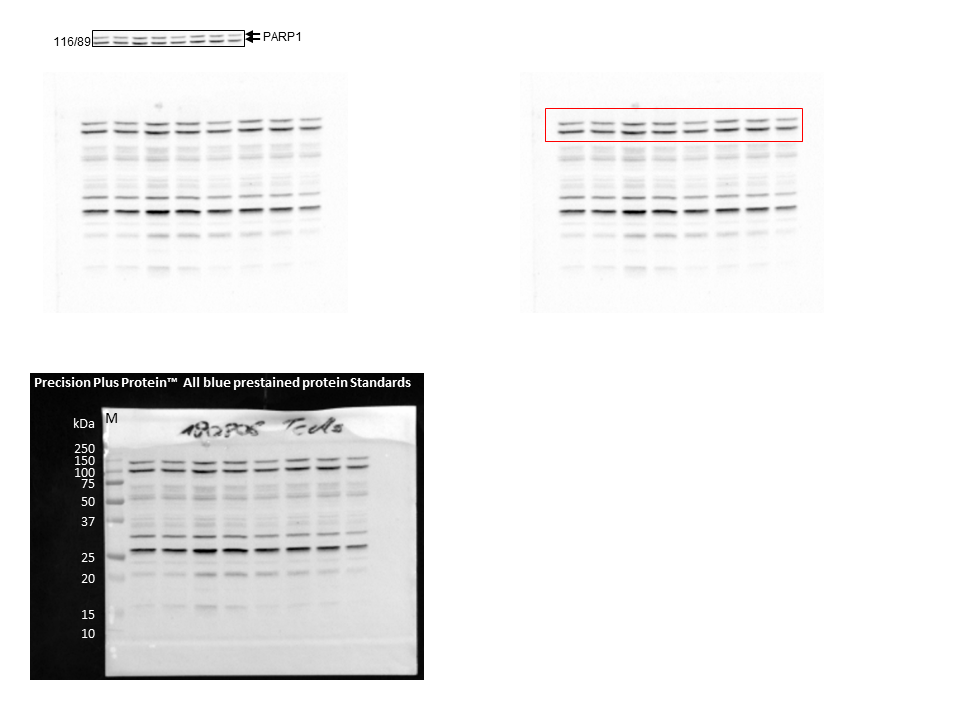

Supplement: Supplementary file 2 [file Presentation2.zip › marked-WB/Tcells-rawdata-marked/Folie6.TIF]

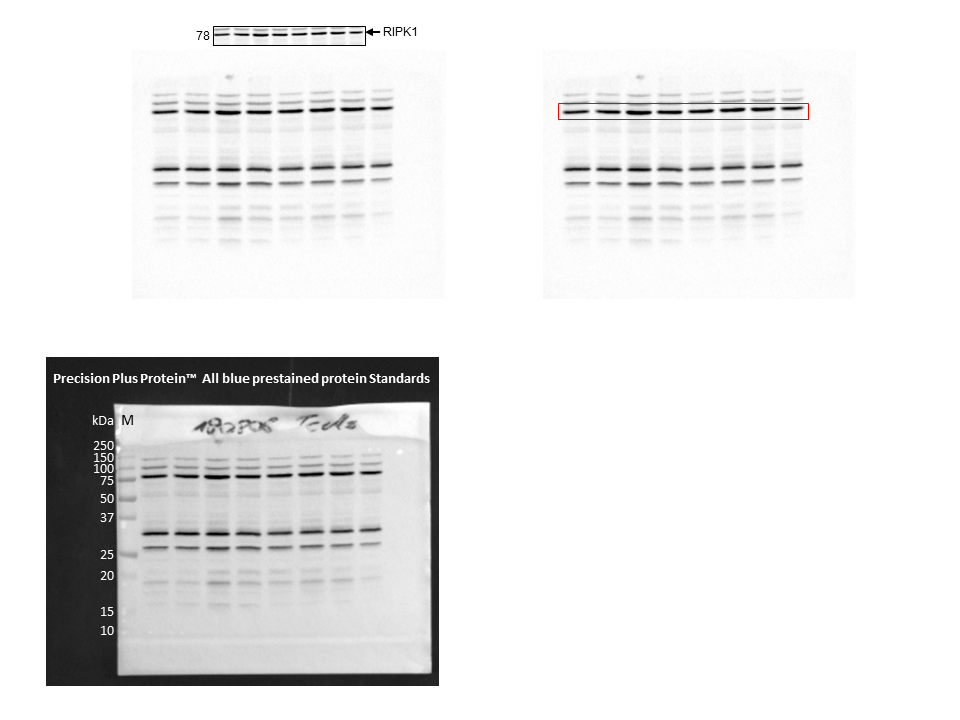

Supplement: Supplementary file 2 [file Presentation2.zip › marked-WB/Tcells-rawdata-marked/Folie7.TIF]

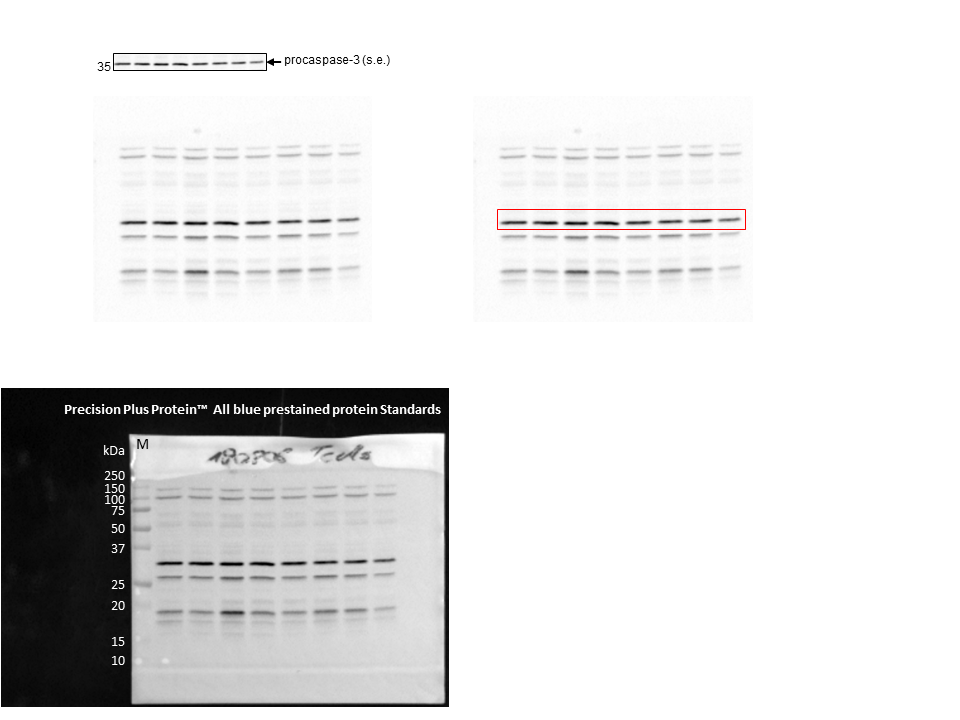

Supplement: Supplementary file 2 [file Presentation2.zip › marked-WB/Tcells-rawdata-marked/Folie8.TIF]

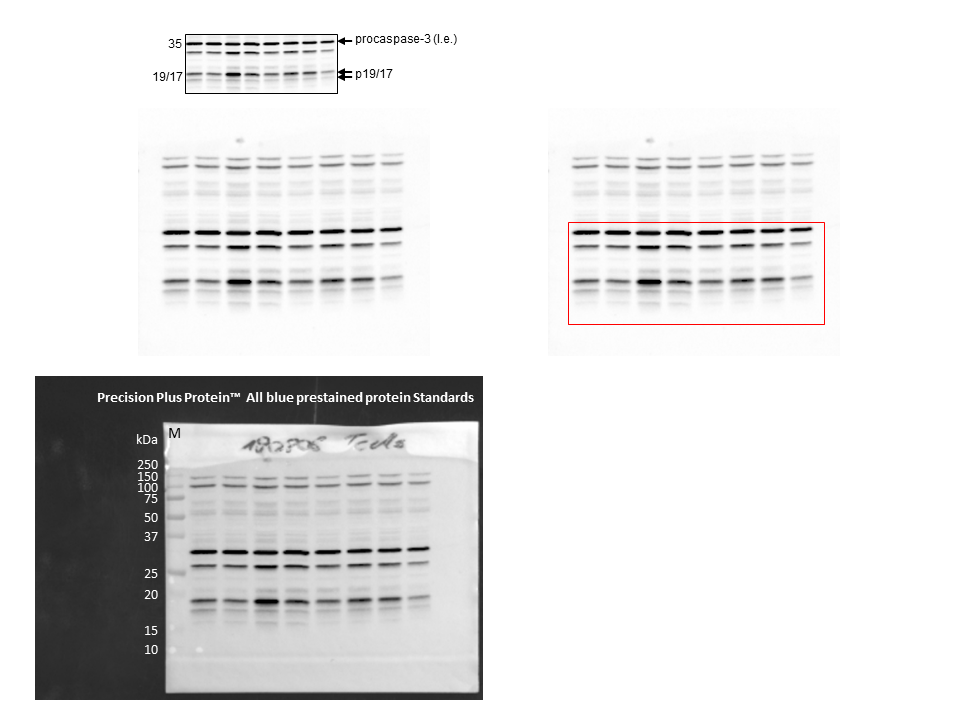

Supplement: Supplementary file 2 [file Presentation2.zip › marked-WB/Tcells-rawdata-marked/Folie9.TIF]

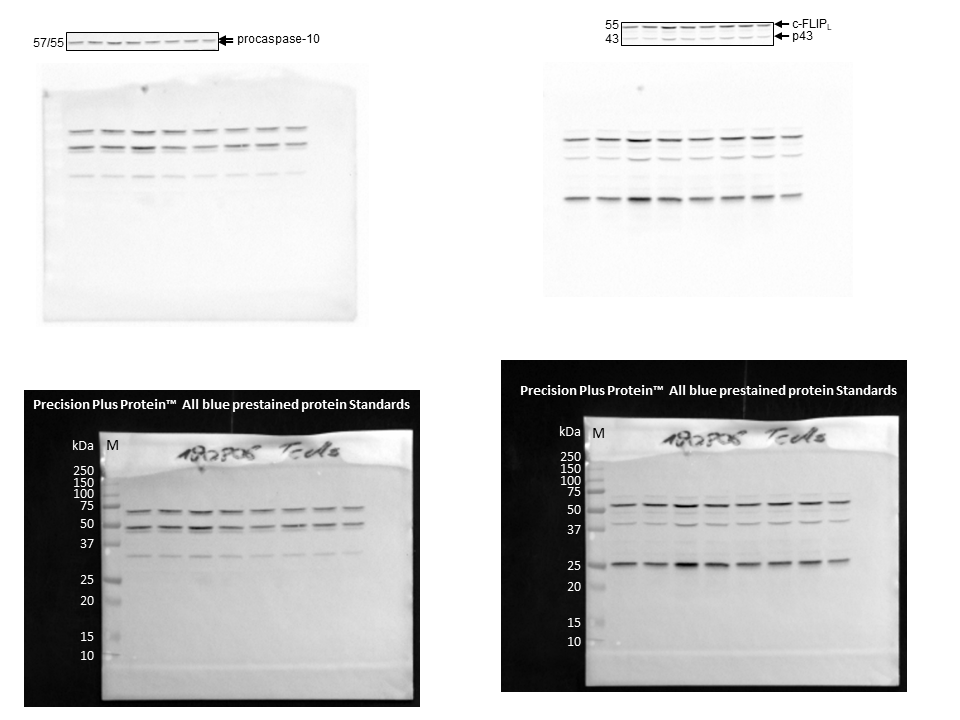

Supplement: Supplementary file 2 [file Presentation2.zip › supplfigure1g-Tcells-rawdata/Folie2.TIF]

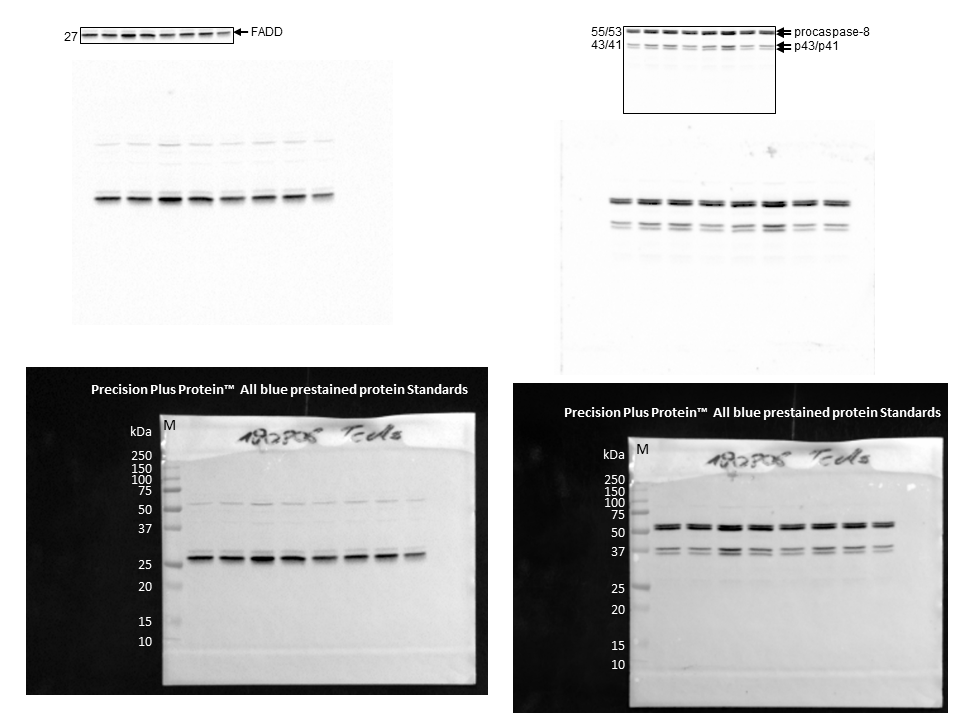

Supplement: Supplementary file 2 [file Presentation2.zip › supplfigure1g-Tcells-rawdata/Folie3.TIF]

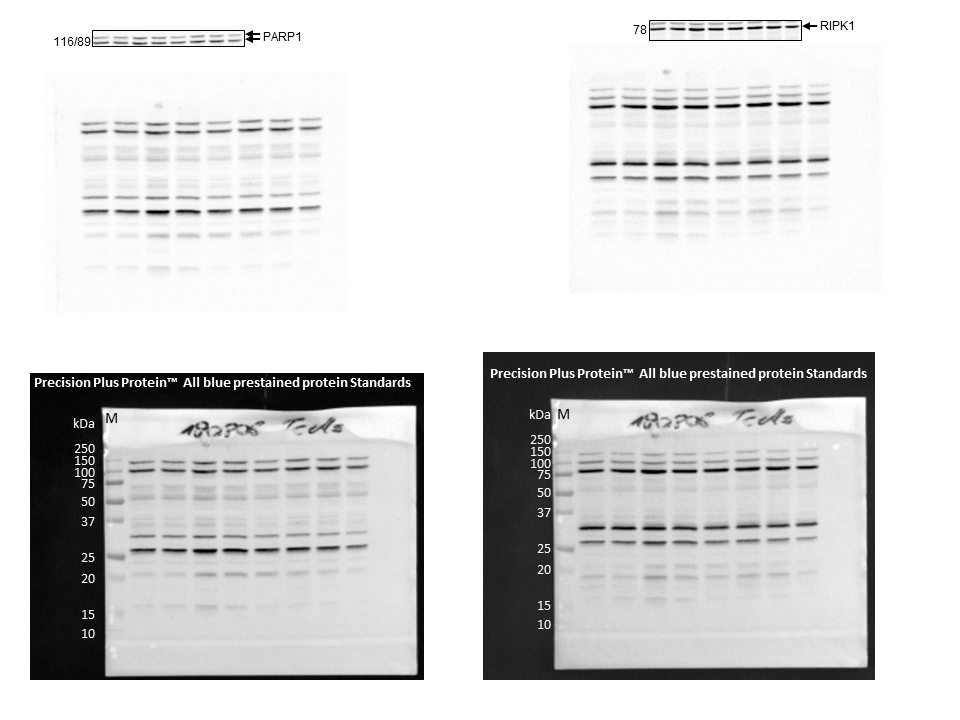

Supplement: Supplementary file 2 [file Presentation2.zip › supplfigure1g-Tcells-rawdata/Folie4.TIF]

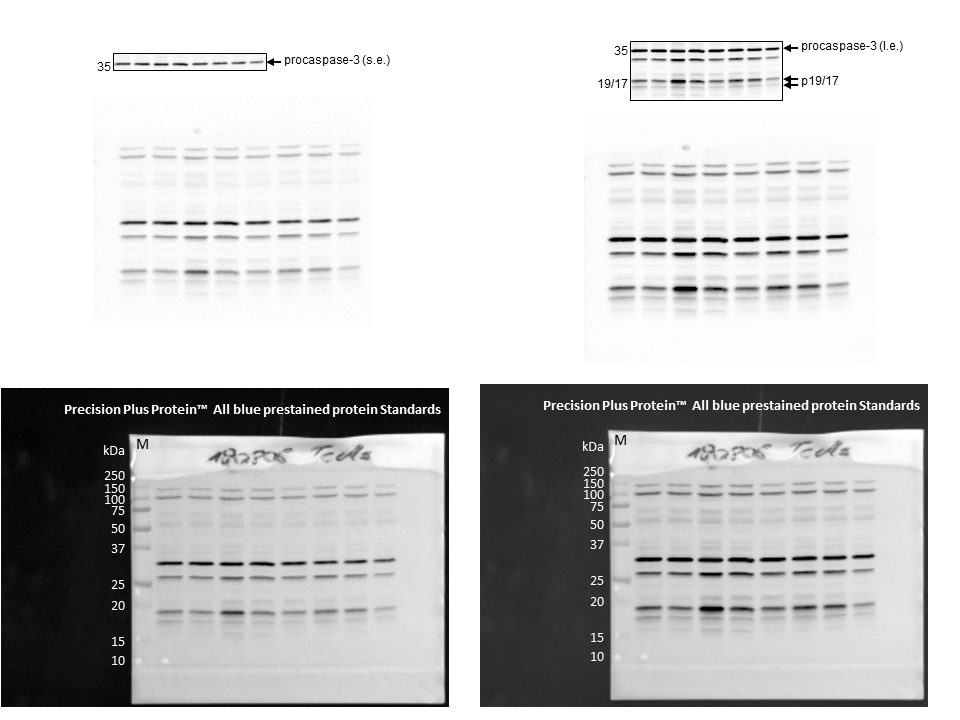

Supplement: Supplementary file 2 [file Presentation2.zip › supplfigure1g-Tcells-rawdata/Folie5.TIF]

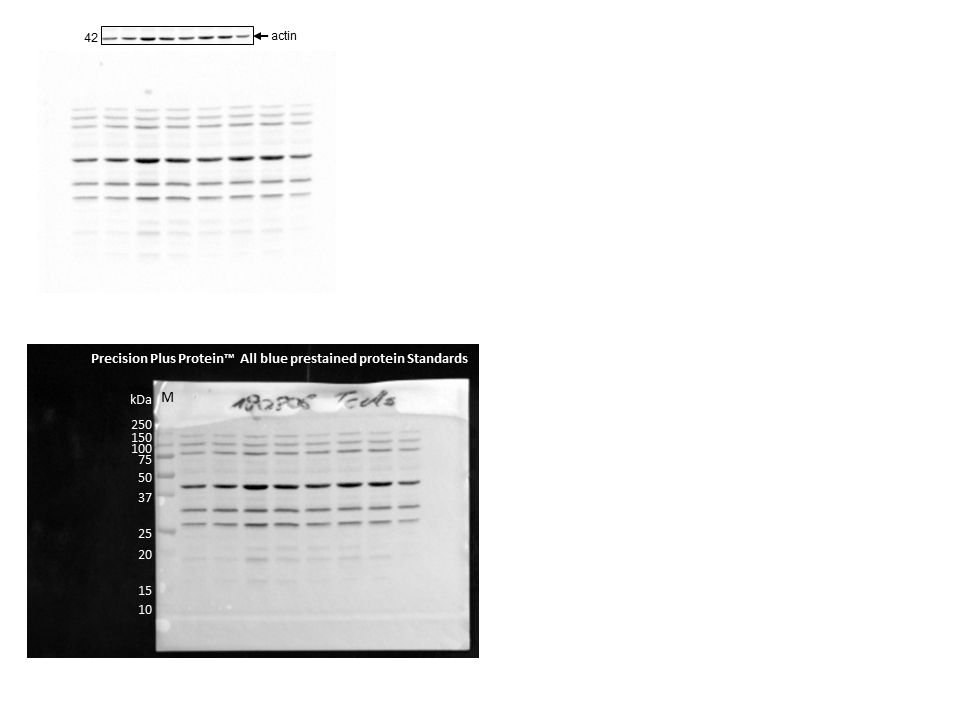

Supplement: Supplementary file 2 [file Presentation2.zip › supplfigure1g-Tcells-rawdata/Folie6.TIF]

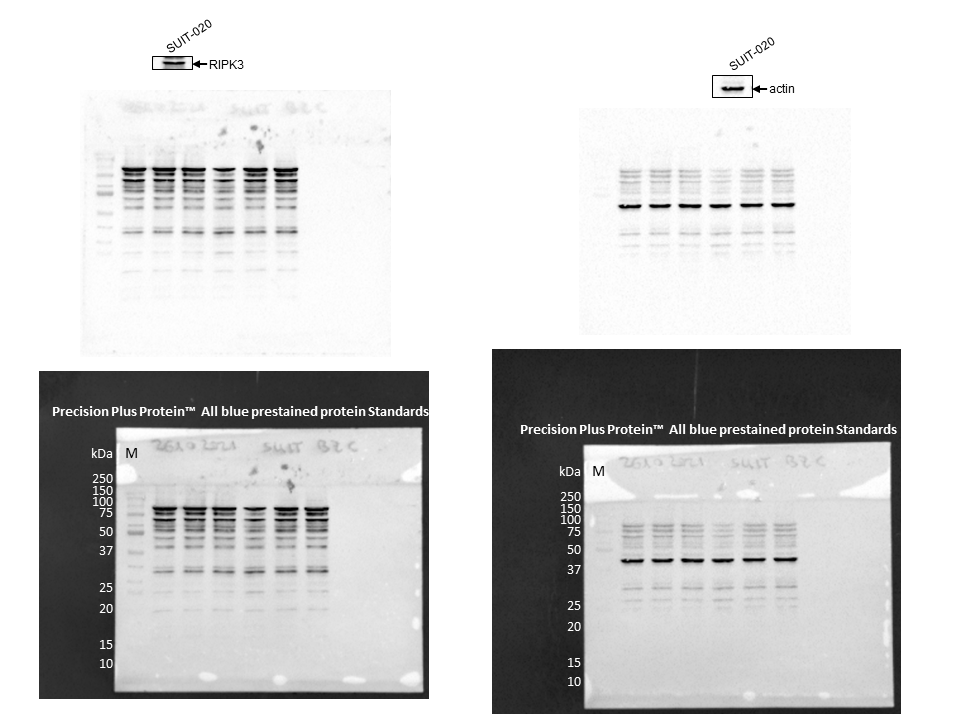

Supplement: Supplementary file 2 [file Presentation2.zip › supplfigure2c-RIPK3blot-rawdata/Folie2.TIF]

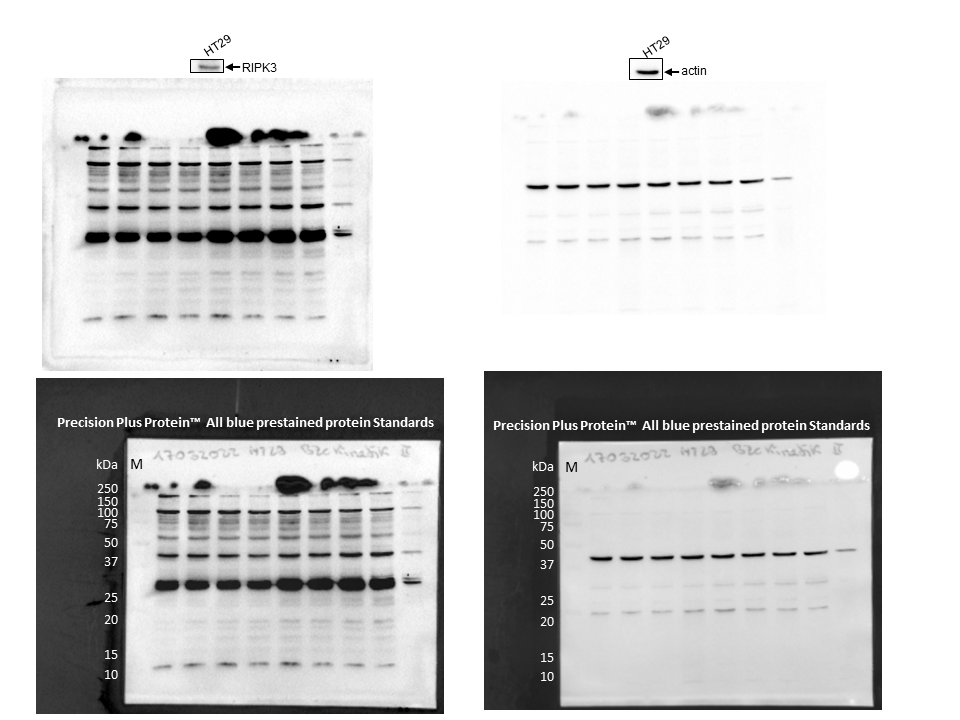

Supplement: Supplementary file 2 [file Presentation2.zip › supplfigure2c-RIPK3blot-rawdata/Folie3.TIF]

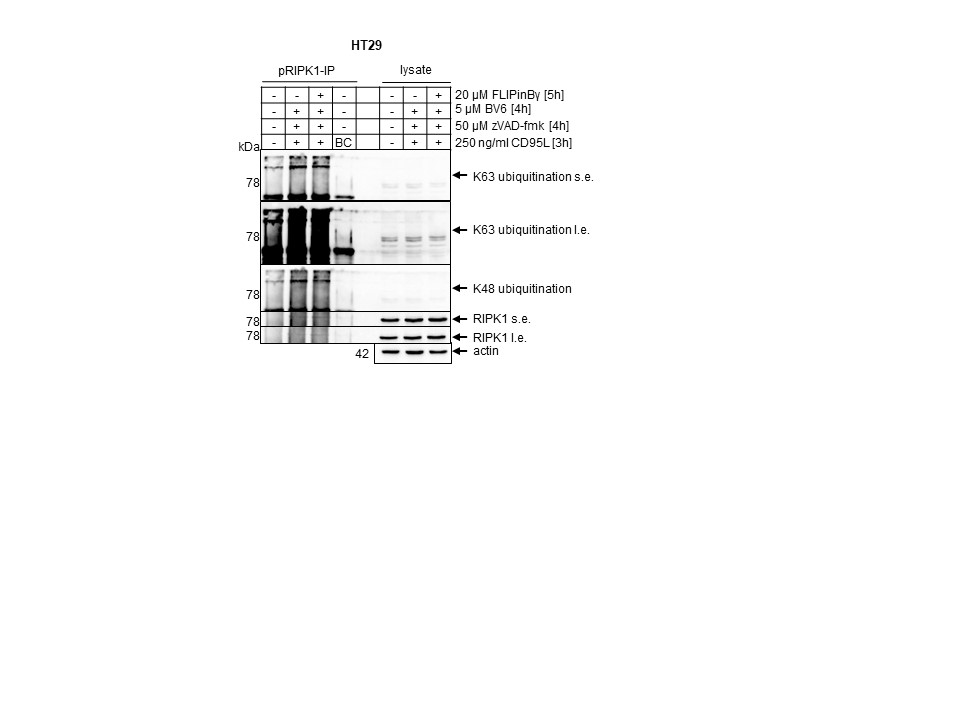

Supplement: Supplementary file 2 [file Presentation2.zip › supplfigure4-HT29-pRIPK1IP-ubi/Folie1.JPG]

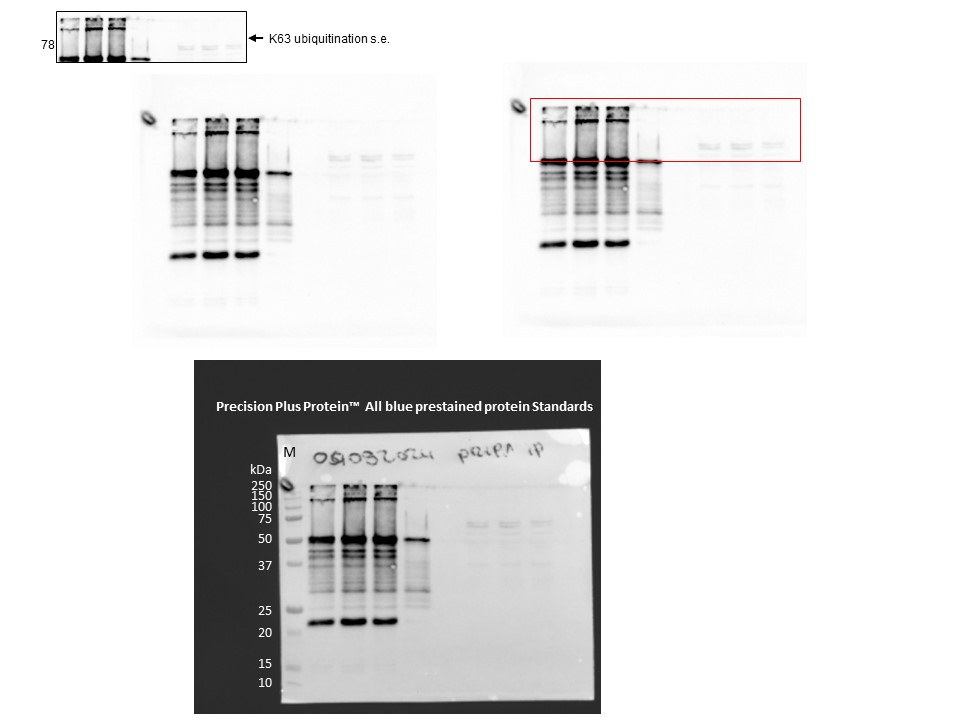

Supplement: Supplementary file 2 [file Presentation2.zip › supplfigure4-HT29-pRIPK1IP-ubi/Folie2.JPG]

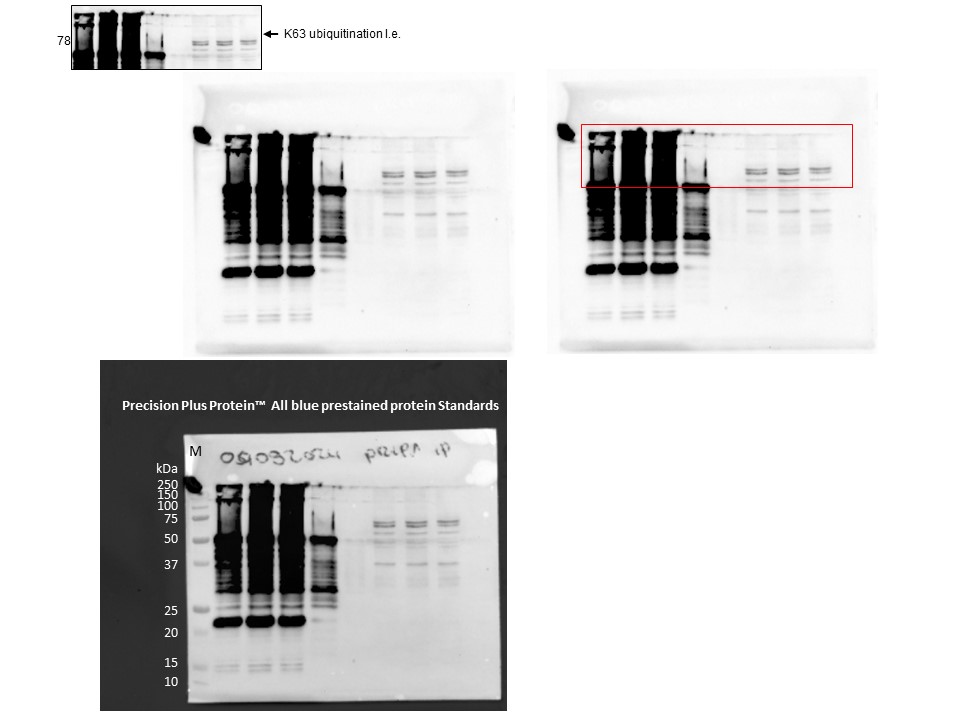

Supplement: Supplementary file 2 [file Presentation2.zip › supplfigure4-HT29-pRIPK1IP-ubi/Folie3.JPG]

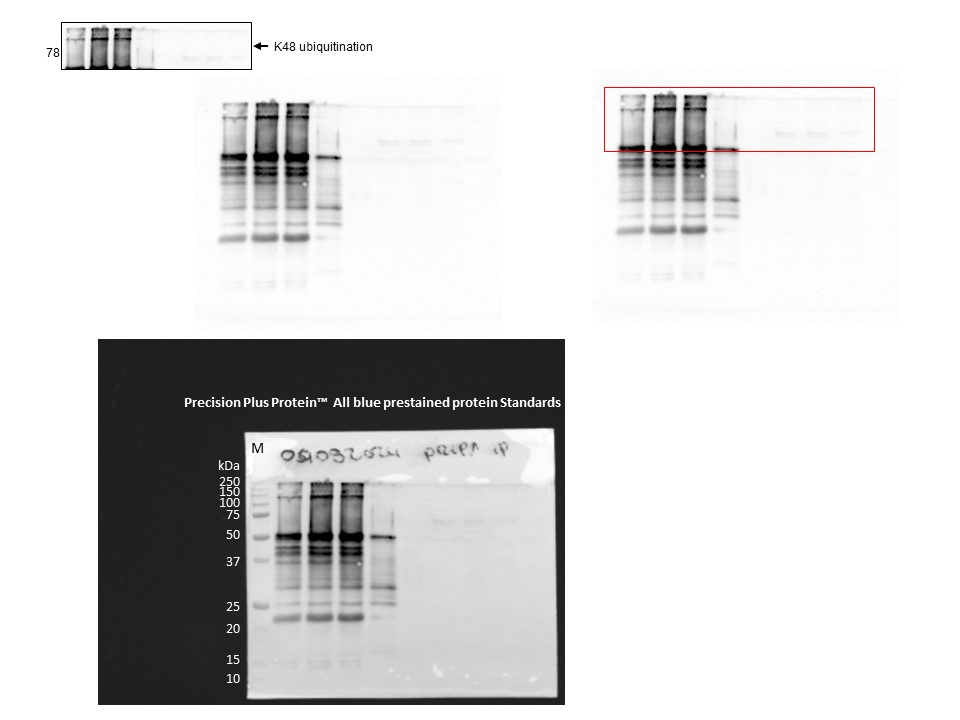

Supplement: Supplementary file 2 [file Presentation2.zip › supplfigure4-HT29-pRIPK1IP-ubi/Folie4.JPG]

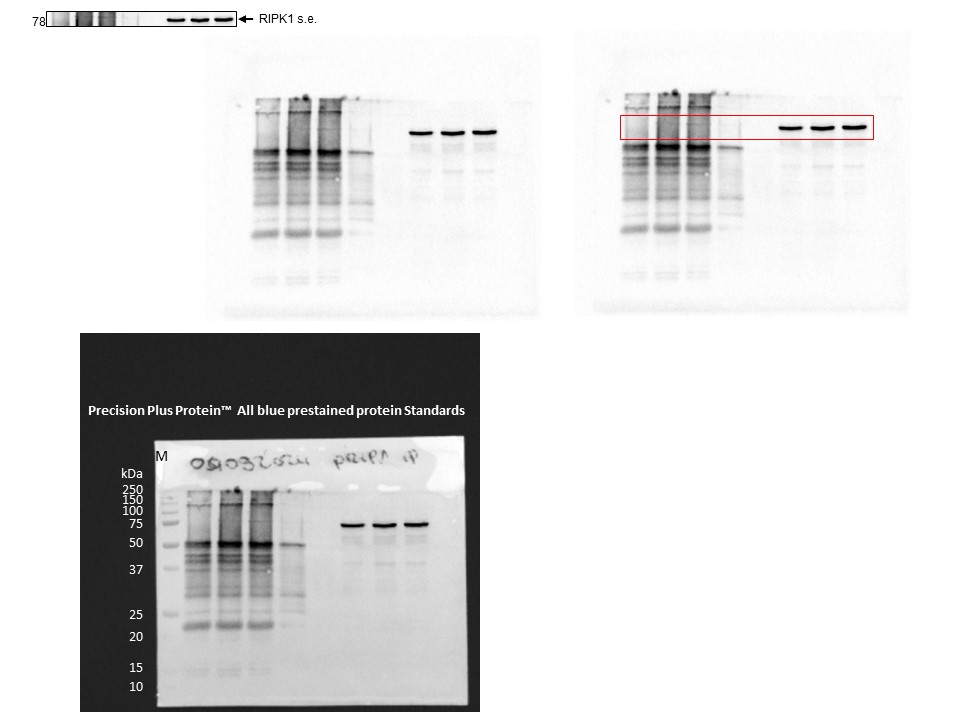

Supplement: Supplementary file 2 [file Presentation2.zip › supplfigure4-HT29-pRIPK1IP-ubi/Folie5.JPG]

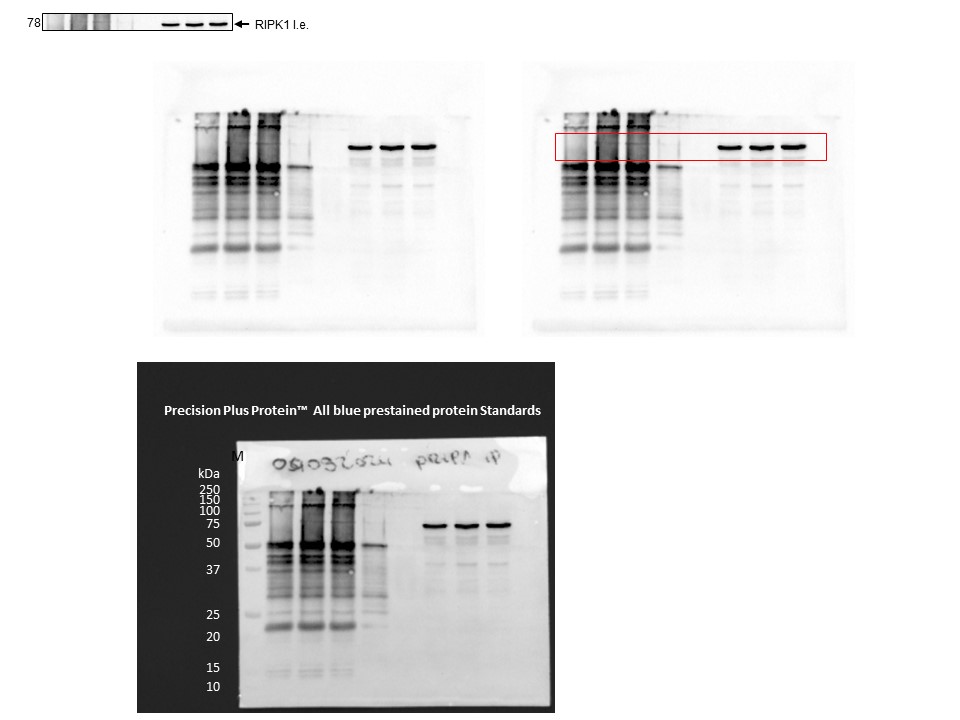

Supplement: Supplementary file 2 [file Presentation2.zip › supplfigure4-HT29-pRIPK1IP-ubi/Folie6.JPG]

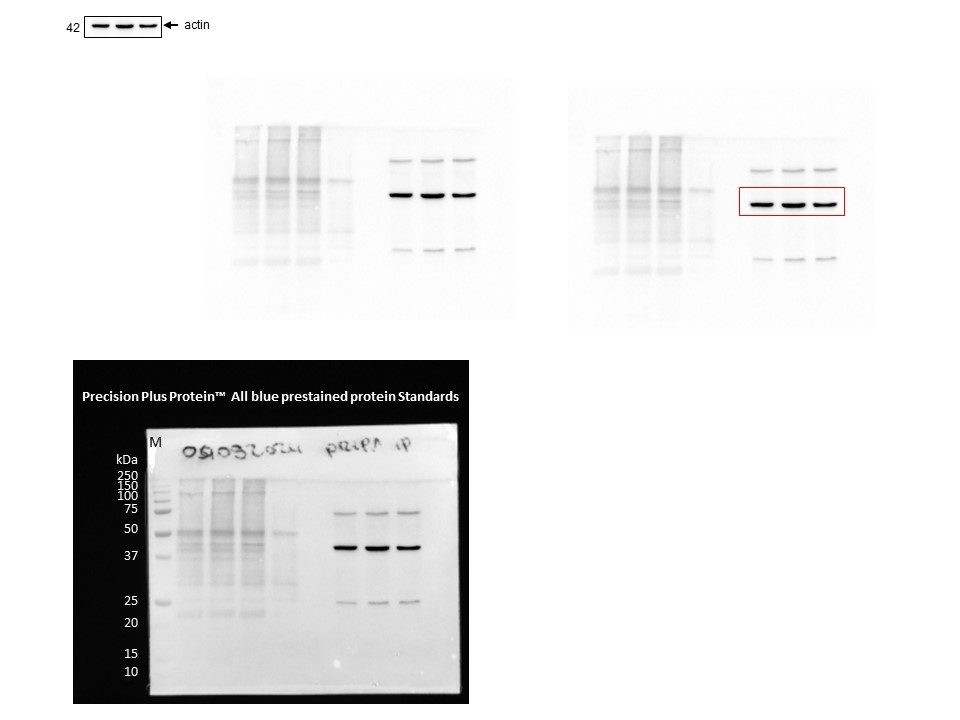

Supplement: Supplementary file 2 [file Presentation2.zip › supplfigure4-HT29-pRIPK1IP-ubi/Folie7.JPG]
